# SLIC-CAGE: high-resolution transcription start site mapping using nanogram-levels of total RNA

**DOI:** 10.1101/gr.235937.118

**Published:** 2018-12

**Authors:** Nevena Cvetesic, Harry G. Leitch, Malgorzata Borkowska, Ferenc Müller, Piero Carninci, Petra Hajkova, Boris Lenhard

**Affiliations:** 1 Institute of Clinical Sciences, Faculty of Medicine, Imperial College London, London W12 0NN, United Kingdom;; 2 MRC London Institute of Medical Sciences, London W12 0NN, United Kingdom;; 3 Institute of Cancer and Genomic Sciences, University of Birmingham, Edgbaston B15 2TT, United Kingdom;; 4 RIKEN Center for Life Science Technologies, Division of Genomic Technologies, Yokohama City, Kanagawa 230-0045, Japan;; 5 RIKEN Omics Science Center, Yokohama City, Kanagawa 230-0045, Japan;; 6 Sars International Centre for Marine Molecular Biology, University of Bergen, N-5008 Bergen, Norway

## Abstract

Cap analysis of gene expression (CAGE) is a methodology for genome-wide quantitative mapping of mRNA 5′ ends to precisely capture transcription start sites at a single nucleotide resolution. In combination with high-throughput sequencing, CAGE has revolutionized our understanding of the rules of transcription initiation, led to discovery of new core promoter sequence features, and discovered transcription initiation at enhancers genome-wide. The biggest limitation of CAGE is that even the most recently improved version (nAnT-iCAGE) still requires large amounts of total cellular RNA (5 µg), preventing its application to scarce biological samples such as those from early embryonic development or rare cell types. Here, we present SLIC-CAGE, a Super-Low Input Carrier-CAGE approach to capture 5′ ends of RNA polymerase II transcripts from as little as 5–10 ng of total RNA. This dramatic increase in sensitivity is achieved by specially designed, selectively degradable carrier RNA. We demonstrate the ability of SLIC-CAGE to generate data for genome-wide promoterome with 1000-fold less material than required by existing CAGE methods, by generating a complex, high-quality library from mouse embryonic day 11.5 primordial germ cells.

Cap analysis of gene expression (CAGE) is used for genome-wide quantitative identification of polymerase II transcription start sites (TSSs) at a single nucleotide resolution (Shiraki et al. 2003) as well as 5′ end-centered expression profiling of RNA polymerase II (RNAPII) transcripts. The region surrounding a TSS (∼40 bp upstream and downstream) represents the core promoter, where the transcription initiation machinery and general transcription factors bind to direct initiation by RNAPII (Smale and Kadonaga 2003). Information on exact TSS positions in the genome improves identification of core promoter sequences and led to the discovery of new core promoter and active enhancer sequences (Andersson et al. 2014; The FANTOM Consortium et al. 2014; Haberle et al. 2014; for review, see Lenhard et al. 2012; Haberle and Lenhard 2016). The current knowledge of core promoter sequences identified by CAGE has uncovered their regulatory role on an unprecedented scale. CAGE-detected TSS profiles represent an accurate and quantitative readout of promoter utilization, and their patterns reflect ontogenic, cell-type–specific and cellular homeostasis-associated dynamic profiles which allows promoter classification and informs about the diversity of promoter-level regulation. This has led to increased use of CAGE techniques and their application in high-profile research projects like ENCODE (The ENCODE Project Consortium 2012), modENCODE (Celniker et al. 2009), FANTOM3 (The FANTOM Consortium and RIKEN Genome Exploration Research Group and Genome Science Group [Genome Network Project Core Group] 2005), and FANTOM5 (The FANTOM Consortium and the RIKEN PMI and CLST [DGT] 2014; Kawaji et al. 2017). Finally, CAGE is proving to be invaluable for uncovering disease-associated novel TSSs (Boyd et al. 2018) that can be used as diagnostic markers, for associating effects of GWAS-identified loci with TSSs (Blauwendraat et al. 2016; Cusanovich et al. 2016), and for facilitating design of CRISPRi experiments (Qi et al. 2013).

Central to CAGE methodology is the positive selection of RNA polymerase II transcripts using the cap-trapper technology (Carninci et al. 1996). This technology uses sodium periodate to selectively oxidize vicinal ribose diols present in the cap structure of mature mRNA transcripts, facilitating their subsequent biotinylation. RNA is first reversely transcribed using a random primer (N_6_TCT) and converted to RNA:cDNA hybrids, followed by oxidation, biotinylation, and treatment with RNase I to select only full-length RNA:cDNA hybrids; i.e., cDNA that has reached the 5′ end of capped mRNA during reverse transcription will protect RNA against digestion with RNase I. Purification of biotinylated RNA:cDNA hybrids is then performed using streptavidin-coated paramagnetic beads. These steps ensure that incompletely synthesized cDNA and cDNA synthesized from uncapped RNAs are eliminated from the sample. The initial CAGE protocol required large amounts of starting material (30–50 µg of total cellular RNA) and used restriction enzyme digestion to generate short reads (20 bp) (Kodzius et al. 2006), whereas the later versions reduced the starting amount 10-fold and generated slightly longer reads (27 bp) with increased mappability (Takahashi et al. 2012).

The latest CAGE protocol using cap-trapping is nAnT-iCAGE (Murata et al. 2014), and it is the most unbiased method for genome-wide identification of TSSs. It excludes PCR amplification as well as restriction enzymes used to produce short reads in previous CAGE versions. However, at least 5 µg of total RNA material are still required for nAnT-iCAGE. To address this, an alternative, biochemically unrelated approach, nanoCAGE, was developed for samples of limited material availability (50–500 ng of total cellular RNA) (Plessy et al. 2010; Poulain et al. 2017). NanoCAGE uses template switching (Zhu et al. 2002) instead of the cap-trapper technology to lower the starting material. Template switching is based on reverse transcriptase's ability to add extra cytosines complementary to the cap, which are then used for hybridization of the riboguanosine-tailed template switching oligonucleotide to extend and barcode only the 5′ full-length cDNAs. Despite its simplicity, nanoCAGE has limitations that make it inferior to classic CAGE protocols: (1) Template switching has been shown to be sequence-dependent and therefore biased (Tang et al. 2013), potentially compromising the determination of preferred TSS positions; and (2) production of libraries from 50 ng of total RNA often requires 20–35 PCR amplification cycles, leading to low-complexity libraries with high levels of duplicates. Although nanoCAGE methodology implements unique molecular identifiers (UMIs) (Kivioja et al. 2012; Poulain et al. 2017), their use for removal of PCR duplicates is often complicated due to problems with achieving truly randomly synthesized UMIs and errors in sequencing (Smith et al. 2017).

Despite improvements in the CAGE methodology, the amount of input RNA needed for unbiased genome-wide identification of TSSs constitutes a true limitation when cells, and therefore RNA, are difficult to obtain. This is the case when working with embryonic tissue or early embryonic stages, rare cell types, FACS-sorted selected cells, heterogeneous tumors, or diagnostic biopsies. Here, we present SLIC-CAGE, a Super-Low Input Carrier-CAGE approach that is based on cap-trapper technology and can generate unbiased high-complexity libraries from 5 to 10 ng of total RNA. Thus far, the cap-trapper step has been the limiting factor in the reduction of the amount of required starting material. To facilitate the cap-trapper technology on the nanogram scale, representing capped RNA from as low as hundreds of eukaryotic cells, samples of the total RNA of interest are supplemented with novel predesigned carrier RNAs. Prior to sequencing, the carrier is efficiently removed from the final library using homing endonucleases that target recognition sites embedded within the sequences of the carrier molecules, leaving only the target mRNA library to be amplified and sequenced.

## Results

### Development of SLIC-CAGE

To enable profiling of minute amounts of RNA, we set out to design a carrier RNA similar in size distribution and percentage of capped RNA to the cellular RNA, but whose cDNA will selectively degrade without affecting the cDNA originating from the sample.

**Figure 1.**
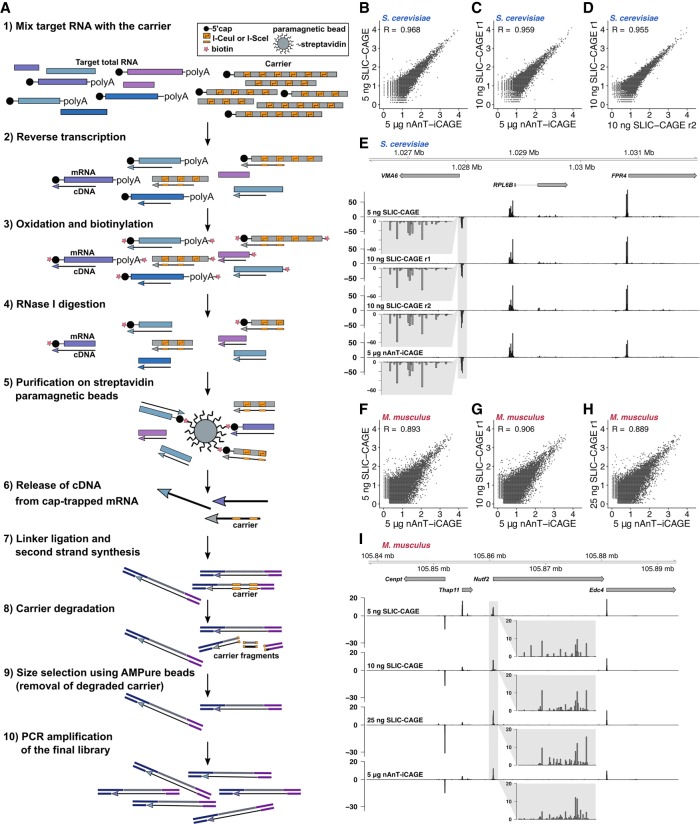
SLIC-CAGE development and assessment. (*A*) Schematics of the SLIC-CAGE approach. Target RNA of limited quantity is mixed with the carrier mix to get 5 µg of total RNA material. cDNA is synthesized through reverse transcription and cap is oxidized using sodium periodate. Oxidation allows attachment of biotin using biotin hydrazide. In addition to the cap structure, biotin gets attached to the mRNA's 3′ end, as it is also oxidized using sodium periodate. To remove biotin from mRNA:cDNA hybrids with incompletely synthesized cDNA, and from mRNA's 3′ ends, the samples are treated with RNase I. Complete cDNAs (cDNA that reached the 5′ end of mRNA) are selected by affinity purification on streptavidin magnetic beads (cap-trapping). cDNA is released from cap-trapped cDNA:mRNA hybrids and 5′- and 3′-linkers are ligated. The library molecules that originate from the carrier are degraded using I-Sce-I and I-Ceu-I homing endonucleases and the fragments removed using AMPure beads. The leftover library molecules are then PCR-amplified to increase the amount of material for sequencing. (*B*,*C*) Pearson's correlation at the CTSS level of nAnT-iCAGE and SLIC-CAGE libraries prepared from (*B*) 5 ng or (*C*) 10 ng of *S. cerevisiae* total RNA. (*D*) Pearson's correlation at the CTSS level of SLIC-CAGE technical replicates prepared from 10 ng of *S. cerevisiae* total RNA. The axes in *B*–*D* show log_10_(TPM+1) values and the correlation was calculated on raw, non-log-transformed data. (*E*) CTSS signal in example locus on Chromosome 12 in SLIC-CAGE libraries prepared from 5 or 10 ng of *S. cerevisiae* total RNA and in nAnT-iCAGE library prepared from standard 5 µg of total RNA. The *inset* gray boxes show a magnification of a tag cluster. (*F*–*H*) Pearson's correlation at the CTSS level of nAnT-iCAGE and SLIC-CAGE libraries prepared from (*F*) 5 ng, (*G*) 10 ng, or (*H*) 25 ng of *M. musculus* total RNA. The axes in *F*–*H* show log_10_(TPM+1) values and the correlation was calculated on raw non-log-transformed data. (*I*) CTSS signal in example locus on Chromosome 8 in SLIC-CAGE libraries prepared from 5, 10, or 25 ng of *M. musculus* total RNA and in the reference nAnT-iCAGE library prepared from standard 5 µg of total RNA. The *inset* gray boxes show a magnification of a tag cluster, and one bar represents a single CTSS.

We constructed the synthetic gene used as a template for run-off in vitro transcription of the carrier RNA (Supplemental Fig. S1A,B; Supplemental Table S1; see Methods for details). The synthetic gene is based on the *Escherichia coli* leucyl-tRNA synthetase sequence for two main reasons. First, we wanted to avoid mapping to eukaryotic genomes. Secondly, leucyl-tRNA synthetase is a housekeeping gene from a mesophilic species, and therefore its sequence is not expected to form strong secondary structures that would reduce its translation in vivo or reduce the efficiency of reverse transcription. We made this carrier selectively degradable by embedding it with multiple recognition sites of two homing endonucleases, I-CeuI and I-SceI (Fig. 1A; Supplemental Figs. S1A,B, S20; Supplemental Table S1). Combination of alternating recognition sites allows for higher degradation efficiency and reduces sequence repetitiveness. The two enzymes have recognition sites of lengths 27 and 18 bp, respectively, which even with some degeneracy allowed in the recognition site (Gimble and Wang 1996; Argast et al. 1998) makes their random occurrence in a transcriptome highly improbable. The two enzymes work at the same temperature and in the same buffer, so their digestion can be combined in a single step. A fraction of the synthesised carrier RNA is capped using the Vaccinia Capping System (NEB) and mixed with uncapped carrier to achieve the desired capping percentage (Palazzo and Lee 2015).

The percentage of capped RNAs in the carrier and its size distribution were optimized by performing the entire SLIC-CAGE protocol, starting by adding the synthetic carrier to the low-input sample to achieve a total of 5 µg of RNA material. To assess its performance, we compared its output with the nAnT-iCAGE library derived from 5 µg of total cellular RNA. We use nAnT-iCAGE as a reference as it is currently considered the most unbiased protocol for promoterome mapping (Murata et al. 2014) and because TSS identification by cap-trapper-based technology has been experimentally validated (Carninci et al. 2006). To identify the optimal ratio of capped and uncapped carrier, as well as the length of the carrier RNAs, we tested the following carrier mixes: (1) carriers with lengths distributed between 0.3 and 1 kb versus homogenous 1kb-length carriers; and (2) a mixture of capped and uncapped versus only capped carrier. We performed the SLIC-CAGE protocol outlined in Figure 1A, starting with 100 ng of total RNA isolated from *Saccharomyces cerevisiae* supplemented with the various carrier mixes up to a total of 5 µg of RNA. We then compared the output with the nAnT-iCAGE library generated using 5 µg of total RNA (Supplemental Fig. S1C–M; Supplemental Tables S4, S5; see Methods for more details). Removal of the carrier was performed by two rounds of degradation using homing endonucleases (I-SceI and I-CeuI) (Supplemental Fig. S20) with a purification and a PCR amplification step between the rounds (see Methods for details). The presence of the carrier significantly improved the correlation of individual CAGE-supported TSSs (CTSSs) (see Methods) between SLIC-CAGE and the reference nAnT-iCAGE (Supplemental Fig. S1C–H). This effect was not observed when either only the capped carrier or no carrier was used (Supplemental Fig. S1C,G,H). The highest correlation and reproducibility was achieved by a carrier mix composed of 10% capped and 90% uncapped molecules of 0.3–1 kb length (Supplemental Fig. S1E,F, mix 2; Supplemental Tables S4, S5). This mix was designed to closely mimic the composition of cellular total RNA (see Methods for more details). Other diagnostic criteria shown in Supplemental Figure S1, J–M, confirm it as the optimal carrier choice.

### SLIC-CAGE allows genome-wide TSS identification from nanogram-scale samples

We set out to identify the lowest amount of total RNA that can be used to produce high-quality CAGE libraries. To that end, we performed a SLIC-CAGE titration test with 1–100 ng of total *S. cerevisiae* RNA and compared this with nAnT-iCAGE library derived from 5 µg of total RNA. The high correlation of individual CTSSs between SLIC-CAGE and the reference nAnT-iCAGE library (Fig. 1B,C; Supplemental Fig. S2A) shows that genuine CTSSs are identified. Moreover, SLIC-CAGE libraries show high reproducibility (Fig. 1D). Figure 1E shows an example locus in the genome browser, demonstrating the high similarity of SLIC-CAGE and nAnT-iCAGE CTSS profiles in all high-quality data sets (i.e., data sets with high complexity; see below).

To confirm the general applicability of the SLIC-CAGE protocol, we performed a similar titration test using total RNA isolated from E14 mouse embryonic stem cells. The results obtained following sequencing of the libraries generated using 5, 10, or 25 ng of total RNA were highly correlated (Pearson's correlation 0.9) with the reference nAnT-iCAGE-derived library. The correlation did not improve further with increasing total starting RNA (Fig. 1F–H; Supplemental Fig. S2C), again verifying the SLIC-CAGE protocol for nanogram-scale samples. The genome browser view (Fig. 1I) confirms the similarity of profiles on the individual CTSS level, although with minor differences in the library prepared from 5 ng of *Mus musculus* total RNA due to lower complexity, as discussed in detail in the next section. Analysis of library mapping efficiency demonstrated that selective degradation of the carrier is highly efficient (see Supplemental Material; Supplemental Tables S10, S11).

### Complexity and resolution of SLIC-CAGE libraries

**Figure 2.**
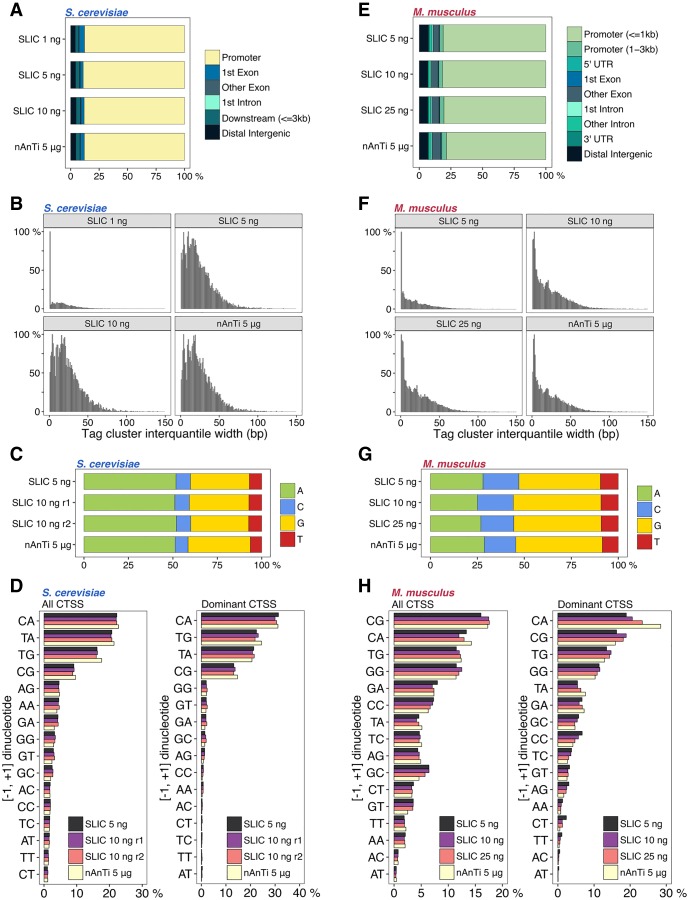
Identifying the lower limits of SLIC-CAGE libraries. (*A*) Genomic locations of tag clusters identified in SLIC-CAGE libraries prepared from 1, 5, or 10 ng of *S. cerevisiae* total RNA versus the reference nAnT-iCAGE library. (*B*) Distribution of tag cluster inter-quantile widths in SLIC-CAGE libraries prepared from 1, 5, or 10 ng of *S. cerevisiae* total RNA and in the nAnT-iCAGE library. (*C*) Nucleotide composition of all CTSSs identified in SLIC-CAGE libraries prepared from 5 or 10 ng of *S. cerevisiae* total RNA and in the reference nAnT-iCAGE library. (*D*) Dinucleotide composition of all CTSSs (*left* panel) or dominant CTSSs (*right* panel) identified in SLIC-CAGE libraries prepared from 5 or 10 ng of *S. cerevisiae* total RNA and in the nAnT-iCAGE library. Both panels are ordered from the most to the least used dinucleotide in nAnT-iCAGE. (*E*) Genomic locations of tag clusters in SLIC-CAGE libraries prepared from 5, 10, or 25 ng of *M. musculus* total RNA and in the nAnT-iCAGE library. (*F*) Distribution of tag cluster inter-quantile widths in SLIC-CAGE libraries prepared from 5, 10, or 25 ng of *M. musculus* total RNA and the nAnT-iCAGE library. (*G*) Nucleotide composition of all CTSSs identified in SLIC-CAGE libraries prepared from 5, 10, or 25 ng of *M. musculus* total RNA or identified in the nAnT-iCAGE library. (*H*) Dinucleotide composition of all CTSSs (*left* panel) or dominant CTSSs (*right* panel) identified in SLIC-CAGE libraries prepared from 5, 10, or 25 ng of *M. musculus* total RNA or identified in the reference nAnT-iCAGE library. Both panels are ordered from the most to the least used dinucleotide in the reference nAnT-iCAGE.

Next, we wanted to explore library complexity and any potential inherent CTSS detection biases. CTSSs in close vicinity reflect functionally equivalent transcripts and are generally clustered together and analyzed as a single transcriptional unit termed a tag cluster (Haberle et al. 2015). The CTSS with the highest TPM value within a tag cluster is referred to as the *dominant* CTSS. Specificity in capturing genuine TSSs can be assessed by examining the fraction of tag clusters that overlap with expected promoter regions. We identified a high percentage of SLIC-CAGE tag clusters that map to known promoter regions in both *S. cerevisiae* and *M. musculus* libraries irrespective of the total starting RNA, thus indicating the high specificity of these libraries (∼80%, at the same level as the reference nAnT-iCAGE protocol) (Fig. 2A,E; Supplemental Fig. S2D,F).

In addition to determining the number of unique detected CTSSs and tag clusters and their overlap with the reference library (Supplemental Table S12), complexity of CAGE-derived libraries can be assessed by comparing tag cluster widths. To robustly identify tag cluster widths, the inter-quantile widths (IQ-widths) were calculated that span the 10th and the 90th percentile (q0.1–q0.9) of the total tag cluster signal to exclude effects of extreme outlier CTSSs. The distribution of tag cluster IQ-widths serves as a good visual indicator of library complexity, as in low-complexity libraries, incomplete CTSS detection will lead to artificially sharp tag clusters. This low-complexity effect can be simulated by randomly subsampling a high-complexity nAnT-iCAGE library (see Supplemental Fig. S3). IQ-width distribution of *S. cerevisiae* SLIC-CAGE tag clusters reveals that complexity of the reference nAnT-iCAGE library is recapitulated using as little as 5 ng of total RNA (Fig. 2B; Supplemental Fig. S4A). This result is substantiated with the number of unique CTSSs, which corresponds to the number identified with nAnT-iCAGE (around 70% overlap between 5 ng SLIC-CAGE and nAnTi-iCAGE and 90% overlap in tag cluster identification). Low-complexity with artificially sharper tag clusters is seen only with 1–2 ng of total RNA input (Fig. 2B; Supplemental Fig. S4A; Supplemental Table S12). A highly similar result is observed with *M. musculus* SLIC-CAGE libraries, although lower complexity is notable at 5 ng of total RNA (Fig. 2F; Supplemental Fig. S4B). This is in agreement with the lower number of unique CTSSs identified in 5 ng *M. musculus* SLIC-CAGE library compared to nAnT-iCAGE (Supplemental Table S12). We expect that an increase in sequencing depth would ultimately recapitulate the complexity of the reference data set, as higher coverage in *S. cerevisiae* facilitates higher complexity libraries with a lower starting amount (5 ng).

To assess sensitivity and precision of SLIC-CAGE, we used standard RNA-seq receiver operating characteristic (ROC) curves with true CTSSs and tag clusters defined by nAnT-iCAGE and show similar ratios of true and false positives when identifying CTSSs or tag clusters in high-complexity libraries, regardless of total RNA input amount (Supplemental Fig. S5A,C).

We also assessed SLIC-CAGE-derived CTSS features from *S. cerevisiae* and *M. musculus* and compared them with features extracted using nAnT-iCAGE as a reference. First, nucleotide composition of all SLIC-CAGE-identified CTSSs reveals highly similar results to nAnT-iCAGE, independent of the total input RNA (Fig. 2C,G; Supplemental Fig. S2G,I). Furthermore, the composition of [−1,+1] dinucleotide initiators (where the +1 nucleotide represents the identified CTSS) also showed a highly similar pattern to the reference nAnT-iCAGE (Fig. 2D,H left panel; Supplemental Fig. S2J,L). SLIC-CAGE libraries identify CA, TA, TG, and CG as the most preferred initiators, similar to preferred mammalian initiator sequences (Carninci et al. 2006).

Focusing only on the initiation patterns ([−1, +1] dinucleotide) of the dominant TSS (CTSSs with the highest TPM within each tag cluster) of each tag cluster facilitates estimating the influence of PCR amplification on the distribution of tags within a tag cluster. Highly similar dinucleotide composition of dominant TSS initiators, independent of the amount of total RNA used, confirms that identification of the dominant TSSs is not obscured by PCR amplification (Fig. 2D,H, right panel; Supplemental Fig. S2M,O). The identified preferred initiators are pyrimidine-purine dinucleotides CA, TG, TA (*S. cerevisiae*) or CA, CG, TG (*M. musculus*) in accordance with the Inr element (YR) (Burke and Kadonaga 1997; Haberle and Lenhard 2016). These results confirmed the utility of SLIC-CAGE in uncovering authentic transcription initiation patterns such as the well-established CA initiator.

Further, we analyzed the distance between dominant TSSs identified in tag clusters common to each SLIC-CAGE and the reference nAnT-iCAGE sample and show (1) the same dominant TSS is identified in 50%–60% cases in high-complexity libraries (≥10 ng of total RNA), and (2) 75%–80% of identified dominant TSSs are within a 10-bp distance of the nAnT-iCAGE-identified dominant TSS in high-complexity libraries (Supplemental Figs. S6, S7; Supplemental Table S12). Some variability between the identified dominant TSSs is expected, even between technical nAnT-iCAGE replicates, especially in broad tag clusters with several TSSs of similar expression level (70% of dominant TSSs within a 10-bp distance of nAnT-iCAGE technical replicates) (Supplemental Table S13; Supplemental Fig. S8).

As a final assessment of SLIC-CAGE performance, we analyzed expression ratios per individual CTSS common to SLIC-CAGE and the reference nAnT-iCAGE (Supplemental Figs. S9, S10, left panels) and present the ratios in a heat map centered on the dominant CTSS identified by the reference nAnT-iCAGE library. This analysis can uncover any positional biases if introduced by the SLIC-CAGE protocol. Patterns of signal in heat maps (grouping upstream of or downstream from the nAnT-iCAGE-identified dominant CTSS) would signify positional bias and indicate nonrandom capturing of authentic TSSs. We also evaluated the positions and expression values of CTSSs identified in the nAnT-iCAGE but absent in SLIC-CAGE libraries (Supplemental Figs. S9, S10, middle panels). We found there are no positional biases with regard to SLIC-CAGE-identified CTSSs and their expression values, independent of the total input RNA. As expected, a higher number of CTSSs identified in nAnT-iCAGE were absent from lower complexity *S. cerevisiae* SLIC-CAGE libraries derived from 1 and 2 ng total RNA (Supplemental Fig. S9A,B, middle panels). This was particularly evident in those CTSSs with expression values in the lower two quartiles (top two sections in each heat map). Further, the CTSSs identified in both low-complexity SLIC-CAGE and nAnT-iCAGE exhibit higher TPM ratios, likely reflecting the effect of PCR amplification. On the other hand, we found that the SLIC-CAGE library derived from 5 ng of total RNA (Supplemental Fig. S9C) shows similar patterns as libraries derived from greater amounts of RNA (Supplemental Fig. S9D–H) or the library derived by PCR amplification of the nAnT-iCAGE library (Supplemental Fig. S9I).

Similar results were obtained when comparing *M. musculus* SLIC-CAGE libraries with their reference nAnT-iCAGE library (Supplemental Fig. S10), albeit with a twofold greater minimum starting RNA (10 ng) required for high-complexity libraries. Overall, these results show that SLIC-CAGE increases the sensitivity of the CAGE method 1000-fold over the current “gold standard” nAnT-iCAGE, without decrease in signal quality.

### SLIC-CAGE generates superior quality libraries compared to existing low-input methods

**Figure 3.**
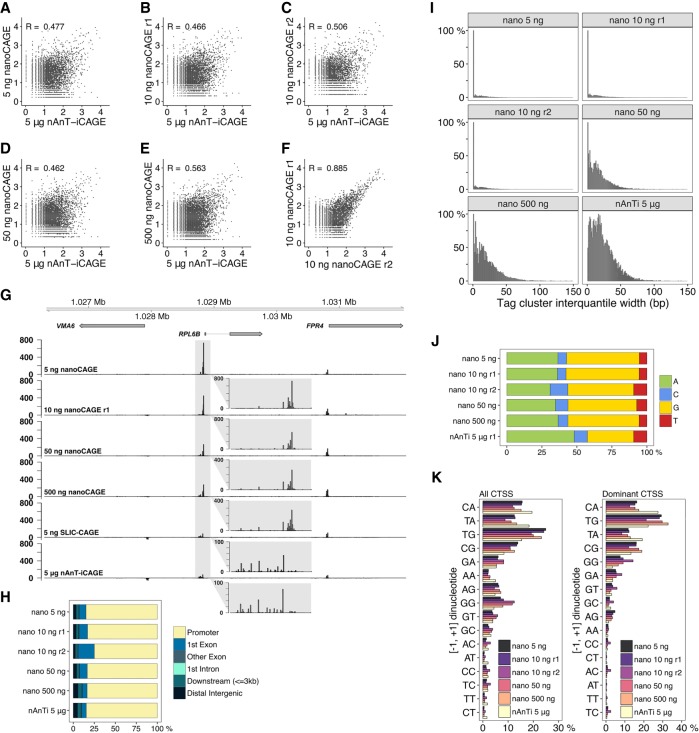
Comparison of nanoCAGE and the reference nAnT-iCAGE. (*A*–*E*) Pearson's correlation of nAnT-iCAGE and nanoCAGE libraries prepared from (*A*) 5 ng, (*B*,*C*) 10 ng, (*D*) 50 ng, or (*E*) 500 ng of *S. cerevisiae* total RNA. (*F*) Pearson's correlation of nanoCAGE technical replicates prepared from 10 ng of *S. cerevisiae* total RNA. (*G*) CTSS signal in example locus on Chromosome 12 in nanoCAGE libraries prepared from 5, 10, 50, or 500 ng, SLIC-CAGE library prepared from 5 ng and the nAnT-iCAGE library prepared from 5 µg of *S. cerevisiae* total RNA (the same locus is shown in Fig. 1E). The *inset* gray boxes show a magnification of a tag cluster. *Insets* in nanoCAGE libraries have a different scale, as signal is skewed with PCR amplification. Different tag cluster is magnified compared to Figure 1E, as nanoCAGE did not detect the upstream tag cluster on the minus strand. Additional validation is presented in Supplementary Figure S22. (*H*) Genomic locations of tag clusters identified in nanoCAGE libraries prepared from 5 to 500 ng of *S. cerevisiae* total RNA and in the nAnT-iCAGE library. (*I*) Distribution of tag cluster inter-quantile widths in nanoCAGE libraries prepared from 5 to 500 ng of *S. cerevisiae* total RNA versus the reference nAnT-iCAGE library. (*J*) Nucleotide composition of all CTSSs identified in nanoCAGE libraries prepared from 5 to 500 ng of *S. cerevisiae* total RNA or identified in the reference nAnT-iCAGE library. (*K*) Dinucleotide composition of all CTSSs (*left* panel) or dominant CTSSs (*right* panel) identified in nanoCAGE libraries prepared from 5 to 500 ng of *S. cerevisiae* total RNA or in the reference nAnT-iCAGE library. Both panels are ordered from the most to the least used dinucleotide in the reference nAnT-iCAGE.

We first set off to compare nanoCAGE against nAnT-iCAGE. We carried out a nanoCAGE titration test using *S. cerevisiae* total RNA (5–500 ng) and compared the obtained libraries with the reference nAnT-iCAGE library. CTSSs identified in nanoCAGE libraries were poorly correlated (Pearson's correlation 0.5–0.6) with the nAnT-iCAGE library, irrespective of the total RNA used (Fig. 3A–E; Supplemental Fig. S2B). Despite reduced similarity with nAnT-iCAGE, nanoCAGE libraries appeared reproducible (Fig. 3F). An example genome browser view also reveals significant differences in CTSS profiles between nanoCAGE and nAnT-iCAGE libraries (Fig. 3G). NanoCAGE systematically failed to capture all CTSSs identified with nAnT-iCAGE. In contrast, our SLIC-CAGE library derived from only 5 ng of total RNA accurately recapitulates the nAnT-iCAGE TSS profile shown in the same genomic region (Fig. 3G as in Fig. 1E).

Next, we investigated the tag clusters identified in each nanoCAGE library and showed that ∼85% were indeed in expected promoter regions (Fig. 3H; Supplemental Fig. S2E). The cluster overlap is highly similar to the reference nAnT-iCAGE library in all nanoCAGE libraries, independent of the amount of total RNA used. Therefore, nanoCAGE does not capture the full complexity of TSS usage, but its specificity for promoter regions is not diminished.

To inspect the complexity of nanoCAGE libraries, we again compared tag cluster IQ-widths with the reference nAnT-iCAGE library (Fig. 3I; Supplemental Fig. S4C). An increase in the number of sharper tag clusters is observed at 1–50 ng of total input RNA. The IQ-width distributions show that nanoCAGE systematically produces lower-complexity libraries compared to nAnT-iCAGE and SLIC-CAGE. This result agrees well with the consistently lower number of unique CTSSs identified in nanoCAGE libraries and its low overlap with nAnT-iCAGE CTSSs (Supplemental Table S13).

To further assess performance of nanoCAGE, we used ROC curves with true CTSSs or tag clusters defined by the nAnT-iCAGE library (Supplemental Fig. S5C). SLIC-CAGE substantially outperforms nanoCAGE—in nanoCAGE, the ratio of true and false positives is low and highly dependent on total RNA input amount.

Nucleotide composition of nanoCAGE-identified robust CTSSs revealed a strong preference for G-containing CTSSs (Fig. 3J). This observed G-preference is not an artifact caused by the extra C added complementary to the cap structure at the 5′ end of cDNA during reverse transcription, as that is common to all CAGE protocols and corrected using the Bioconductor package *CAGEr* (Haberle et al. 2015). To check if in nanoCAGE more than one G is added during reverse transcription, we counted the 5′ end Gs flagged as a mismatch in the alignment and found that the amount of two consecutive mismatches was not significant (Supplemental Table S14).

We also analyzed the distance between dominant TSSs identified in tag clusters common to each nanoCAGE and the reference nAnT-iCAGE sample and show that (1) nanoCAGE captures only 30% of the dominant TSSs regardless of the RNA input quantity, and (2) only about 60% of identified dominant TSSs are within a 10-bp distance of the nAnT-iCAGE-identified dominant TSS regardless of the RNA input (Supplemental Fig. S8; Supplemental Table S13). Taken together, the results demonstrate that SLIC-CAGE strongly outperforms nanoCAGE in identification of true dominant TSSs.

The composition of [−1,+1] initiator dinucleotides revealed a severe depletion in identified CA and TA initiators, with the corresponding increase in G-containing initiators (TG and CG), in comparison with the reference nAnT-iCAGE data set (Fig. 3K, left panel; Supplemental Fig. S2K). To assess the most robust CTSSs, we repeated the same analysis using only the dominant CTSSs in each tag cluster (Fig. 3K, right panel; Supplemental Fig. S2N), and the lack of CA and TA initiators was equally apparent. This property of nanoCAGE makes it unsuitable for the determination of dominant CTSSs and details of promoter architecture at base-pair resolution.

To exclude the effects of CTSSs located in nonpromoter regions and to assess if CTSS identification depends on expression levels, we divided tag clusters according to their genomic location or expression values (division into four expression quartiles per each library) and repeated the analysis (Supplemental Figs. S11, S12). Since a similar pattern (depletion of CA and TA initiators) was observed irrespective of the genomic location or expression level, these results suggest that the nanoCAGE bias is caused by template switching of reverse transcriptase known to be sequence-dependent and expected to preferentially capture capped RNA that starts with G (Zajac et al. 2013).

Finally, we analyzed signal ratios of individual CTSSs identified in each nanoCAGE library and the reference nAnT-iCAGE (ratio of TPM values) (Supplemental Fig. S13, left panels) and CTSSs not identified in nanoCAGE (Supplemental Fig. S13, middle panels) similarly as described for SLIC-CAGE (see above). This analysis reveals that there are no position-specific biases in nanoCAGE and that the biases are primarily caused by nucleotide composition of the capped RNA 5′ ends. Further, it accentuates the inability of nanoCAGE to capture dominant CTSSs identified with the reference nAnT-iCAGE, even with higher amounts of starting material, compared to SLIC-CAGE (Supplemental Fig. S13F–H vs. Supplemental Fig. S9A–H).

To ensure that the observed nanoCAGE biases are general and not specific to our library, we analyzed and compared nAnT-iCAGE and nanoCAGE XL data recently produced on a human K562 cell line (data from Adiconis et al. 2018). Our results were recapitulated with Adiconis et al. data sets (see Supplemental Fig. S14; Supplemental Material).

### Using SLIC-CAGE to uncover promoter architecture

The dominant CTSS provides a structural reference point for the alignment of promoter sequences and thus facilitates the discovery of promoter-specific sequence features. High-quality data is necessary for the accurate identification of the dominant TSS within a tag cluster or promoter region. Sharp promoters, described by small IQ-widths, are typically defined by a fixed distance from a core promoter motif, such as a TATA-box or TATA-like element at −30 position (Ponjavic et al. 2006) upstream of the TSS, or by a DPE motif at +28 to +32 (Kutach and Kadonaga 2000) in *Drosophila*. Broader vertebrate promoters, featuring multiple CTSS positions, are enriched for GC content, and CpG island overlap and also exhibit precisely positioned +1 nucleosomes (Haberle and Lenhard 2016). Lower-complexity libraries have an increased number of artificially sharp tag clusters (Fig. 2F) due to sparse CTSS identification. Although the identified CTSSs in lower-complexity SLIC-CAGE libraries are canonical, their association of sequence features may be obscured by artificially sharp tag clusters, as they will group with true sharp clusters and dilute the signal derived from sequence features (see analysis with 5-ng sample below). To address this, we investigated the promoter architecture for known promoter features in E14 mouse embryonic stem cells (mESC) using SLIC-CAGE from 5 to 100 ng of total RNA.

**Figure 4.**
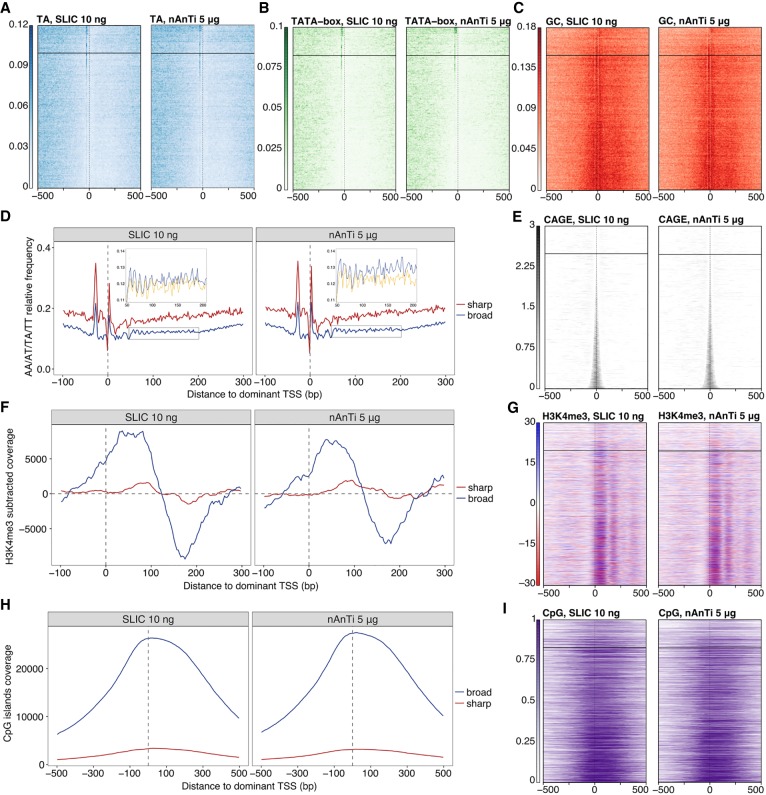
SLIC-CAGE is equivalent to nAnT-iCAGE for pattern discovery. Comparison of SLIC-CAGE derived from 10 ng and nAnT-iCAGE derived from 5 µg of *M. musculus* total RNA. In all heat maps, promoters are centered at the dominant CTSS (dashed vertical line at 0) and ordered by tag cluster inter-quantile width with sharpest promoters on *top* and broadest on the *bottom* of each heat map. The horizontal line separates sharp and broad promoters (empirical boundary for sharp promoters is set at inter-quantile width ≤ 3). (*A*) Comparison of TA dinucleotide density in the SLIC-CAGE (*left*) and nAnT-iCAGE library (*right*). As expected, sharp promoters have a strong TA enrichment, in line with the expected TATA-box in sharp promoters around the −30 position. (*B*) Comparison of TATA-box density in SLIC-CAGE (*left*; 14.3% of TCs have a TATA-box around −30 position) vs. nAnT-iCAGE library (*right*; 14.5% of TCs have a TATA-box around −30 position). Promoter regions are scanned using a minimum of the 80th percentile match to the TATA-box position weight matrix (PWM). Sharp promoters exhibit a strong TATA-box signal as suggested in *A*. (*C*) Comparison of GC dinucleotide density in the SLIC-CAGE (*left*) and nAnT-iCAGE library (*right*). Broad promoters show a higher enrichment of GC dinucleotides across promoters, suggesting the presence of CpG islands, as expected in broad promoters. (*D*) Average WW (AA/AT/TA/TT) dinucleotide frequency in sharp and broad promoters identified in SLIC-CAGE (*left*) or nAnT-iCAGE library (*right*). *Inset* shows a closer view on WW dinucleotide frequency (blue) overlain with the signal obtained when the sequences are aligned to a randomly chosen identified CTSS within broad promoters (yellow). Ten-base-pair WW periodicity implies the presence of well-positioned +1 nucleosomes in broad promoters, in line with the current knowledge on broad promoters. (*E*) Tag cluster coverage heat map of SLIC-CAGE (*left*) or nAnT-iCAGE library (*right*). (*F*) H3K4me3 relative coverage in sharp versus broad promoters identified in SLIC-CAGE (*left*) or nAnT-iCAGE (*right*). Signal enrichment in broad promoters indicates well-positioned +1 nucleosomes, in line with the presence of WW periodicity in broad promoters. (*G*) H3K4me3 signal density across promoter regions centered on SLIC-CAGE or nAnT-iCAGE-identified dominant CTSS. (*H*) Relative coverage of CpG islands across sharp and broad promoters, centered on the dominant CTSS identified in SLIC-CAGE (*left*) or nAnT-iCAGE (*right*). These results agree with GC-dinucleotide density signal, which is much stronger in broad promoters. (*I*) CpG islands coverage signal across promoter regions centered on the dominant CTSS identified in SLIC-CAGE (*left*; 68.1% of TCs overlap with a CpG island) or nAnT-iCAGE (*right*; 64.4% of TCs overlap with a CpG island).

We first assessed the presence of a TA dinucleotide around the −30 positions for all CTSSs identified by SLIC-CAGE for both 5 and 10 ng of input RNA. The dominant CTSSs were ordered by IQ-width of their corresponding tag cluster and extended to include 1 kb DNA sequence up- and downstream (Fig. 4E). The TA frequency is depicted in a heat map in Figure 4A for promoters ordered from sharp to broad for 10 ng of RNA and clearly recapitulates the patterns visible in the reference nAnT-iCAGE library (similarity of heat maps is assessed by permutation testing) (Supplemental Fig. S15A). As expected, the sharpest tag clusters in libraries produced from 5 ng of total RNA have a weaker TA signal (Supplemental Fig. S16A, TA heat map), as these are likely artificially sharp and not the canonical sharp promoters. A similar result is observed for enrichment of the canonical TATA-box element; the 10-ng library recapitulated the reference nAnT-iCAGE library whereas the 5-ng library shows a weaker enrichment (Fig. 4B; Supplemental Figs. S15B, S17C,D).

A GC-enrichment in the region between the dominant TSS and 250 bp downstream from it indicates positioning of the +1 nucleosomes and is expected to be highly localized in broad promoters. This feature is again recapitulated by the 10-ng RNA input library (Fig. 4C; Supplemental Figs. S15C, S16; The FANTOM Consortium et al. 2014; Haberle et al. 2014; Haberle and Lenhard 2016). Furthermore, rotational positioning of the +1 nucleosomes is associated with WW periodicity (AA/AT/TA/TT dinucleotides) lined up with the dominant TSS. We examined WW dinucleotide density separately for sharp and broad promoters identified by SLIC-CAGE and the reference nAnT-iCAGE library (Fig. 4D,E). A strong 10.5-bp periodicity of WW dinucleotides downstream from the dominant TSS was observed in SLIC-CAGE libraries derived from 10 ng of *M. musculus* total RNA and corresponded to the phasing observed with the reference nAnT-iCAGE library (Fig. 4D; Supplemental Fig. S17B). This can only be observed across promoters if the dominant TSS is accurately identified, and therefore it reflects the quality of the libraries. To confirm that WW dinucleotide periodicity reflects +1 nucleosome positioning in broad promoters, we assessed H3K4me3 data downloaded from ENCODE (Fig. 4F,G; Supplemental Fig. S16, H3K4me3 heat map; Supplemental Fig. S15D). H3K4me3-subtracted coverage reflects the well-positioned +1 nucleosome broad promoters (Fig. 4F,G) and localizes with WW periodicity specific for broad promoters (Fig. 4D). These results are in agreement with previously identified nucleosome positioning preferences (Segal et al. 2006).

As a final validation of SLIC-CAGE promoters, we assessed CpG island density separately in sharp and broad promoters (Fig. 4H,I; Supplemental Figs. S15E, S16). We observed a higher density of CpG islands in SLIC-CAGE broad promoters, which corresponds to nAnT-iCAGE broad promoters and agrees with the expected association of broad promoters and CpG islands (Carninci et al. 2006; Haberle and Lenhard 2016). These results demonstrate the utility of SLIC-CAGE libraries derived from nanogram-scale samples in promoter architecture discovery, alongside the gold standard nAnT-iCAGE libraries.

### Uncovering the TSS landscape of mouse primordial germ cells using SLIC-CAGE

Transcriptome, epigenome, and methylome changes occurring during primordial germ cell development have been thoroughly studied (Hajkova 2011; Yamaguchi et al. 2013; Hill et al. 2018). However, the total number of cells and the total amount of RNA obtained per embryo are severely limited. As a result, the underlying regulatory changes at the level of promoter activity and TSS usage have not been addressed, primarily due to a lack of adequate low-input methodology. We applied SLIC-CAGE to mouse primordial germ cells at embryonic day 11.5 (PGC E11.5) using ∼10 ng of total RNA obtained from 5000 to 6000 cells isolated per litter (7–8 embryos) and provide its first promoterome/TSS landscape.

**Figure 5.**
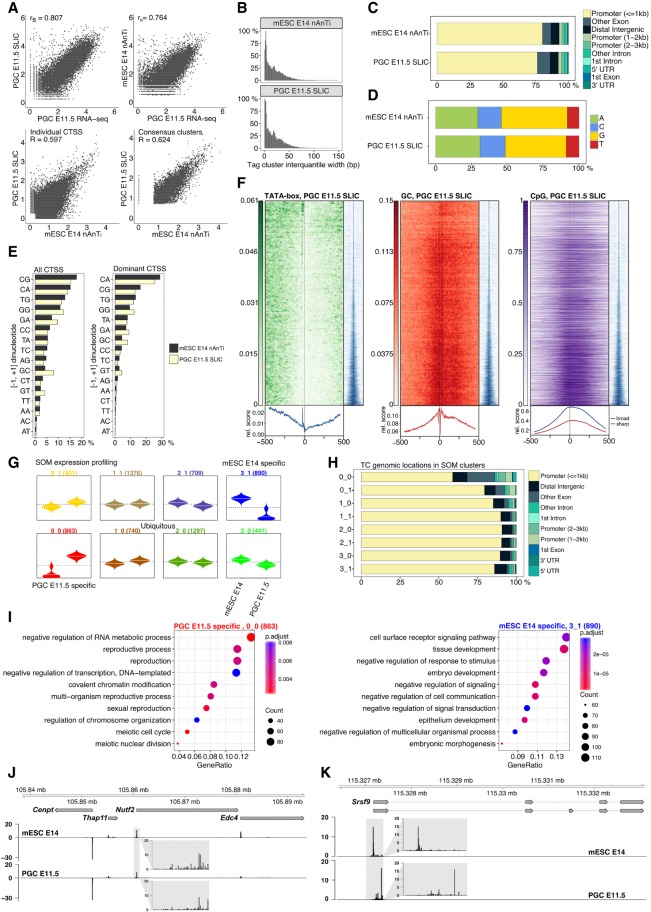
TSS landscape of primordial germ cell E11.5 stage. (*A*) Spearman's correlation of SLIC-CAGE PGC E11.5 data with PGC E11.5 RNA-seq data (*upper left*) or nAnT-iCAGE mESC E14 data with PGC E11.5 RNA-seq data (*upper right* panel). Pearson's correlation of SLIC-CAGE PGC E11.5 and nAnT-iCAGE mESC E14 data sets on individual CTSS level (*lower left*) or consensus tag cluster/promoter level (*lower right* panel). Comparison of nAnT-iCAGE mESC E14 and SLIC-CAGE PGC E11.5 libraries: (*B*) distribution of tag cluster inter-quantile widths; (*C*) genomic locations of tag clusters; (*D*) nucleotide composition of all CTSSs; (*E*) dinucleotide composition of all CTSSs (*left* panel) or dominant CTSSs (*right* panel). Both panels are ordered from the most- to the least-used dinucleotide in the mESC E14 library. (*F*) TATA-box (14.3% of TCs have a TATA-box around −30 position), GC dinucleotide, and CpG island density in PGC E11.5 data (67.3% TCs overlap CpG islands). In all heat maps, promoters are centered at the dominant CTSS (dashed vertical line at 0). Promoter regions are scanned using a minimum of the 80th percentile match to the TATA-box PWM. The signal metaplot is shown *below* each heat map, and a tag cluster IQ-width coverage (in blue) shows ordering in the pattern heat map from sharp to broad tag clusters/promoters (200-bp window centered on dominant TSS). (*G*) Expression profiles obtained by SOM clustering of tag-clusters/promoters. Each box represents one cluster, *left* beanplots represent mESC E14 and *right* beanplots represent PGC E11.5. The horizontal line denotes the mean expression level in each cluster. (*H*) Genomic locations of tag cluster in each SOM class (SOM-classes are shown on the *y*-axis). (*I*) Biological process GO analysis in PGC E11.5-specific SOM class 0_0 (*left*) and mESC E14-specific class 3_1 (*right*). (*J*) CTSS signal in example locus on Chromosome 8 (same as in Fig. 1I) and (*K*) CTSS signal in *Srsf9* promoter region (Chr 5) exhibiting TSS switching in PGC E11.5 compared to mESC E14 (distance between dominant CTSSs is 180 bp). Two transcript variants are shown where thin lines depict introns. The *inset* gray boxes show magnification of tag clusters.

To validate the PGC E11.5 SLIC-CAGE library, we compared CAGE-derived gene expression levels to a published PGC E11.5 RNA-seq data set (Yamaguchi et al. 2013) and found high correlation (Spearman's correlation coefficient 0.81) (Fig. 5A, upper-left panel). The correlation between E11.5 PGC SLIC-CAGE and E11.5 PGC RNA-seq expression is significantly and reproducibly higher than between E11.5 PGC SLIC-CAGE and E14 mESC RNA-seq expression, although mESC E14 and PGC E11.5 have similar TSS/promoter landscapes (Fig. 5A, lower-left and lower-right panels). High similarities of IQ-width distributions (Fig. 5B), genomic locations of identified tag clusters (Fig. 5C), CTSS and dominant CTSS dinucleotide distributions (Fig. 5D,E) between SLIC-CAGE PGC E11.5 and nAnT-iCAGE mESC E14 libraries validate the high quality of the PGC E11.5 SLIC-CAGE data set. Furthermore, we observed canonical promoter types such as sharp and broad promoters used in the PGC E11.5 stage. We also detected classification-associated sequence characteristics, as sharp tag clusters/promoters were associated with the presence of a TATA-box (14.3% of TCs), while broad tag clusters/promoters overlapped with CpG islands (67% of TCs) (Fig. 5F).

Similar results were obtained with an independent biological replicate of PGC E11.5, for which we used ∼10–15 ng of highly degraded total RNA (Supplemental Fig. S18A). Somewhat lower correlation of replicates and lower mapping to promoter regions is observed due to use of degraded RNA; however, the data show no bias in CTSS composition and allow capturing of canonical promoter features (Supplemental Fig. S18B–G). As the data have higher background noise, higher filtering thresholds may need to be applied (for details, see Supplemental Material; Supplemental Fig. S24). This proves that the SLIC-CAGE protocol can be used to generate unbiased TSS landscapes even from degraded samples, which is more often required when the samples are hard to obtain.

We also used the paired-end information on random reverse priming to collapse PCR duplicates in the replicate data—47% of uniquely mapped reads are kept upon de-duplication. We show that de-duplicated data highly correlate with the data prior to de-duplication on both CTSSs and tag cluster levels, causing no changes in IQ-width distribution (Supplemental Fig. S19).

Using self-organizing maps (SOM) expression profiling, we identified differentially and ubiquitously expressed tag clusters in PGC E11.5 stage vs. the mESC E14 cell line (Fig. 5G,H). Biological process GO analysis of the PGC E11.5-specific SOM cluster 0_0 revealed enrichment of reproduction and meiosis-related terms (Fig. 5I, left panel), while mESC E14-specific SOM cluster 3_1 showed tissue and embryo development terms (Fig. 5I, right panel). In line with the recently discovered set of genes crucial for normal gametogenesis (45 germline reprogramming-responsive genes or GRRs) (Hill et al. 2018), we found nine GRR genes in the PGC E11.5-specific 0_0 SOM class (Rad51c, Dazl, Slc25a31, Hormad1, 1700018B24Rik, Fkbp6, Stk31, Asz1, and Taf9b), three GRR genes in the PGC E11.5-specific 0_1 SOM class (Mael, Sycp, and Pnldc), and two in the ubiquitously expressed class 2_0 (D1Pas1 and Hsf2bp). Classes specific to mESC E14 cells or other ubiquitous classes did not contain any GRR genes.

While the TSS landscapes are highly similar (Fig. 5A, lower-left panel; Fig. 5J), we identified several genes with differential TSS usage within the same promoter region (Fig. 5K, right panel, seven switching events with median 29-bp distance between dominant TSSs; Supplemental Fig. S25). This may reflect differential preinitiation machinery, known to differ in gametogenesis (Goodrich and Tjian 2010) but also may cause alternative transcript/protein isoforms, change translational efficiency due to 5′ UTR variation, or alter differential transcript stability (Tamarkin-Ben-Harush et al. 2017; Leppek et al. 2018).

## Discussion

We have developed SLIC-CAGE, an unbiased cap-trapper-based CAGE protocol optimized for promoterome discovery from as little as 5–10 ng of isolated total RNA (∼10^3^ cells, RIN ≥7, as generally recommended for CAGE techniques [Takahashi et al. 2012]). SLIC-CAGE may also be used on low-quality RNA; however, an increase in the amount of starting material may be required for high-quality libraries. We show that SLIC-CAGE libraries are of equivalent quality and complexity as nAnT-iCAGE libraries derived from 500- to 1000-fold more material (5 µg of total RNA, ∼10^6^ cells). SLIC-CAGE extends the nAnT-iCAGE protocol through addition of the degradable carrier to the target RNA material of limited availability. Since the best CAGE protocol is not amenable to downscaling, the idea behind the carrier is to increase the amount of material to permit highly specific cap-trapper-based purification of target RNA polymerase II transcripts and to minimize material loss in many protocol steps.

We designed the carrier to have a similar size distribution and fraction of capped molecules as the total cellular RNA, to effectively saturate nonspecific adsorption sites on all surfaces and matrices used throughout the protocol. In the final stage of SLIC-CAGE, the carrier molecules are selectively degraded using homing endonucleases, while the intact target library is amplified and sequenced. SLIC-CAGE, equally as nAnT-iCAGE (Murata et al. 2014), permits for paired-end sequencing and linking TSSs to transcript architecture. In addition, paired-end data contains information on random priming in reverse transcription, which may be used as a UMI to collapse identical read pairs as PCR duplicates.

We have shown that SLIC-CAGE is superior in sensitivity, resolution, and absence of bias to the only other low-input CAGE technology, nanoCAGE, which relies on template switching during the cDNA synthesis (Plessy et al. 2010). Although the amount of starting material is significantly reduced, the lowest input limit for nanoCAGE is 50 ng of total RNA, which may require up to 30 PCR cycles (Poulain et al. 2017). We directly compared performances of SLIC-CAGE and nanoCAGE in titration tests and demonstrated that (1) higher complexity libraries are achieved with significantly lower input—SLIC-CAGE requires 5–10 ng, while nanoCAGE requires 50 ng of total RNA, (2) nanoCAGE does not recapitulate the complexity of the nAnT-iCAGE libraries even with the highest recommended amount of RNA (500 ng); in comparison SLIC-CAGE captures the full complexity when 5–10 ng are used, (3) nanoCAGE preferentially captures G-starting capped mRNAs, while SLIC-CAGE does not have 5′ mRNA nucleotide dependent biases, and (4) biases in nanoCAGE libraries are independent of the total RNA amount used and inherent to the template switching step. Our results are in agreement with a recent study that demonstrated low performance of nanoCAGE and other template-switching technologies in capturing true transcription initiation events, compared to cap-trapper-based CAGE technologies (Adiconis et al. 2018). However, the effects of template-switching bias are presumably reduced when used for RNA-seq purposes and gene expression profiling (see Supplemental Fig. S23). Overall, cap-trapper-based CAGE methods outperform RAMPAGE, STRT, NanoCAGE-XL, and Oligo-capping (Adiconis et al. 2018).

Importantly, with the carrier approach to minimize the target sample loss, SLIC-CAGE protocol requires fewer PCR amplification cycles—15–18 cycles for 10–1 ng of total RNA as input. This is advantageous as a smaller number of PCR cycles avoids amplification biases and the fraction of observed duplicate reads. Although nanoCAGE takes advantage of unique molecular identifiers to remove PCR duplicates, in our experience, synthesis of truly random UMIs is problematic and subject to variability, thereby obscuring its use.

A different carrier-based approach has recently been applied to downscale chromatin-precipitation-based methods—favored amplification recovery via protection ChIP-seq (FARP-ChIP-seq) (Zheng et al. 2015). FARP-ChIP-seq relies on a designed biotinylated synthetic DNA carrier, mixed with chromatin of interest prior to ChIP-seq library preparation. Amplification of the synthetic DNA carrier is prevented using specific blocker oligonucleotides. This blocker strategy can achieve a 99% reduction in amplification of the biotin-DNA which, if applied instead of our degradable carrier, would leave much more carrier to sequence (starting SLIC-CAGE with 1 ng of total RNA and 5 µg of the carrier, 27% of the carrier is left in the final library, which is more than a 10,000-fold reduction) (Supplemental Table S11).

Finally, we show that SLIC-CAGE is applicable to low-cell-number samples by obtaining the TSS landscape of mouse PGC E11.5 stage. In comparison with mESC E14, we show that PGC E11.5 has highly similar features and canonical promoter signatures. We also identify genes specific to PGC E11.5 stage, further validating the high quality of the PGC E11.5 TSS atlas. Although the TSS landscape in shared promoter regions is similar between mESC E14 and PGC E11.5 stage, we uncover TSS switching events. Identification of TSS switching events and biological follow-up studies will lead to a higher understanding of its functional consequences.

We anticipate that SLIC-CAGE will prove to be invaluable for in-depth and high-resolution promoter analysis of rare cell types, including early embryonic developmental stages or embryonic tissue from a wide range of model organisms, which has so far been inaccessible to the method. With its low material requirement (5–10 ng of total RNA), SLIC-CAGE can also be applied on isolated nascent RNA to provide an unbiased promoterome with high positional and temporal resolution. Lastly, as bidirectional capped RNA is a signature feature of active enhancers (Andersson et al. 2014), deeply sequenced SLIC-CAGE libraries can be used to identify active enhancers in rare cell types. The principle of the degradable carrier can also be easily extended to other protocols where the required amount of RNA or DNA is limiting.

## Methods

### Preparation of the carrier RNA molecules

DNA template (1kb length) for preparation of the carrier by in vitro transcription was synthesized and cloned into a pJ241 plasmid (service by DNA 2.0) (Supplemental Fig. S1; Supplemental Table S1) to produce the carrier plasmid. The template encompasses the gene that serves as the carrier, embedded with restriction sites for I-SceI and I-CeuI to allow degradation in the final steps of the library preparation. The templates for in vitro transcription were prepared by PCR amplification using the unique forward primer (PCR_GN5_f1) (Supplemental Table S2) which introduces the T7 promoter followed by five random nucleotides and the reverse primer which determines the total length of the carrier template and introduces six random nucleotides at the 3′ end (PCR_N6_r1-r10 ) (Supplemental Table S2).

The PCR reaction to produce the carrier templates was composed of 0.2 ng µL^−1^ carrier plasmid, 1 µM primers (each), 0.02 U µL^−1^ Phusion High-Fidelity DNA Polymerase (Thermo Fisher Scientific), and 0.2 mM dNTP in 1× Phusion HF Buffer (final concentrations). The cycling conditions are presented in Supplemental Table S3. Produced carrier templates (lengths 1034–386 bp) were gel-purified to remove nonspecific products.

Carrier RNA was in vitro-transcribed using a HiScribe T7 High Yield RNA Synthesis kit (NEB) according to the manufacturer's instructions and purified using an RNeasy Mini kit (Qiagen). A portion of carrier RNAs was capped using the Vaccinia Capping System and purified using an RNeasy Mini kit (Qiagen). The capping efficiency was estimated using RNA 5′ Polyphosphatase and Terminator 5′-Phosphate-Dependent Exonuclease, as only uncapped RNAs are de-phosphorylated and degraded, while capped RNAs are protected.

Several carrier combinations were tested in SLIC-CAGE (Supplemental Tables S4, S5), and the final carrier used in SLIC-CAGE was comprised of 90% uncapped carrier and 10% capped carrier, both of varying length (Supplemental Table S5). We note that the carrier RNA mix can be prepared within 2 d in large quantities for multiple library preparations and frozen at −80°C until use.

### Sample collection and nucleic acid extraction

*S. cerevisiae* and mouse embryonic stem cells (E14) were grown in standard conditions and total RNA extracted using standard procedures as described in detail in Supplemental Methods.

### SLIC-CAGE library preparation

For the standard cap analysis of gene expression, the latest nAnT-iCAGE protocol was followed (Murata et al. 2014). In the SLIC-CAGE variant, the carrier was mixed with the RNA of interest to the total amount of 5 µg; e.g., 10 ng of RNA of interest were mixed with 4990 ng of carrier mix and subjected to reverse transcription as in the nAnT-iCAGE protocol (Murata et al. 2014). Further library preparation steps were followed as described in Murata et al. (2014) with several exceptions: (1) Samples were pooled only prior to sequencing to allow individual quality control steps; (2) samples were never completely dried using the centrifugal concentrator and then redissolved as in nAnT-iCAGE; instead, the leftover volume was monitored to avoid complete drying and adjusted with water to achieve the required volume; and (3) after the final AMPure purification in the nAnT-iCAGE protocol, each sample was concentrated using the centrifugal concentrator and its volume adjusted to 15 µL, out of which 1 µL was used for quality control on the Agilent Bioanalyzer HS DNA chip.

Steps regarding degradation of the carrier in SLIC-CAGE libraries are schematically presented in Supplemental Figure S20.

To degrade the carrier, 14 µL of sample were mixed with I-SceI (5 U) and I-CeuI (5 U) in 1× CutSmart buffer (NEB) and incubated at 37°C for 3 h. The enzymes were heat-inactivated at 65°C for 20 min and the samples purified using AMPure XP beads (1.8× AMPure XP volume per reaction volume, as described in Murata et al. [2014]). The libraries were eluted in 42 µL of water and concentrated to 20 µL using the centrifugal concentrator.

A qPCR control was then performed to determine the suitable number of PCR cycles for library amplification and assess the amount of the leftover carrier. The primers designed to amplify the whole library are complementary to 5′ and 3′ linker regions, while the primers used to selectively amplify just the carrier are complementary to the 5′ end of the carrier (common to all carrier molecules) and the 3′ linker (common to all molecules in the library) (see Supplemental Table S6 for primer sequences). qPCR reactions were performed using a KAPA SYBR FAST qPCR kit using 1 µL of the sample and 0.1 µM primers (final concentration) in 10 µL total volume using PCR cycle conditions presented in the Supplemental Table S7.

The number of cycles for PCR amplification of the library corresponded to the *C*_*t*_ value obtained with the primers that amplify the whole library (adapter_f1 and adapter_r1) (Supplemental Table S6). PCR amplification of the library was then performed using KAPA HiFi HS ReadyMix, with 0.1 µM primers (adapter_f1 and adapter_r1) (Supplemental Table S6) and 18 µL of sample in a total volume of 100 µL. The cycling program is presented in Supplemental Table S8 and the final number of cycles used to amplify the libraries in Supplemental Table S9. Amplified samples were purified using AMPure XP beads (1.8× volume ratio of the beads to the sample), eluted with 42 µL of water and concentrated using a centrifugal concentrator to 14 µL.

A second round of carrier degradation was then performed as described for the first round. The samples were purified using AMPure XP beads (stringent 1:1 AMPure XP to sample volume ratio to exclude primer dimers and short fragments), eluted with 42 µL of water and concentrated to 12 µL using a centrifugal concentrator. The combination of the first round of carrier degradation followed by PCR amplification, AMPure XP purification, and the second round of carrier degradation is necessary to avoid substantial sample loss that leads to low-complexity libraries.

Each sample was then individually assessed for fragment size distribution using an HS DNA chip (Bioanalyzer, Agilent). If short fragments were present in the library (<300 bp) (see Supplemental Fig. S21), another round of size selection was performed using a stringent volume ratio of AMPure XP beads to the sample of 0.8 X (volume of each sample was, prior to purification, adjusted with water to 30 µL). The samples were eluted in 42 µL of water and concentrated to 12 µL using a centrifugal concentrator. Fragment size distribution was again checked using an HS DNA chip (Bioanalyzer, Agilent) to ensure removal of the short fragments.

Finally, the amount of leftover carrier was estimated using qPCR as described above after the first round of carrier degradation. The expected C_t_ in qPCR using adapter_f1 and adapter_r1 is 12–13 or 23–30 using carrier_f1 and adapter_r1 primer pairs (Supplemental Table S6) when the starting total RNA amount is 100–1 ng.

The libraries were sequenced on MiSeq (*S. cerevisiae*) or HiSeq 2500 (*M. musculus*) Illumina platforms in single-end, 50-bp mode (Genomics Facility, MRC, LMS).

### NanoCAGE library preparation

*S. cerevisiae* nanoCAGE libraries were prepared as described in the latest protocol version by Poulain et al. (2017). Briefly, 5, 10, 25, 50, or 500 ng of *S. cerevisiae* total RNA was reversely transcribed in the presence of corresponding template switching oligonucleotides (Supplemental Table S15), followed by AMPure purification. One 500-ng replicate was pretreated with exonuclease to test if rRNA removal has any effect on the quality of the final library.

The number of PCR cycles for semisuppressive PCR was determined by qPCR as described in Poulain et al. (2017) (Supplemental Table S9). Samples were AMPure purified after amplification and the concentration of each sample determined using Picogreen.

Two nanograms of each sample were pooled prior to tagmentation, and 0.5 ng of the pool was used in tagmentation. The sample was AMPure-purified and quantified using Picogreen prior to MiSeq sequencing in single-end, 50-bp mode (Genomics Facility, MRC, LMS).

### PGC E11.5 isolation and SLIC-CAGE library preparation

E11.5 PGCs were isolated from embryos obtained from a 129Sv female and GOF18ΔPE-EGFP (Yoshimizu et al. 1999) male cross. Briefly, genital ridges from one litter (7–8 embryos) were dissected out and digested at 37°C for 3 min using TrypLE Express (Thermo Fisher Scientific). Enzymatic digestion was neutralized with DMEM/F-12 (Gibco) supplemented with 15% fetal bovine serum (Gibco), followed by manual dissociation by pipetting. The cells were spun down by centrifugation and resuspended in 0.1% BSA PBS. GFP-positive cells were isolated using an Aria Fusion (BD Bioscience) flow cytometer and sorted into ice-cold PBS. Total RNA was isolated from ∼5000–6000 E11.5 PGCs per litter using a DNA/RNA Duet Kit miniprep kit (Zymo Research). The SLIC-CAGE library was then prepared by mixing the obtained ∼10 ng of PGC E11.5 total RNA (measured by Agilent 2100 Bioanalyzer) with 5 µg of the carrier mix and processed as described in SLIC-CAGE library preparation section.

### Processing of CAGE tags: nAnT-iCAGE, SLIC-CAGE, and nanoCAGE

Sequenced CAGE tags (50 bp) were mapped to a reference *S. cerevisiae* genome (sacCer3 assembly) or *M. musculus* genome (mm10 assembly) using Bowtie 2 (Langmead and Salzberg 2012) with default parameters that allow zero mismatches per seed sequence (default 22 nucleotides). Sequenced nanoCAGE libraries were trimmed prior to mapping to remove the linker and UMI region (15 bp from the 5′ end were trimmed). FASTQ files from Adiconis et al. (2018) K562 nanoCAGE XL and nAnT-iCAGE libraries (replicate 1) were obtained from the SRA database (SRR6006247 and SRR6006235). As all nAnT-iCAGE libraries produced in that study were highly correlated, we chose to use only one replicate to match nanoCAGE XL (only one replicate was produced for nanoCAGE XL). Only read1 was used from both libraries and mapped using Bowtie 2 (as described above) to hg19 to match the analysis pipeline from Adiconis et al. (2018). As nanoCAGE and nAnT-iCAGE samples were processed equally and highly mappable (>80%), mapping to hg38 would not influence results.

Only uniquely mapped reads were used in downstream analysis within the R graphical and statistical computing environment (R Core Team 2017) using Bioconductor packages (http://www.bioconductor.org/) and custom scripts. The mapped reads were sorted and imported into R as BAM files using *CAGEr* (Haberle et al. 2015). The additional G nucleotide at the 5′ end of the reads, if added through template-free activity of the reverse transcriptase, was resolved within *CAGEr*’s standard workflow designed to remove Gs that do not map to the genome: (1) If the first nucleotide is G and a mismatch, i.e., it does not map to the genome, it is removed from the read; or (2) if the first nucleotide is G and it matches, it is retained or removed according to the percentage of mismatched G.

Sample replicates and reads from different lanes were merged prior to the final analysis as presented in Supplemental Material (Supplemental Tables S16–S18). All unique 5′ ends represent CAGE tag-supported TSSs, and the number of tags within each CTSS represents expression levels. Raw tag counts were normalized using a referent power-law distribution to a total of 10^6^ tags, resulting in normalized tags per million (TPMs) (Balwierz et al. 2009).

De-duplication of mouse PGC E11.5 replicate 2 was done using Clumpify from the BBMap suite. Information from paired-end reads was utilized to collapse PCR duplicates. Since a random TCTN_6_ primer is used in reverse transcription, identical read pairs are expected to originate from PCR duplicates.

### Data analysis

Analyses were performed using R statistical computing environment (R Core Team 2017) and Bioconductor (Gentleman et al. 2004) packages (http://www.bioconductor.org/). Analyses details are presented in Supplemental Methods.

## Data access

All data generated in this study—nAnT-iCAGE, SLIC-CAGE, and nanoCAGE libraries—have been submitted to ArrayExpress (https://www.ebi.ac.uk/arrayexpress/) under accession numbers E-MTAB-6519 and E-MTAB-7056 (mouse PGC E11.5 data). Custom analysis scripts are available as Supplemental Code and at https://github.com/ncvetesic/SLIC-CAGE.

## Competing interest statement

A patent has been filed for SLIC-CAGE technology.

## Supplementary Material

Supplemental Material
