# Supplementary material for: SLIC-CAGE: high-resolution transcription start site mapping using nanogram-levels of total RNA
Source: Genome Res. 2018 Dec;28(12):1943–56. doi: 10.1101/gr.235937.118 (PMC6280763; doi:10.1101/gr.235937.118)
Supplement: Supplemental Material [file supp_28_12_1943__index.html]

SLIC-CAGE: high-resolution transcription start site mapping using nanogram-levels of total RNA — SLIC-CAGE: high-resolution transcription start site mapping using nanogram-levels of total RNA — Supplemental Material 

# SLIC-CAGE: high-resolution transcription start site mapping using nanogram-levels of total RNA

## Supplemental Material

- Supplemental\_code.zip
- Supplemental\_Material.pdf
