# Supplementary material for: SLIC-CAGE: high-resolution transcription start site mapping using nanogram-levels of total RNA
Source: Genome Res. 2018 Dec;28(12):1943–56. doi: 10.1101/gr.235937.118 (PMC6280763; doi:10.1101/gr.235937.118)
Supplement: Supplemental Material [file supp_gr.235937.118_Supplemental_code.zip › Supplemental_code/README.html]

# SLIC-CAGE: high-resolution transcription start site mapping using nanogram-levels of total RNA

## Abstract

Cap analysis of gene expression (CAGE) is a methodology for genome-wide quantitative mapping of mRNA 5’ends to precisely capture transcription start sites at a single nucleotide resolution. In combination with high-throughput sequencing, CAGE has revolutionized our understanding of rules of transcription initiation, led to discovery of new core promoter sequence features and discovered transcription initiation at enhancers genome-wide. The biggest limitation of CAGE is that even the most recently improved version (nAnT-iCAGE) still requires large amounts of total cellular RNA (5 micrograms), preventing its application to scarce biological samples such as those from early embryonic development or rare cell types. Here, we present SLIC-CAGE, a Super-Low Input Carrier-CAGE approach to capture 5’ends of RNA polymerase II transcripts from as little as 5-10 ng of total RNA. The dramatic increase in sensitivity is achieved by specially designed, selectively degradable carrier RNA. We demonstrate the ability of SLIC-CAGE to generate data for genome-wide promoterome with 1000-fold less material than required by existing CAGE methods by generating a complex, high quality library from mouse embryonic day (E) 11.5 primordial germ cells.

## Requirements

- Install following R packages from CRAN: dplyr, tidyr, ggplot2, RColorBrewer, viridis, reshape2, cowplot, corrplot, zoo, philentropy
- Install following R packages from bioconductor: CAGEr, SeqPattern, heatmaps, GenomicRanges, BSgenome.Scerevisiae.UCSC.sacCer3, BSgenome.Mmusculus.UCSC.mm10, TxDb.Mmusculus.UCSC.mm10.knownGene, GenomicFeatures, org.Mm.eg.db, ChIPseeker, TxDb.Scerevisiae.UCSC.sacCer3.sgdGene, biomaRt, Gviz, DESeq2

## Figure to code map

- Figure 1 B-D and F-H, E and I
- Figure 2 A, B and F, C and G, D and H
- Figure 3 A-F, G, H, I, J, K
- Figure 4 A-C, D, E, F-G, H-I
- Figure 5 A, B, C, D, E, F, G, H, I, J-K
- Supplemental Figure S1 C-I, J, K, L, M
- Supplemental Figure S2 A-C, D-F, G-I, J-O
- Supplemental Figure S3
- Supplemental Figure S4 A-C
- Supplemental Figure S5 A-C
- Supplemental Figure S6-S8
- Supplemental Figure S9-S10, S13
- Supplemental Figure S11
- Supplemental Figure S12
- Supplemental Figure S14 A-B, C, D, E, F, G
- Supplemental Figure S15
- Supplemental Figure S16 A-F

## Additional information

- Pre-processing pipelines are available upon request.
- Processed data (*.bam,* .bw, CTSS or tag cluster tables, etc.) and intermediate data is available upon request.

## Reference

**SLIC-CAGE: high-resolution transcription start site mapping using nanogram-levels of total RNA**

biorxiv preprint
