# Supplementary material for: SLIC-CAGE: high-resolution transcription start site mapping using nanogram-levels of total RNA
Source: Genome Res. 2018 Dec;28(12):1943–56. doi: 10.1101/gr.235937.118 (PMC6280763; doi:10.1101/gr.235937.118)
Supplement: Supplemental Material [file supp_gr.235937.118_Supplemental_Material.pdf]

Supplemental Material for

SLIC-CAGE: high-resolution transcription start site mapping using nanogram-levels of total RNA

Nevena Cvetesic[1, 2*], Harry G. Leitch[1,2], Malgorzata Borkowska[1,2], Ferenc Müller[3], Piero Carninci[4,5], Petra Hajkova[1,2], Boris Lenhard[1,2,6*]

[1]Institute of Clinical Sciences, Faculty of Medicine, Imperial College London, London W12 0NN, UK
[2]MRC London Institute of Medical Sciences, London W12 0NN, UK
[3]Institute of Cancer and Genomic Sciences, University of Birmingham, Edgbaston B15 2TT, UK
[4]RIKEN Center for Life Science Technologies, Division of Genomic Technologies, Yokohama City, Kanagawa 230-0045, Japan
[5]RIKEN Omics Science Center, Yokohama City, Kanagawa 230-0045, Japan
[6]Sars International Centre for Marine Molecular Biology, University of Bergen, N-5008 Bergen, Norway

*Correspondence to:

Nevena Cvetesic, Ph.D.
Institute of Clinical Sciences,
Faculty of Medicine,
Imperial College London and
MRC London Institute of Medical Sciences,
London, W12 0NN, UK
e-mail: ncvetesi@ic.ac.uk

Prof. Boris Lenhard, Ph.D.
Institute of Clinical Sciences,
Faculty of Medicine,
Imperial College London and
MRC London Institute of Medical Sciences,
London, W12 0NN, UK
e-mail: b.lenhard@imperial.ac.uk

Running title: TSS discovery using Super-Low Input Carrier-CAGE

TABLE OF CONTENTS

SUPPLEMENTAL TABLES: 3-17

Supplemental Table S1. Sequence of the carrier synthetic gene: **3**
Supplemental Table S2. Primers used to amplify carrier molecules: **4**
Supplemental Table S3. PCR conditions used to create carrier templates: **5**
Supplemental Table S4. Carrier combinations tested in SLIC-CAGE: **6**
Supplemental Table S5. Carrier molecule quantities used in SLIC-CAGE: **7**
Supplemental Table S6. Primer sequences for qPCR used to estimate the ratio of target library and the leftover carrier: **8**
Supplemental Table S7. Real-time qPCR cycling conditions: **9**
Supplemental Table S8. PCR amplification conditions: **10**
Supplemental Table S9. Number of PCR cycles used to amplify SLIC-CAGE and nanoCAGE libraries: **11**
Supplemental Table S10. SLIC-CAGE, nAnT-iCAGE and nanoCAGE mapping efficiency: **12**
Supplemental Table S11. SLIC-CAGE carrier leftover: **13**
Supplemental Table S12. CTSS and tag cluster identification in SLIC-CAGE and nAnTi-CAGE: **14**
Supplemental Table S13. CTSS and tag cluster identification in nanoCAGE: **15**
Supplemental Table S14. Alignment mismatches in nanoCAGE tags: **16**
Supplemental Table S15. CTSS and tag cluster identification in nanoCAGE: **17**
Supplemental Table S16. Additional information on *S. cerevisiae* SLIC-CAGE and nAnT-iCAGE libraries: **18**
Supplemental Table S17. Additional information on *M. musculus* SLIC-CAGE and nAnT-iCAGE libraries: **19**
Supplemental Table S18. Additional information on *S. cerevisiae* nanoCAGE libraries: **20**

SUPPLEMENTAL FIGURES: 21-58

Supplemental Fig. S1. Design and test of carrier molecules: **21-22**
Supplemental Fig. S2. Performance comparison of SLIC-CAGE and nanoCAGE libraries: **23-24**
Supplemental Fig. S3. Distributions of tag cluster interquantile widths in subsampled *S. cerevisiae* nAnT-iCAGE libraries: **25**
Supplemental Fig. S4. Distributions of tag cluster interquantile widths: **26**
Supplemental Fig. S5. ROC curves for samples in SLIC-CAGE and nanoCAGE libraries: **27**
Supplemental Fig. S6. Precision of dominant TSS identification in *S. cerevisiae* SLIC-CAGE libraries: **28**
Supplemental Fig. S7. Precision of dominant TSS identification in *M. musculus* SLIC-CAGE libraries: **29**
Supplemental Fig. S8. Precision of dominant TSS identification in *S. cerevisiae* nanoCAGE libraries: **30**
Supplemental Fig. S9. Assessment of positional accuracy in *S. cerevisiae* SLIC-CAGE libraries prepared from various amounts of total RNA: **31-33**
Supplemental Fig. S10. Assessment of positional accuracy in *M. musculus* SLIC-CAGE libraries prepared from various amounts of total RNA: **34-35**
Supplemental Fig. S11. Dinucleotide composition of dominant CTSSs: **36-37**
Supplemental Fig. S12. Dinucleotide composition of dominant CTSSs: **38**
Supplemental Fig. S13. Assessment of CTSS positional accuracy in nanoCAGE: **39-41**
Supplemental Fig. S14. Comparison K562 cell line CAGE and nanoCAGE XL data: **42-43**
Supplemental Fig. S15. Similarity of patterns discovered in mESC E14 SLIC-CAGE 10 ng sample and mESC E14 nAnT-iCAGE 5 µg sample: **44**
Supplemental Fig. S16. Pattern discovery in *M. musculus* SLIC-CAGE libraries: **45-46**
Supplemental Fig. S17. Separation of sharp and broad promoters/tag clusters in *M. musculus* SLIC-CAGE libraries: **47-48**
Supplemental Fig. S18. Validation of PGC E11.5 SLIC-CAGE libraries: **49-50**
Supplemental Fig. S19. Correlation of SLIC-CAGE libraries prior to and after deduplication: **51**
Supplemental Fig. S20. Detailed workflow of SLIC-CAGE protocol steps following carrier degradation: **52**
Supplemental Fig. S21. Representative SLIC-CAGE HS DNA bioanalyzer traces: **53**
Supplemental Fig. S22. Genome browser screenshots of RPL6B locus: **54-55**
Supplemental Fig. S23. Correlation of nanoCAGE and nAnT-iCAGE libraries on promoter/tag cluster level: **56**
Supplemental Fig. S24. Analyses of PGC E11.5 replicate 2: **57-58**
Supplemental Fig. S25. CTSS signal in regions with TSS switching in PGC E11.5 compared to mESC E14: **59**

SUPPLEMENTAL METHODS: 60-63

SUPPLEMENTAL RESULTS: 64-65

SUPPLEMENTAL REFERENCES: 66

SUPPLEMENTAL TABLES

Supplemental Table S1. Sequence of the carrier synthetic gene.
I-SceI recognition sites are highlighted in red, while I-CeuI recognitions sites are highlighted in blue.

```
5'CAGCGTTCGCTATAACTATAACGGTCCTAAGGTAGCGAAATGCAAGAGCAATACCGCCCGGAAGAGATAGAATCCAA
AGTACAGCTTCATAGGGATAACAGGGTAATTTGGGATGAGAAGCGCACATTTGAAGTAACCGAAGACGAGAGCAAAGAG
ATAACTATAACGGTCCTAAGGTAGCGAAAGTATTACTGCCTGTCTATGCTTCCCTATCCTTCTGGTCGACTACACATGT
AGGGATAACAGGGTAATGGCCACGTACGTAACTACACCATCGGTGACGTGATCGCCCGCTACCAGCGTAACTATAACGG
TCCTAAGGTAGCGAATATGCTGGGCAAAAACGTCCTGCAGCCGATCGGCTGGGACGCGTTTGGTCTAGGGATAACAGGG
TAATTGCCTGCGGAAGGCGCGGCGGTGAAAAACAACACCGCTCCGGCACCGTGGTAACTATAACGGTCCTAAGGTAGCG
AAACGTACGACAACATCGCGTATATGAAAAACCAGCTCAAAATGCTGGGCTTTAGGGATAACAGGGTAATTGGTTATGA
CTGGAGCCGCGAGCTGGCAACCTGTACGCCGGAATACTACCTAACTATAACGGTCCTAAGGTAGCGAAGTTGGGAACAG
AAATTCTTCACCGAGCTGTATAAAAAAGGCCTGGTATATTAGGGATAACAGGGTAATAAGAAGACTTCTGCGGTCAACT
GGTGCCCGAACGACCAGACCGTACTGGCTAACTATAACGGTCCTAAGGTAGCGAAGAACGAACAAGTTATCGACGGCTG
CTGCTGGCGCTGCGATACCAAAGTTGTAGGGATAACAGGGTAATAACGTAAAGAGATCCCGCAGTGGTTTATCAAAATC
ACTGCTTACGCTGACTAACTATAACGGTCCTAAGGTAGCGAATTGCAGCTCAACGATCTGGATAAACTGGATCACTGGC
CAGACACCGTTAATAGGGATAACAGGGTAATCGAATTCGTCTGCGACACGTAG3'
```

I-SceI: TAGGGATAACAGGGTAAT

I-CeuI: TAACTATAACGGTCCTAAGGTAGCGAA

Supplemental Table S2. Primers used to amplify carrier molecules.

carrier nr	reverse primer sequence 5'-3'	PCR product length / bp
1	PCR_N6_r1: NNNNNNCTACGTGTCGCAGACGAATT	1034
2	PCR_N6_r2: NNNNNNTATCCAGATCGTTGAGCTGC	966
3	PCR_N6_r3: NNNNNNCACTGCGGGATCTCTTTACG	889
4	PCR_N6_r4: NNNNNNGCCGTCGATAACTTGTTCGT	821
5	PCR_N6_r5: NNNNNNAGTTGACCGCAGAAGTCTTC	744
6	PCR_N6_r6: NNNNNNGTGAAGAATTTCTGTTCCCA	676
7	PCR_N6_r7: NNNNNNCTCGCGGCTCCAGTCATAAC	599
8	PCR_N6_r8: NNNNNNTATACGCGATGTTGTCGTAC	531
9	PCR_N6_r9: NNNNNNACCGCCGCGCCTTCCGCAGG	454
10	PCR_N6_r10: NNNNNNCAGGACGTTTTTGCCCAGCA	386

The same forward primer is used to create PCR templates for all carrier molecules (see below).
T7 promoter sequence is underlined:
PCR_GN5_f1: TAATACGACTCACTATAGNNNNNCAGCGTTCGCTA

Supplemental Table S3. PCR conditions used to create carrier templates.

steps	temperature (°C)	duration	cycles
initial denaturation	98	1 minute	1
denature	98	10 seconds	35
annealing	50	30 seconds	
extension	72	30 seconds	
final extension	72	10 minutes	1

Supplemental Table S4. Carrier combinations tested in SLIC-CAGE.

carrier nr	uncapped / µg	capped / µg
	carrier mix 1	
1	4.4	0.5
	carrier mix 2	
1-10[a]	4.4	0.5
	carrier mix 3	
1	0	0.5
	carrier mix 4	
1-10[a]	0	0.5

[a] Proportions of each carrier used are given in Supplemental Table 5.

Supplemental Table S5. Carrier molecule quantities used in SLIC-CAGE.

carrier nr	uncapped / µg	capped / µg
1	3.96	0.45
2	8.36	0.95
3	4.40	0.50
4	6.60	0.75
5	4.40	0.50
6	3.08	0.35
7	4.40	0.50
8	3.96	0.45
9	2.64	0.30
10	2.20	0.25

Provides approximately 50 µg of the carrier mix 0.3–1 kb (44 µg of uncapped and 5 µg of capped).

Supplemental Table S6. Primer sequences for qPCR used to estimate the ratio of target library and the leftover carrier.

primer	sequence 5'-3'	description
carrier_f1	GCGGCAGCGTTCGCTATAAC	forward primer for all carrier molecules
adapter_f1	AATGATACGGCGACCACCGA	forward primer complementary to 5'adapters
adapter_r1	CAAGCAGAAGACGGCATACGA	reverse primer complementary to 3'adapters

Supplemental Table S7. Real-time qPCR cycling conditions.

steps	temperature (°C)	duration	cycles
initial denaturation	95	30 seconds	1
denature	98	15 seconds	40
annealing	65	10 seconds	
extension	68	2 minutes	
melting curve		instrument specific program	

Supplemental Table S8. PCR amplification conditions.

steps	temperature (°C)	duration	cycles
initial denaturation	95	30 seconds	1
denature	98	15 seconds	X[a]
annealing	65	10 seconds	
extension	68	2 minutes	
final extension	68	2 minutes	1

[a] X corresponds to Ct value obtained in qPCR with adapter_f1 and adapter_r1 primers.

Supplemental Table S9. Number of PCR cycles used to amplify SLIC-CAGE and nanoCAGE libraries.

samples	nr of PCR cycles
Saccharomyces cerevisiae	
SLIC 1 ng	18
SLIC 2 ng	17
SLIC 5 ng	16
SLIC 10 ng r1	15
SLIC 10 ng r2	15
SLIC 25 ng	15
SLIC 50 ng	15
SLIC 100 ng	15
nAnTi 5 µg PCR[a]	13
nAnTi 5 µg	0
nano 5 ng	20
nano 10 ng r1	20
nano 10 ng r2	20
nano 25 ng r1	17
nano 25 ng r2	20
nano 50 ng	17
nano 500 ng r1	15
nano 500 ng r2	15
Mus musculus	
SLIC 5 ng	16
SLIC 10 ng	15
SLIC 25 ng	14
SLIC 50 ng	13
SLIC 100 ng	12
nAnTi 5 µg	0
PGC E11.5 r1	18
PGC E11.5 r2	18

[a] reference nAnT-iCAGE sample diluted 100-fold and PCR amplified 13 cycles using adapter_f1 and adapter_r1 primers.

Supplemental Table S10. SLIC-CAGE, nAnT-iCAGE and nanoCAGE mapping efficiency.

samples	total nr of reads	% overall mapped	% uniquely mapped	% multimappers	% unmapped
		Saccharomyces cerevisiae			
SLIC 1 ng	4402165	32.0	24.6	7.4	68.0
SLIC 2 ng	3253571	62.5	49.9	12.6	37.5
SLIC 5 ng	3151743	75.4	59.2	16.2	24.6
SLIC 10 ng r1	3153689	71.2	59.7	11.5	28.8
SLIC 10 ng r2	3241105	56.3	47.3	9.1	43.7
SLIC 25 ng	2454447	80.4	70.6	9.8	19.6
SLIC 50 ng	3365660	82.8	72.4	10.4	17.2
SLIC 100 ng	2688732	84.7	74.0	10.6	15.3
nAnTi 5 µg PCR	3154255	86.4	75.7	10.7	13.6
nAnTi 5 µg	1456421	85.9	72.3	13.6	14.1
nano 5 ng	1451548	93.1	51.0	42.1	6.9
nano 10 ng r1	1300442	92.9	56.7	36.3	7.1
nano 10 ng r2	1277753	95.3	19.1	76.2	19.1
nano 25 ng r1	970985	92.9	63.0	29.9	7.1
nano 25 ng r2	551169	93.3	18.7	74.6	6.7
nano 50 ng	957838	94.0	58.4	35.6	6.0
nano 500 ng r1	2013089	92.5	75.9	16.6	7.5
nano 500 ng r2	1330674	92.4	76.5	15.9	7.7
		Mus musculus			
SLIC 5 ng	18205750	50.3	30.8	19.5	49.6
SLIC 10 ng	31403275	59.0	37.0	22.0	40.9
SLIC 25 ng	36979965	65.8	42.6	24.2	33.1
SLIC 50 ng	23750223	65.0	39.2	25.8	35.0
SLIC 100 ng	24886015	70.2	43.7	26.5	29.8
nAnTi 5 µg	7806932	82.2	47.0	35.2	17.8

Supplemental Table S11. SLIC-CAGE carrier leftover.

samples	total nr of reads	nr of unmapped reads	nr of reads mapped to the carrier	% of reads mapped to the carrier
		Saccharomyces cerevisiae		
SLIC 1 ng	4402165	2993472	1184220	27
SLIC 2 ng	3253571	1220089	322186	10
SLIC 5 ng	3151743	775329	204155	6.5
SLIC 10 ng r1	3153689	908262	482002	15
SLIC 10 ng r2	3241105	1416363	450066	14
SLIC 25 ng	2454447	481072	93099	3.8
SLIC 50 ng	3365660	578894	86783	2.6
SLIC 100 ng	2688732	411376	29334	1.1
		Mus musculus		
SLIC 5 ng	18205750	9030052	1221861	6.7
SLIC 10 ng	31403275	12843939	652611	2.1
SLIC 25 ng	36979965	12240368	239794	0.7
SLIC 50 ng	23750223	8312578	99080	0.4
SLIC 100 ng	24886015	7416032	47264	0.2

Supplemental Table S12. CTSS and tag cluster identification in SLIC-CAGE and nAnTi-CAGE.

samples	nr of unique CTSS	% CTSS overlap with nAnTi	nr of unique TCs	% TC overlap with nAnTi	% of domCTSS[a] within 10 bp
			Saccharomyces cerevisiae		
SLIC 1 ng	40990	75.5	8066	77.3	66.8
SLIC 2 ng	56018	72.0	7770	84.4	69.3
SLIC 5 ng	83359	71.5	8006	89.4	75.1
SLIC 10 ng r1	85275	70.8	8255	88.3	74.9
SLIC 10 ng r2	93770	69.3	8653	86.3	75.8
SLIC 25 ng	91947	71.4	8398	88.7	75.7
SLIC 50 ng	99453	71.0	8618	88.0	77.3
SLIC 100 ng	95628	72.6	8476	88.5	77.7
nAnTi 5 µg PCR	100123	71.8	8764	87.5	78.8
nAnTi 5 µg	85661	100.0	8095	100.0	100
			Mus musculus		
SLIC 5 ng	103599	74.6	13574	77.8	69.7
SLIC 10 ng	164145	79.3	14598	87.8	76.1
SLIC 25 ng	175361	81.9	15074	89.3	78.6
SLIC 50 ng	172425	83.9	15131	90.4	79.8
SLIC 100 ng	183951	84.7	15811	90.1	82.7
nAnTi 5 µg	177291	100	15918	100.0	100

[a]Percentage of library identified dominant CTSSs within 10 bp distance from nAnT-iCAGE identified dominant CTSS within the same tag cluster.

Supplemental Table S13. CTSS and tag cluster identification in nanoCAGE.

samples	nr of unique CTSS	% CTSS overlap with nAnTi	nr of unique TCs	% TC overlap with nAnTi	% of domCTSS[a] within 10 bp
		Saccharomyces cerevisiae			
nano 5 ng	11750	62.3	4717	71.8	59.1
nano 10 ng r1	19693	58.5	6835	66.1	58.7
nano 10 ng r2	11778	48.5	5346	55.7	59.8
nano 25 ng r1	55207	42.4	8505	72.4	59.6
nano 25 ng r2	23179	40.1	14198	37.3	55.9
nano 50 ng	63061	38.8	9028	70.0	59.1
nano 500 ng r1	78456	46.1	9502	74.0	60.1
nano 500 ng r2	62452	49.9	8385	81.0	59.7
nAnTi 5 µg r1	91497	100.0	9389	100.0	72.4
nAnTi 5 µg r2	90550	50.4	9288	80.1	100

[a]Percentage of library identified dominant CTSSs within 10 bp distance from nAnT-iCAGE identified dominant CTSS within the same tag cluster.

Supplemental Table S14. Alignment mismatches in nanoCAGE tags.

samples	nr of sequences in BAM files	nr of NN mismatches[a]	nr of GG mismatches[b]
nano 5 ng	706721	202	144
nano 10 ng r1	688288	154	104
nano 10 ng r2	237576	162	120
nano 25 ng r1	579426	114	84
nano 25 ng r2	101962	128	93
nano 50 ng	528555	141	105
nano 500 ng r1	1455076	701	483
nano 500 ng r2	988083	386	271

[a]Number of alignment mismatches at the 1st and 2nd nucleotide position in nanoCAGE tags.
[b]Number of GG dinucleotides at 1st and 2nd position in nanoCAGE tags, flagged as mismatches in the alignment.

Supplemental Table S15. Template switching oligonucleotides used in nanoCAGE.

samples	TSO nr[a]	barcode
nano 5 ng	4	ACAGAT
nano 10 ng r1	31	CACGAT
nano 10 ng r2	79	GTATAC
nano 25 ng r1	36	CACTGA
nano 25 ng r2	83	TATAGC
nano 50 ng	46	CTGACG
nano 500 ng r1	63	GAGTGA
nano 500 ng r2	71	GCTGCA

[a]TSO sequences are from Poulain *et al* 2017 (Poulain et al. 2017)

Supplemental Table S16. Additional information on *S. cerevisiae* SLIC-CAGE and nAnT-iCAGE libraries.

SAMPLE NAMES	ORGANISM	PLATFORM	METHOD	TOTAL RNA INPUT / ng	EXPERIMENT INDEX[a]	LANE INDEX[b]	MERGE INDEX[c]
sc_slic_1ng_r1	*S. cerevisiae*	MiSeq	SLIC-CAGE	1	1	1	1
sc_slic_1ng_r2	*S. cerevisiae*	MiSeq	SLIC-CAGE	1	1	1	1
sc_slic_2ng_r1	*S. cerevisiae*	MiSeq	SLIC-CAGE	2	1	1	2
sc_slic_2ng_r2	*S. cerevisiae*	MiSeq	SLIC-CAGE	2	1	1	2
sc_slic_5ng_r1	*S. cerevisiae*	MiSeq	SLIC-CAGE	5	1	1	3
sc_slic_5ng_r2	*S. cerevisiae*	MiSeq	SLIC-CAGE	5	1	1	3
sc_slic_10ng_t1_r1	*S. cerevisiae*	MiSeq	SLIC-CAGE	10	2	2	4
sc_slic_10ng_t1_r2	*S. cerevisiae*	MiSeq	SLIC-CAGE	10	2	2	4
sc_slic_10ng_t2_r1	*S. cerevisiae*	MiSeq	SLIC-CAGE	10	1	1	5
sc_slic_10ng_t2_r2	*S. cerevisiae*	MiSeq	SLIC-CAGE	10	1	1	5
sc_slic_25ng_r1	*S. cerevisiae*	MiSeq	SLIC-CAGE	25	2	2	6
sc_slic_25ng_r2	*S. cerevisiae*	MiSeq	SLIC-CAGE	25	2	2	6
sc_slic_50ng_r1	*S. cerevisiae*	MiSeq	SLIC-CAGE	50	2	2	7
sc_slic_50ng_r2	*S. cerevisiae*	MiSeq	SLIC-CAGE	50	2	2	7
sc_slic_100ng_r1	*S. cerevisiae*	MiSeq	SLIC-CAGE	100	2	2	8
sc_slic_100ng_r2	*S. cerevisiae*	MiSeq	SLIC-CAGE	100	2	2	8
sc_nanti_PCR	*S. cerevisiae*	MiSeq	nAnT-iCAGE	5000	3	3	9
sc_nanti_r1	*S. cerevisiae*	MiSeq	nAnT-iCAGE	5000	4	4	10
sc_nanti_r2	*S. cerevisiae*	MiSeq	nAnT-iCAGE	5000	4	4	10

[a] indicates which samples were prepared at the exact same time (in parallel). Same index denotes the same time of the experiment.
[b] indicates if the samples were sequenced on the same lane in the same run
[c] indicates which reads/samples were merged prior to analysis in R (merge option of samples within CAGEr)

Supplemental Table S17. Additional information on *M. musculus* SLIC-CAGE and nAnT-iCAGE libraries.

SAMPLE NAMES[a]	ORGANISM	PLATFORM	METHOD	TOTAL RNA INPUT / ng	EXPERIMENT INDEX[b]	LANE INDEX[c]	MERGE INDEX[d]
E14_5ng_L3	*M. musculus*	HiSeq2500	SLIC-CAGE	5	1	1	1
E14_5ng_L4	*M. musculus*	HiSeq2500	SLIC-CAGE	5	1	2	1
E14_10ng_r1_L3	*M. musculus*	HiSeq2500	SLIC-CAGE	10	1	1	2
E14_10ng_r1_L4	*M. musculus*	HiSeq2500	SLIC-CAGE	10	1	2	2
E14_10ng_r2_L3	*M. musculus*	HiSeq2500	SLIC-CAGE	10	1	1	2
E14_10ng_r2_L4	*M. musculus*	HiSeq2500	SLIC-CAGE	10	1	2	2
E14_25ng_r1_L3	*M. musculus*	HiSeq2500	SLIC-CAGE	25	1	1	3
E14_25ng_r1_L4	*M. musculus*	HiSeq2500	SLIC-CAGE	25	1	2	3
E14_25ng_r2_L3	*M. musculus*	HiSeq2500	SLIC-CAGE	25	1	1	3
E14_25ng_r2_L4	*M. musculus*	HiSeq2500	SLIC-CAGE	25	1	2	3
E14_50ng_L3	*M. musculus*	HiSeq2500	SLIC-CAGE	50	1	1	4
E14_50ng_L4	*M. musculus*	HiSeq2500	SLIC-CAGE	50	1	2	4
E14_100ng_L3	*M. musculus*	HiSeq2500	SLIC-CAGE	100	1	1	5
E14_100ng_L4	*M. musculus*	HiSeq2500	SLIC-CAGE	100	1	2	5
E14_nanti	*M. musculus*	HiSeq2500	nAnT-iCAGE	5000	2	3	6
PGC_E11_5_L1	*M. musculus*	HiSeq2500	SLIC-CAGE	10	3	4	7
PGC_E11_5_L2	*M. musculus*	HiSeq2500	SLIC-CAGE	10	3	4	7
PGC_E11_5_L3	*M. musculus*	HiSeq2500	SLIC-CAGE	10	3	4	7
PGC_E11_5_L4	*M. musculus*	HiSeq2500	SLIC-CAGE	10	3	4	7
PGC_E11_5_r2_R1	*M. musculus*	HiSeq2500	SLIC-CAGE	10	4	4	-
PGC_E11_5_r2_R2	*M. musculus*	HiSeq2500	SLIC-CAGE	10	4	4	-

[a] samples with L in the name denote the same library sequenced on multiple lanes, r in the name stands for replicate, samples with R1 and R2 denote the same library – sequenced in paired end mode, Read1 and Read2

[b] indicates which samples were prepared at the exact same time (in parallel). Same index denotes the same time of the experiment.

[c] indicates if the samples were sequenced on the same lane in the same run

[d] indicates which reads/samples were merged prior to analysis in R (merge option of samples within CAGEr)

Supplemental Table S18. Additional information on *S. cerevisiae* nanoCAGE libraries.

SAMPLE NAMES	ORGANISM	PLATFORM	METHOD	TOTAL RNA INPUT / ng	EXPERIMENT INDEX[a]	LANE INDEX[b]	MERGE INDEX[c]
sc_nano_5ng	*S. cerevisiae*	MiSeq	nanoCAGE	5	1	1	1
sc_nano_10ng_r1	*S. cerevisiae*	MiSeq	nanoCAGE	10	1	1	2
sc_nano_10ng_r2	*S. cerevisiae*	MiSeq	nanoCAGE	10	1	1	3
sc_nano_25ng_r1	*S. cerevisiae*	MiSeq	nanoCAGE	25	1	1	4
sc_nano_25ng_r2	*S. cerevisiae*	MiSeq	nanoCAGE	25	1	1	5
sc_nano_50ng_r1	*S. cerevisiae*	MiSeq	nanoCAGE	50	1	1	6
sc_nano_500ng_r1	*S. cerevisiae*	MiSeq	nanoCAGE	500	1	1	7
sc_nano_500ng_r2	*S. cerevisiae*	MiSeq	nanoCAGE	500	1	1	8
sc_nanti_r1	*S. cerevisiae*	MiSeq	nAnT-iCAGE	5000	2	2	9
sc_nanti_r2	*S. cerevisiae*	MiSeq	nAnT-iCAGE	5000	2	2	10

[a] indicates which samples were prepared at the exact same time (in parallel). Same index denotes the same time of the experiment.
[b] indicates if the samples were sequenced on the same lane in the same run
[c] indicates which reads/samples were merged prior to analysis in R (merge option of samples within CAGEr)

SUPPLEMENTAL FIGURES

Supplemental Figure S1.

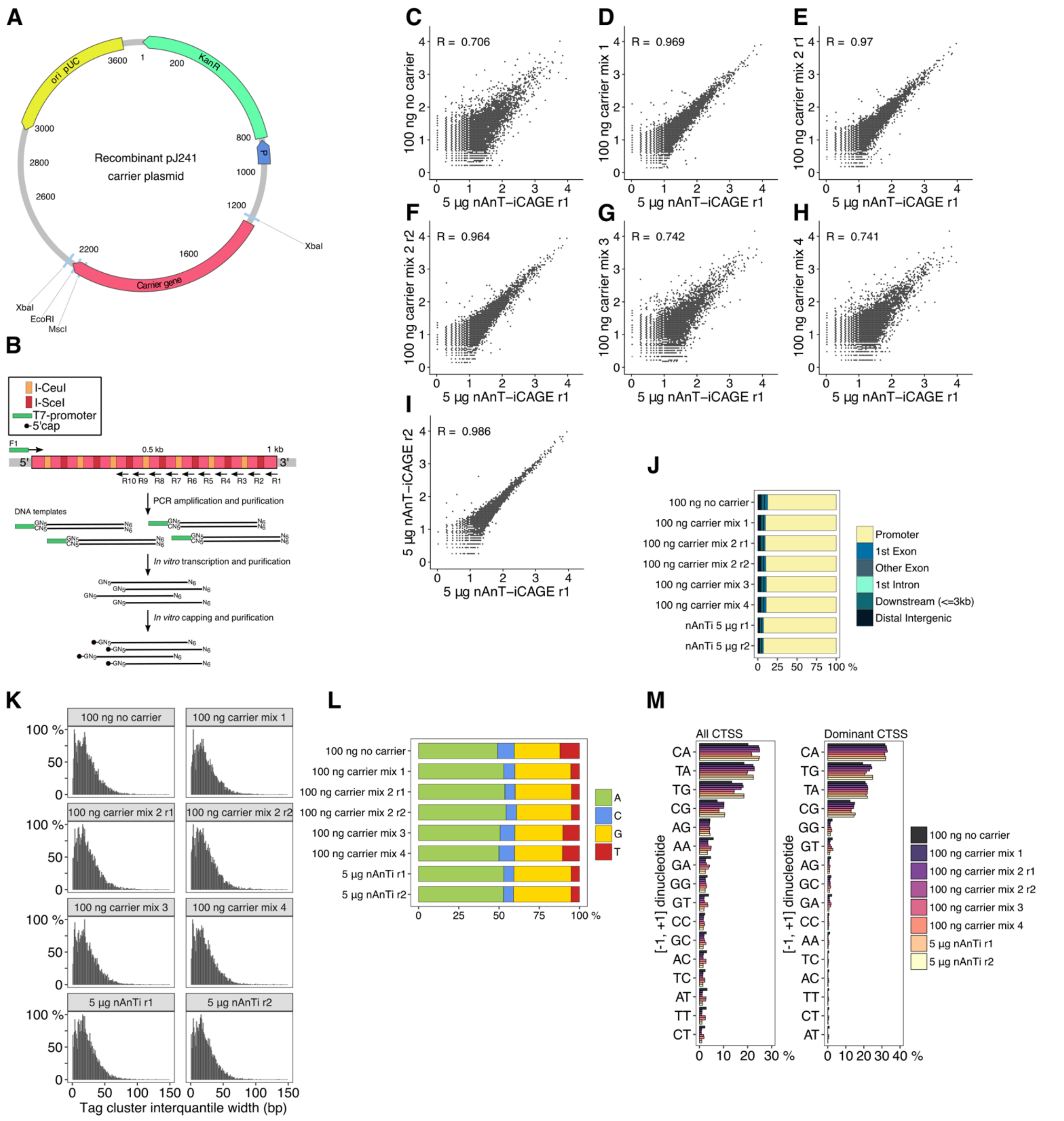

Supplemental Figure S1. Design and test of carrier molecules. (**A**) Schematics of the recombinant plasmid with the synthetic carrier gene. (**B**) Workflow for preparation of the carrier molecules with embedded I-CeuI and I-SceI recognition sites. First, the DNA template for *in vitro* transcription is produced using PCR amplification with a common forward primer (PCR_GN5_f1) and a variety of reverse primers (PCR_N6_r1-r10), to synthesise PCR templates of different lengths (931 – 351 bp, Supplemental Table S2). The forward primer contains the T7-promoter sequence, and a GN_5 sequence (N – random nucleotide). The reverse primer dictates the length of the final carrier and

introduces random nucleotides at the 3'end of carrier molecules (N_6). After PCR-amplification, the templates are gel-purified, and the carrier molecules synthesised using run-off *in vitro* transcription. Carriers are then purified and a portion of it capped, followed by purification. Capped carriers are necessary to ensure that there is carrier left after the cap-trapping step, otherwise all carrier molecules would be eliminated from downstream steps. (**C-H**) Test of various carrier mixes added to 100 ng of *S. cerevisiae* total RNA. Pearson correlation at the CTSS level of the libraries constructed using 100 ng of *S. cerevisiae* total RNA and (**C**) no carrier added, (**D**) mix 1: mix of 931 bp capped (0.5 μg) and 931 bp (4.4 μg) uncapped carrier, (**E**) mix 2: mix of 351-931 bp capped (0.5 μg) and 351-931 bp (4.4 μg) uncapped carrier, replicate 1, (**F**) mix 2: same as in (e), replicate 2, (**G**) mix 3: 931 bp capped (0.5 μg) carrier, (**H**) mix 4: 351-931 bp capped (0.5 μg) carrier. All carrier mixes are presented in detail in the Supplemental Table S4 and S5. The necessity of uncapped molecules is presumably an effect of overall quantity of the carrier added (~ 5 μg compared to 0.5 μg when only capped carrier is used). If only the capped carrier was used in larger quantities (up to 5 μg), it would saturate streptavidin resin and potentially lead to loss of capped target mRNAs. (**I**) Pearson correlation at the CTSS level of two nAnT-iCAGE technical replicates constructed using 5 μg of total *S. cerevisiae* RNA. (**J**) Genomic locations of tag clusters identified in carrier test SLIC-CAGE libraries and the reference nAnT-iCAGE library. (**K**) Distribution of tag cluster interquantile widths in carrier test SLIC-CAGE libraries and the reference nAnT-iCAGE library. (**L**) Nucleotide composition of all CTSSs identified in carrier test SLIC-CAGE libraries and in the reference nAnT-iCAGE library. (**M**) Dinucleotide composition of all CTSSs (left panel) or dominant CTSSs (right panel) identified in carrier test SLIC-CAGE libraries and in the reference nAnT-iCAGE library. Both panels are ordered from the most to least used dinucleotide in the reference nAnT-iCAGE.

Supplemental Figure S2.

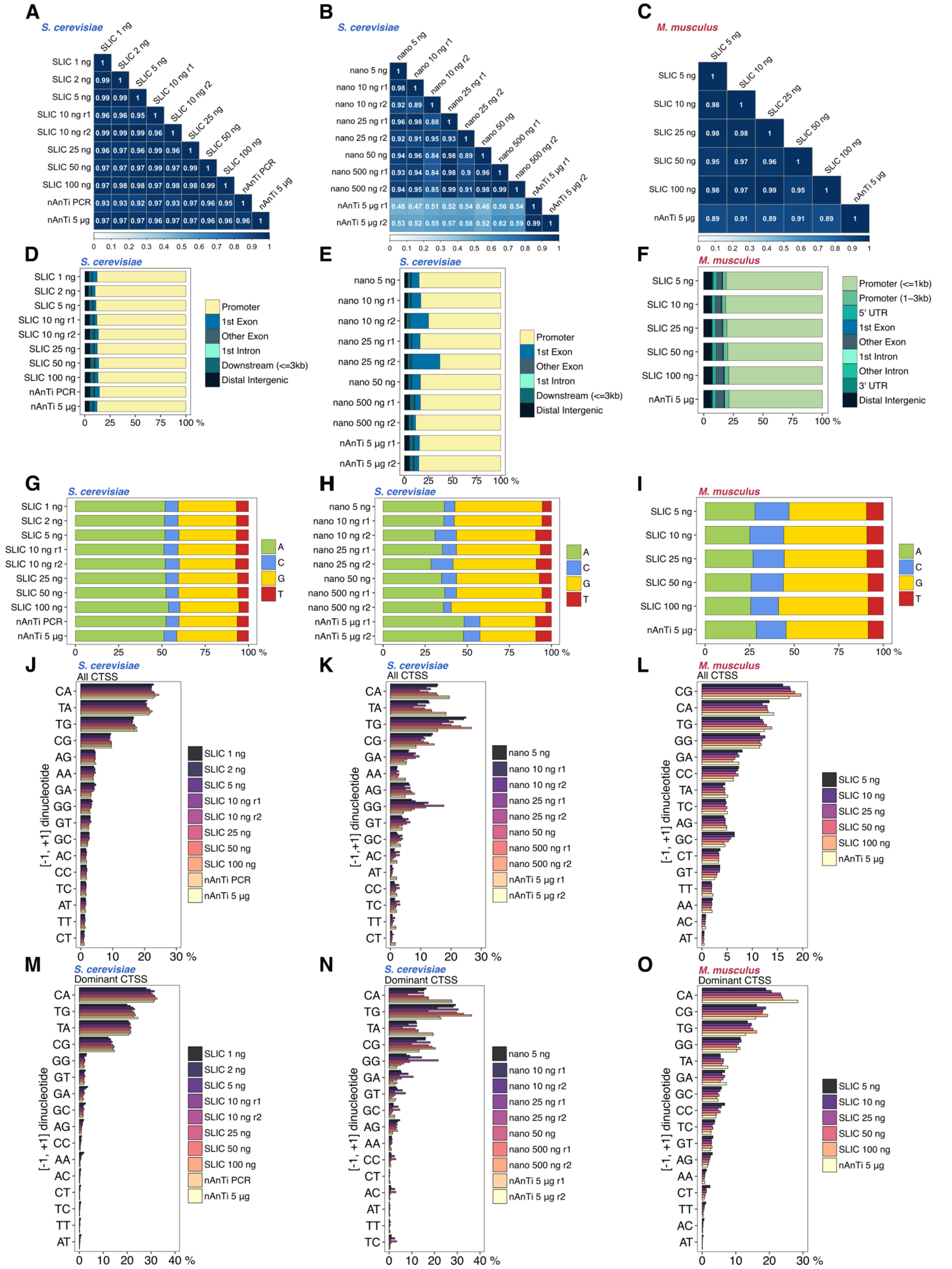

Supplemental Figure S2. Performance comparison of SLIC-CAGE and nanoCAGE libraries. Pearson correlation coefficients at the CTSS level of (**A**) SLIC-CAGE libraries constructed from 1-100 ng of *S. cerevisiae* total RNA and corresponding nAnT-iCAGE libraries (**B**) nanoCAGE libraries constructed from 5-500 ng of *S. cerevisiae* total RNA and the nAnT-iCAGE libraries (**C**) SLIC-CAGE libraries constructed from 5-100 ng of *M. musculus* total RNA and the reference nAnT-iCAGE library. (**D-F**) Genomic locations of tag clusters identified in (**D**) *S. cerevisiae* SLIC-CAGE libraries and the reference nAnT-iCAGE library, (**E**) *S. cerevisiae* nanoCAGE libraries and the reference nAnT-iCAGE library, (**F**) *M. musculus* SLIC-CAGE libraries and the reference nAnT-iCAGE library. (**G-I**) Nucleotide composition of all CTSSs identified in (**G**) *S. cerevisiae* SLIC-CAGE libraries, (**H**) *S. cerevisiae* nanoCAGE libraries, (**I**) *M. musculus* SLIC-CAGE libraries. (**J-L**) Dinucleotide composition of all CTSSs identified in (**J**) *S. cerevisiae* SLIC-CAGE libraries, (**K**) *S. cerevisiae* nanoCAGE libraries, (**L**) *M. musculus* SLIC-CAGE libraries. All panels are ordered from the most to the least used dinucleotide in the reference nAnT-iCAGE. (**M-O**) Dinucleotide composition of dominant CTSSs identified in (**M**) *S. cerevisiae* SLIC-CAGE libraries, (**N**) *S. cerevisiae* nanoCAGE libraries, (**O**) *M. musculus* SLIC-CAGE libraries. All panels are ordered from the most to the least used dominant CTSS dinucleotide in the reference nAnT-iCAGE.

Supplemental Figure S3.

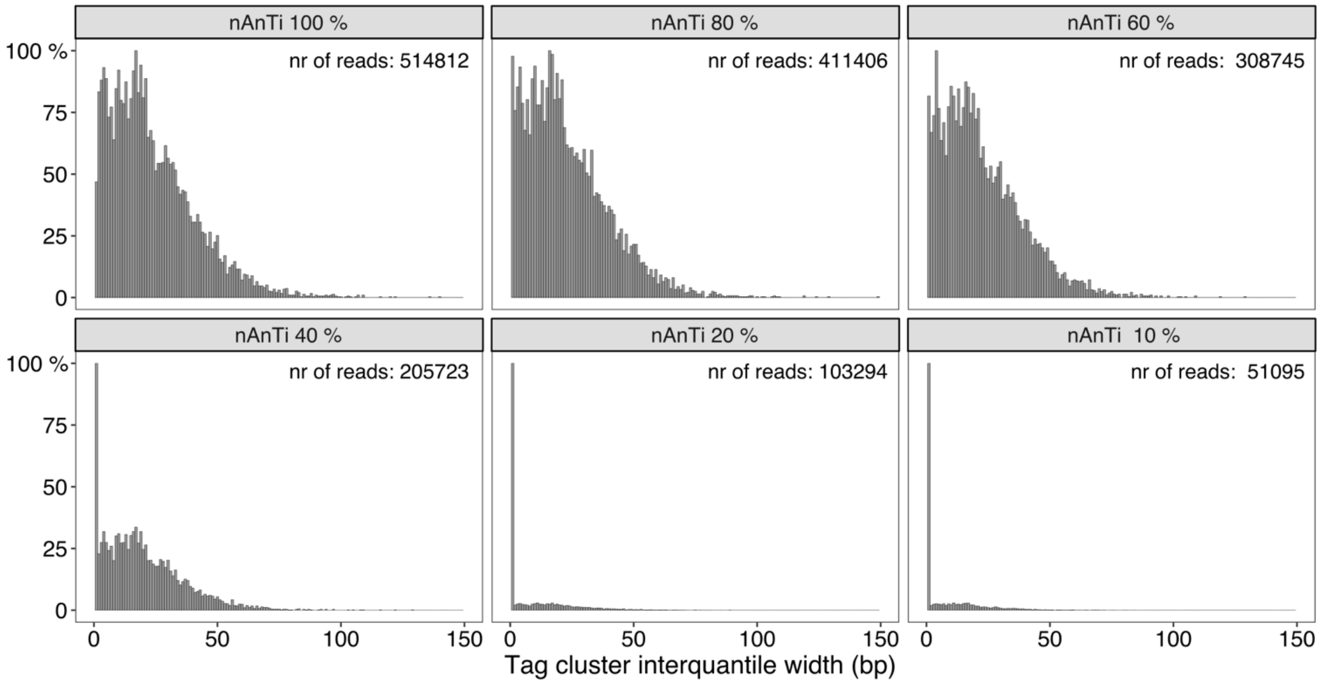

Supplemental Figure S3. Distributions of tag cluster interquantile widths in subsampled *S. cerevisiae* nAnT-iCAGE libraries. All reads (100 %) or 80-10 % of mapped reads were kept in a library. Random subsampling was performed using samtools view -s option. The number of reads kept in the library is indicated in each panel. Subsampling of nAnT-iCAGE library and incomplete CTSS detection leads to artificially sharp libraries.

Supplemental Figure S4.

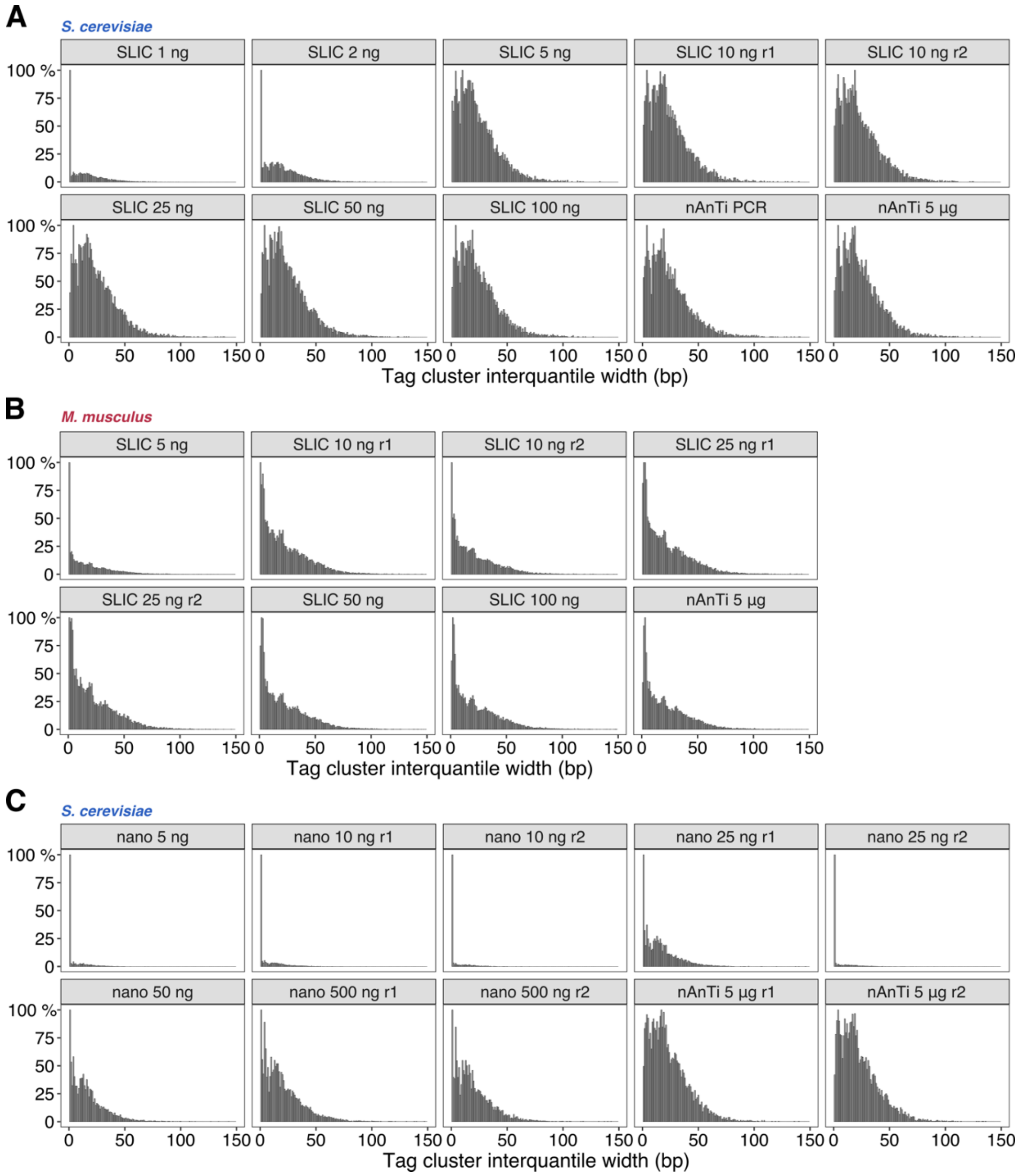

Supplemental Figure S4. Distributions of tag cluster interquantile widths. (**A**) SLIC-CAGE libraries prepared from 1-100 ng of *S. cerevisiae* total RNA in comparison with the nAnT-iCAGE and PCR amplified nAnT-iCAGE library (diluted in water 1:100 and PCR amplified – 13 cycles). (**B**) SLIC-CAGE libraries prepared from 5-100 ng of *M. musculus* total RNA in comparison with nAnT-iCAGE. (**C**) nanoCAGE libraries prepared from 5-500 ng of *S. cerevisiae* total RNA in comparison with nAnT-iCAGE.

Supplemental Figure S5.

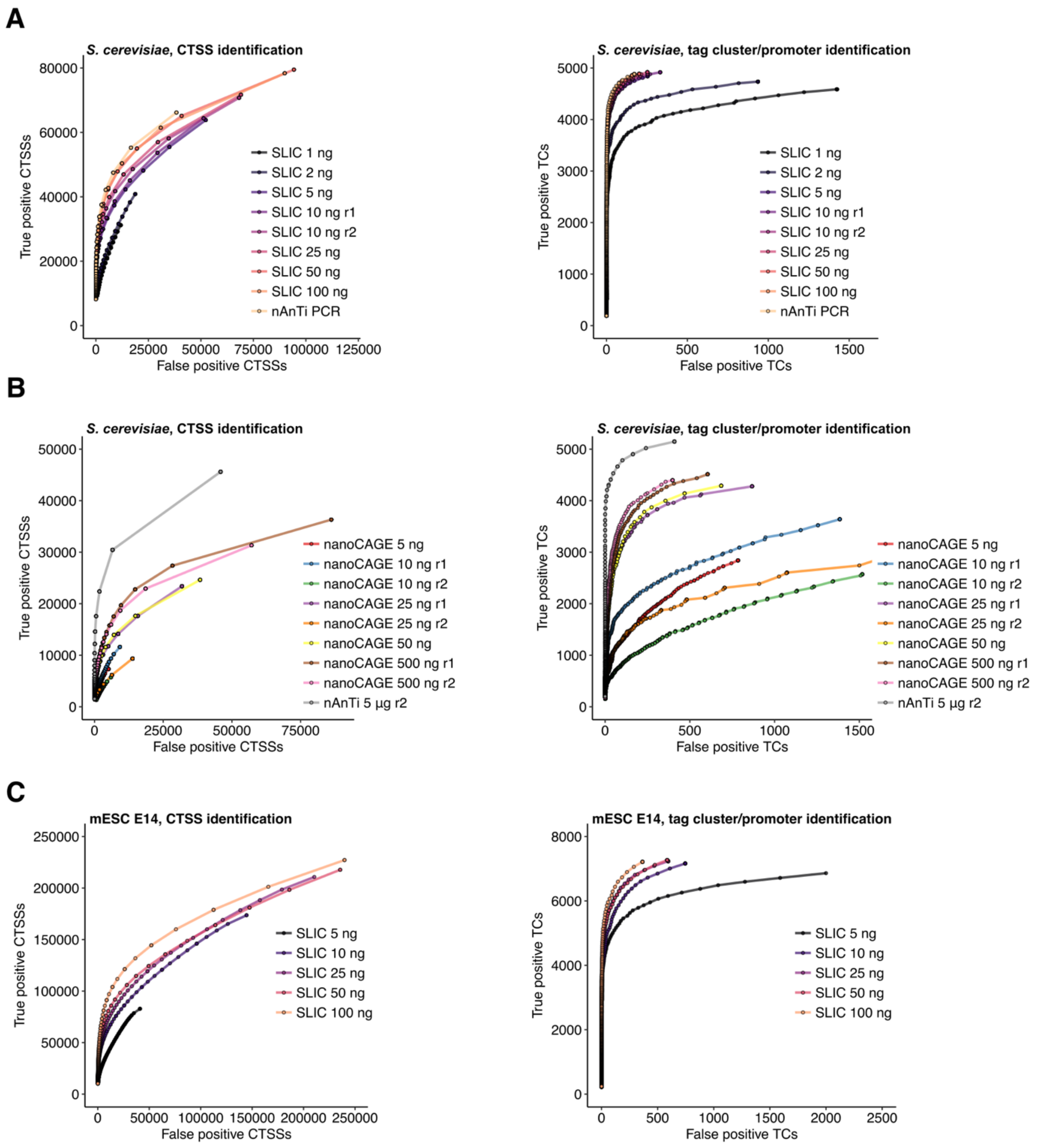

Supplemental Figure S5. ROC curves for samples in SLIC-CAGE and nanoCAGE libraries. (**A**) ROC curves for CTSS (left) or tag cluster (TC) /promoter identification (right) in dependence of CTSS (0-10 TPM) or TC TPM (0-500 TPM) threshold in *S. cerevisiae* SLIC-CAGE libraries. All *S. cerevisiae* nAnT-iCAGE CTSSs and TCs were used as a true set. (**B**) ROC curves for CTSS (left) or TC/promoter identification (right) in dependence of CTSS (0-50 TPM) or TC (0-500 TPM) threshold in *S. cerevisiae* nanoCAGE libraries. All *S. cerevisiae* nAnT-iCAGE CTSSs and TCs were used as a true set. (**C**) ROC curves for CTSS (left) or TC/promoter identification (right) in dependence of CTSS (0-10 TPM) or TC (0-500 TPM) in *M. musculus* SLIC-CAGE libraries. All *M. musculus* nAnT-iCAGE CTSSs and TCs were used as a true set.

Supplemental Figure S6.

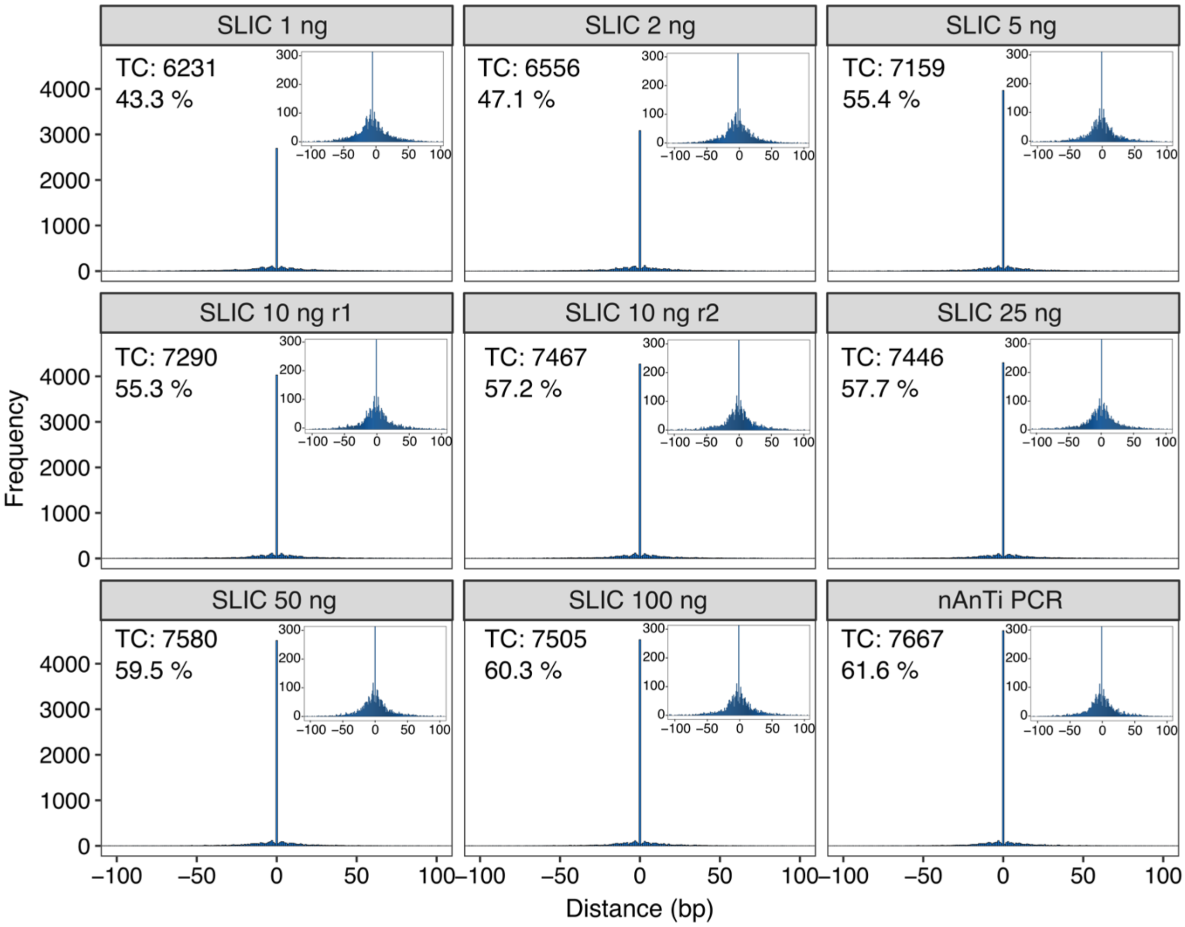

Supplemental Figure S6. Precision of dominant TSS identification in *S. cerevisiae* SLIC-CAGE libraries. Distance distribution between the dominant TSSs in each library and dominant TSSs in nAnT-iCAGE in matched tag clusters. The insets show a magnification of the [-100, 100] region, where 0 is the position of the dominant TSS identified in nAnT-iCAGE library. The label in the upper left corner denotes the number of tag clusters matching nAnT-iCAGE tag clusters and the percentage of its dominant TSSs within 0 bp distance of nAnT-iCAGE-identified dominant TSSs.

Supplemental Figure S7.

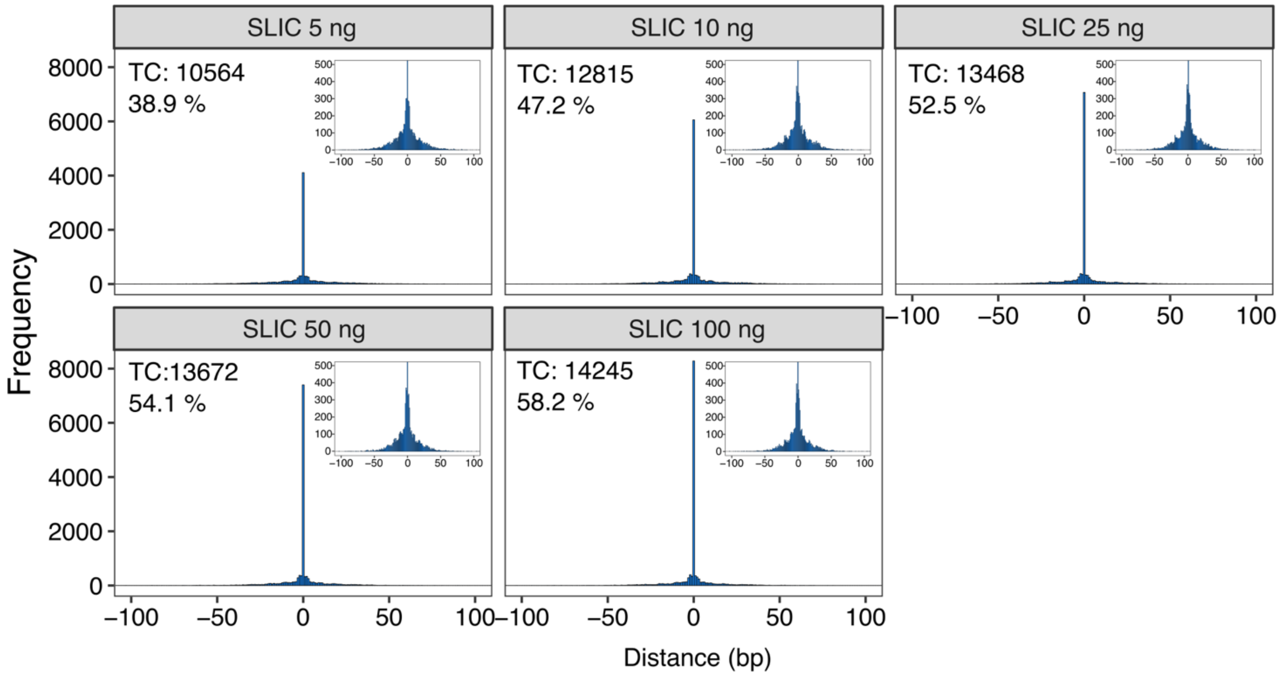

Supplemental Figure S7. Precision of dominant TSS identification in *M. musculus* SLIC-CAGE libraries. Distance distribution between the dominant TSSs in each library and dominant TSSs in nAnT-iCAGE in matched tag clusters. The insets show a magnification of the [-100, 100] region, where 0 is the position of the dominant TSS identified in nAnT-iCAGE library. The label in the upper left corner denotes the number of tag clusters matching nAnT-iCAGE tag clusters and the percentage of its dominant TSSs within 0 bp distance of nAnT-iCAGE-identified dominant TSSs.

Supplemental Figure S8.

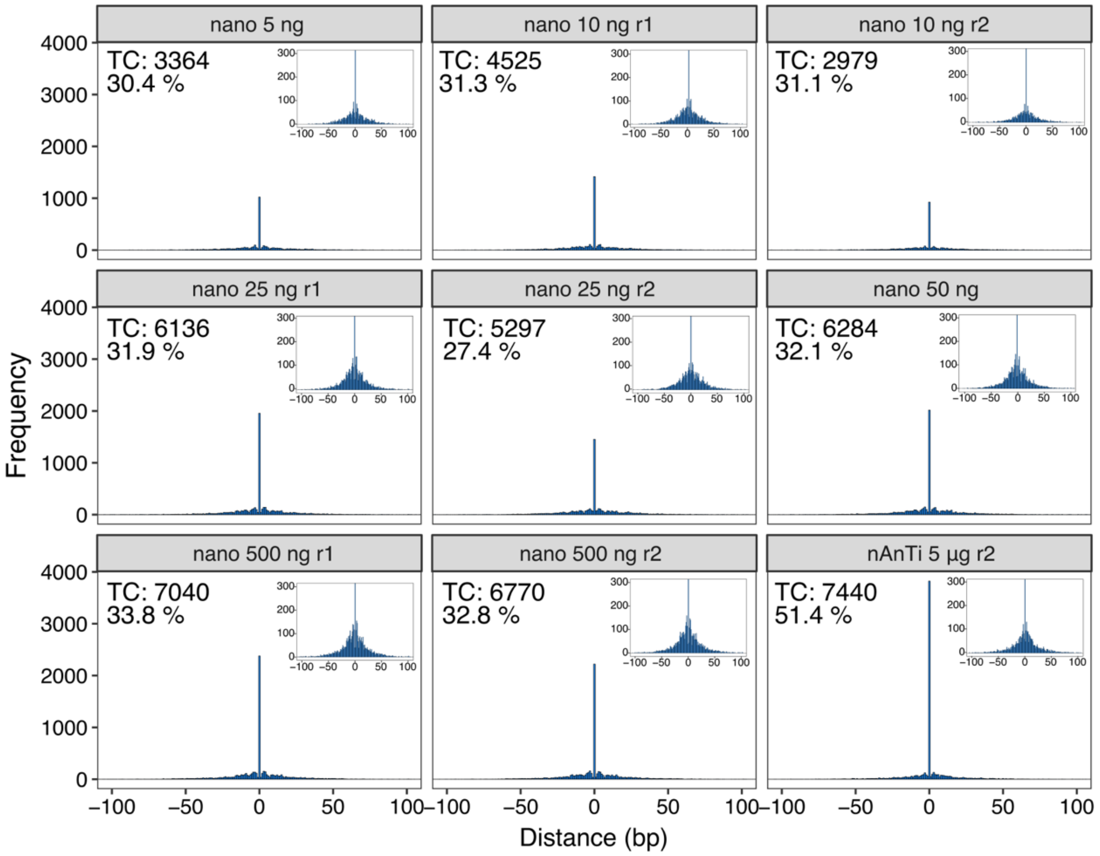

Supplemental Figure S8. Precision of dominant TSS identification in *S. cerevisiae* nanoCAGE libraries. Distance distribution between the dominant TSSs in each library and dominant TSSs in nAnT-iCAGE in matched tag clusters. The insets show a magnification of the [-100, 100] region, where 0 is the position of the dominant TSS identified in nAnT-iCAGE library. The label in the upper left corner denotes the number of tag clusters matching nAnT-iCAGE tag clusters and the percentage of its dominant TSSs within 0 bp distance of nAnT-iCAGE-identified dominant TSSs.

Supplemental Figure S9.

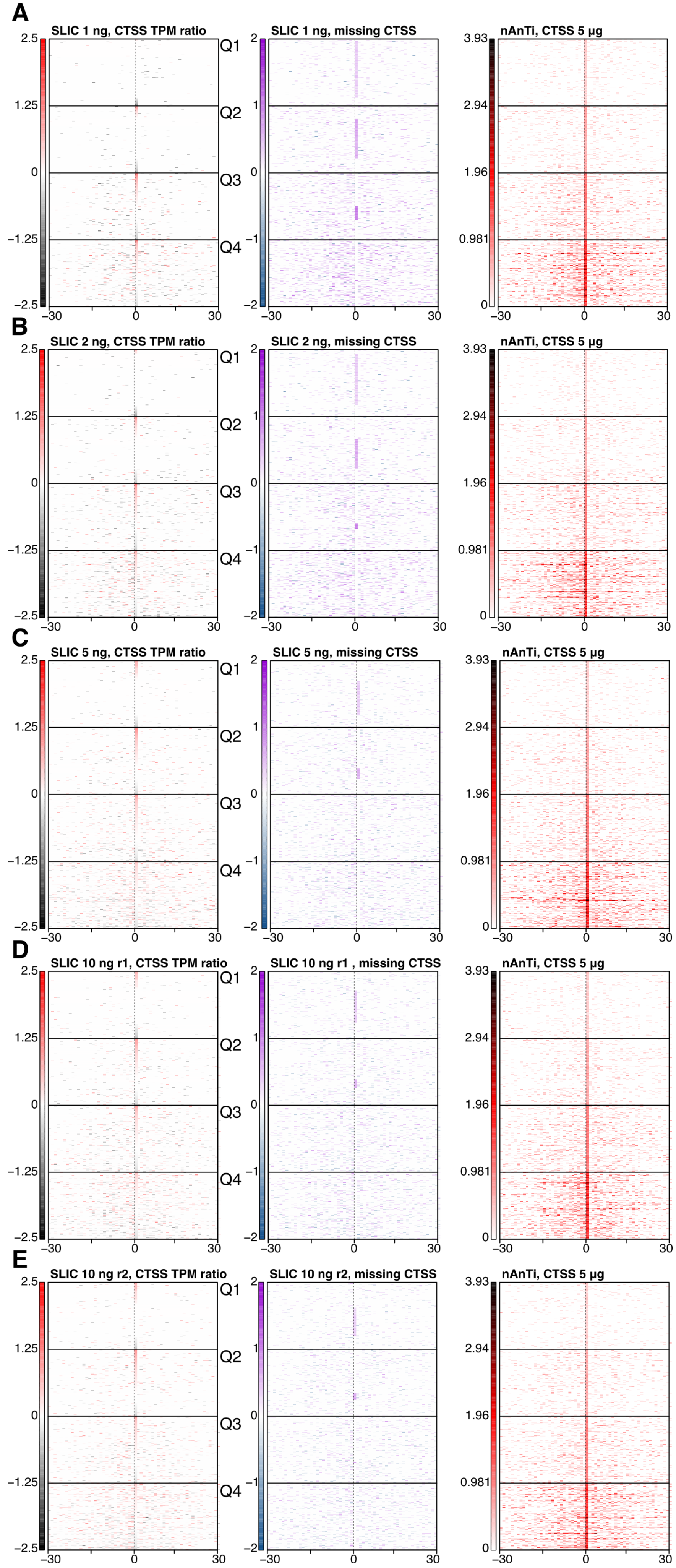

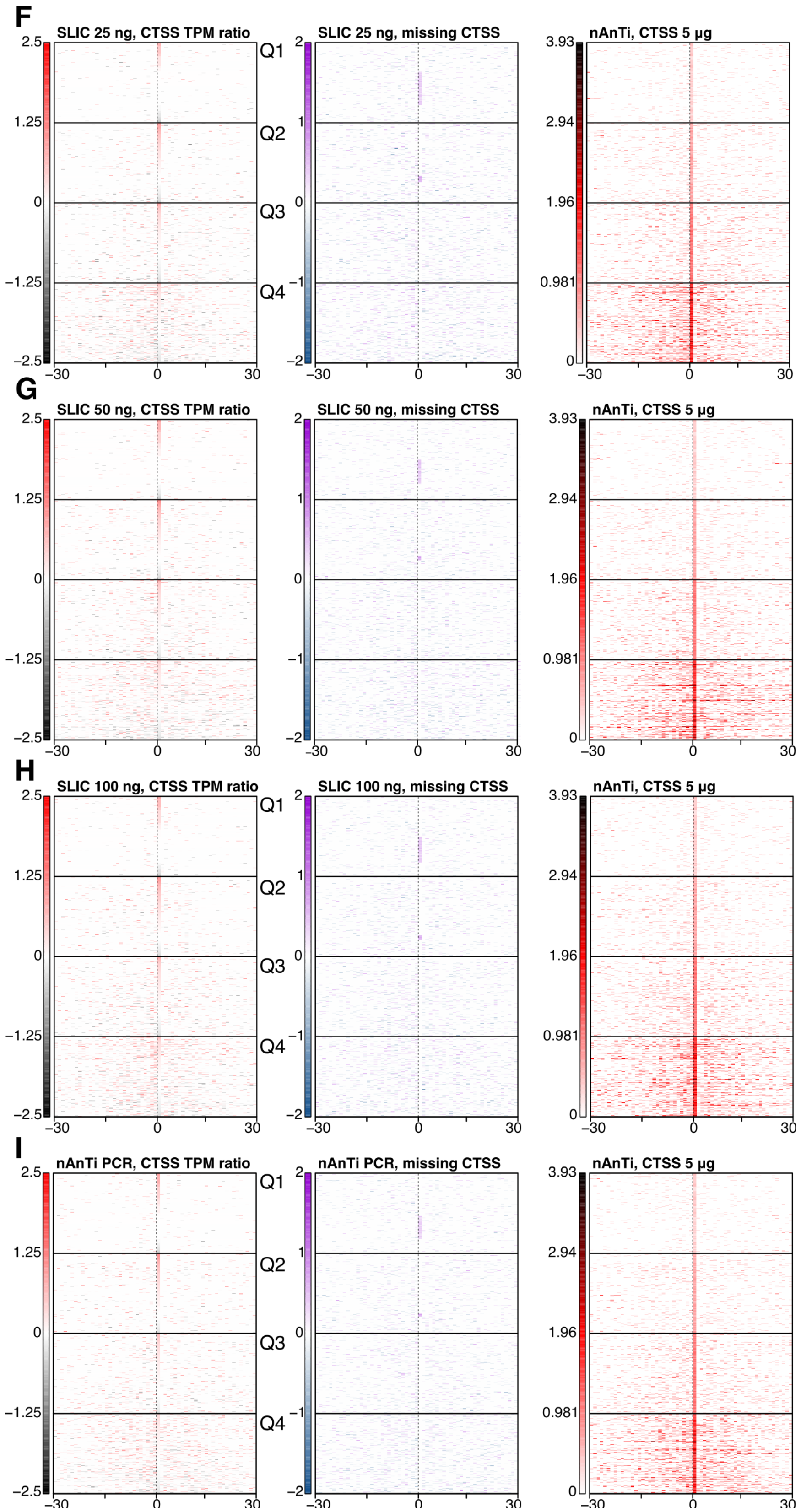

Supplemental Figure S9. Assessment of positional accuracy in *S. cerevisiae* SLIC-CAGE libraries prepared from various amounts of total RNA. (**A**) 1 ng, (**B**) 2 ng, (**C**) 5 ng, (**D**) 10 ng, replicate 1, (**E**) 10 ng, replicate 2, (**F**) 25 ng, (**G**) 50 ng, (**H**) 100 ng, or (**I**) nAnT-iCAGE library prepared from 5 μg of total RNA, diluted 1:100 and PCR amplified. Left panels: heatmaps represent log10(TPM ratio), where the ratio is defined as nAnT-iCAGE TPM value

divided with the corresponding SLIC-CAGE TPM value for each CTSS identified in both libraries. The horizontal lines separate four expression level (TPM) quantiles, with the lowest expression quantile on top (Q1), and the highest at the bottom of the heatmap (Q4). Within each quantile, the sequences are ordered from the highest to the lowest overall TPM ratio values per tag cluster in each SLIC-CAGE library. Middle panels: heatmaps represent the log10(TPM value) of the CTSS present in the nAnT-iCAGE and absent from the SLIC-CAGE library, or the – log10(TPM value) of the CTSS present in the SLIC-CAGE library and absent from the nAnT-iCAGE library. Ordering is the same as explained for left panels. Right panels: coverage of CTSSs present in the reference nAnT-iCAGE library, centred on the dominant CTSS identified in the SLIC-CAGE library with ordering as in the left panels.

Supplemental Figure S10.

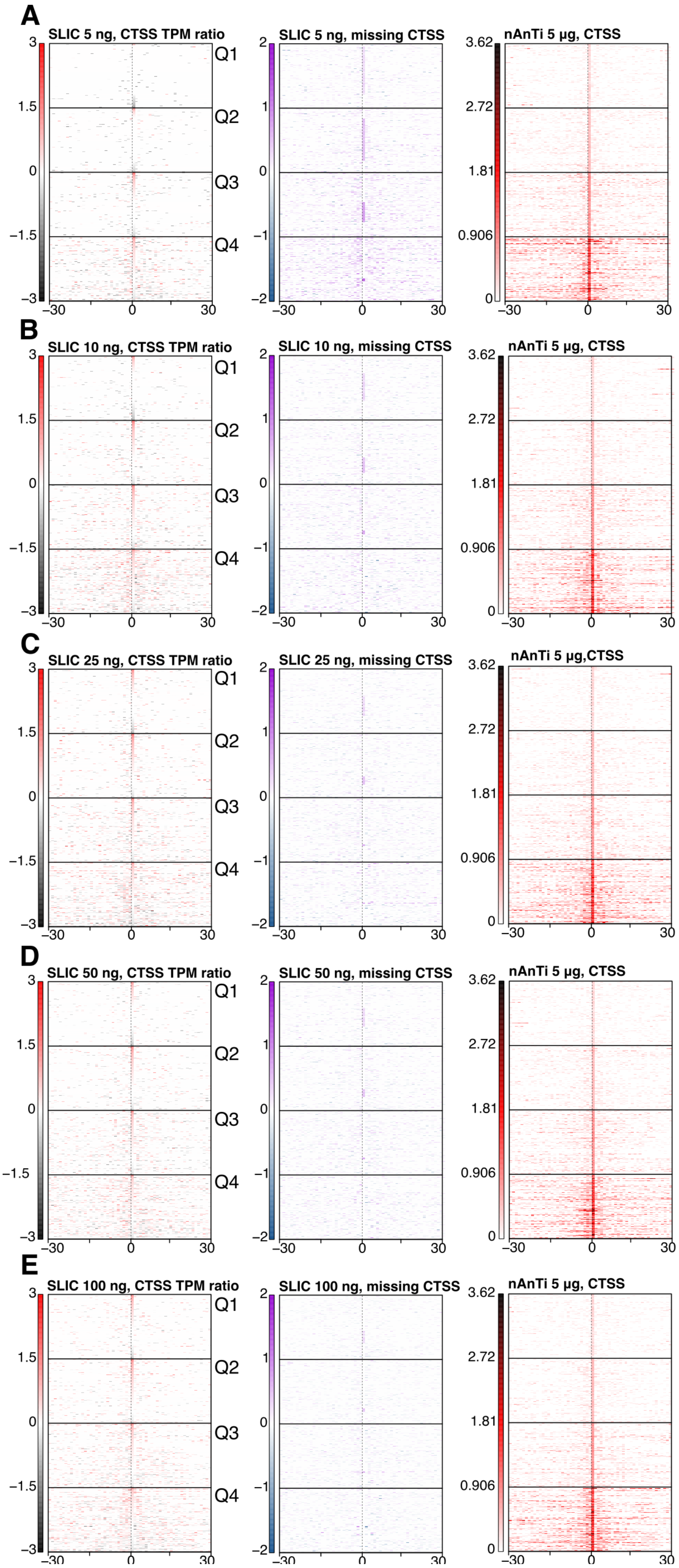

Supplemental Figure S10. Assessment of positional accuracy in *M. musculus* SLIC-CAGE libraries prepared from various amounts of total RNA. (**a**) 5 ng, (**b**) 10 ng, (**c**) 25 ng, (**d**) 50 ng or (**e**) 100 ng. Left panels: heatmaps represent log10(TPM ratio), where the ratio is defined as nAnT-iCAGE TPM value divided with the corresponding SLIC-CAGE TPM value for each CTSS identified in both libraries. The horizontal lines separate four expression level (TPM) quantiles, with the lowest expression quantile on top (Q1), and the highest at the bottom of the heatmap (Q4). Within each quantile, the sequences are ordered from the highest to the lowest overall TPM ratio values per tag cluster in each SLIC-CAGE library. Middle panels: heatmaps represent the log10(TPM value) of the CTSS present in the nAnT-iCAGE and absent from the SLIC-CAGE library, or the –log10(TPM value) of the CTSS present in the SLIC-CAGE library and absent from the nAnT-iCAGE library. Ordering is the same as explained for left panels. Right panels: coverage of CTSSs present in the reference nAnT-iCAGE library, centred on the dominant CTSS identified in the SLIC-CAGE library with ordering as in the left panels.

Supplemental Figure S11.

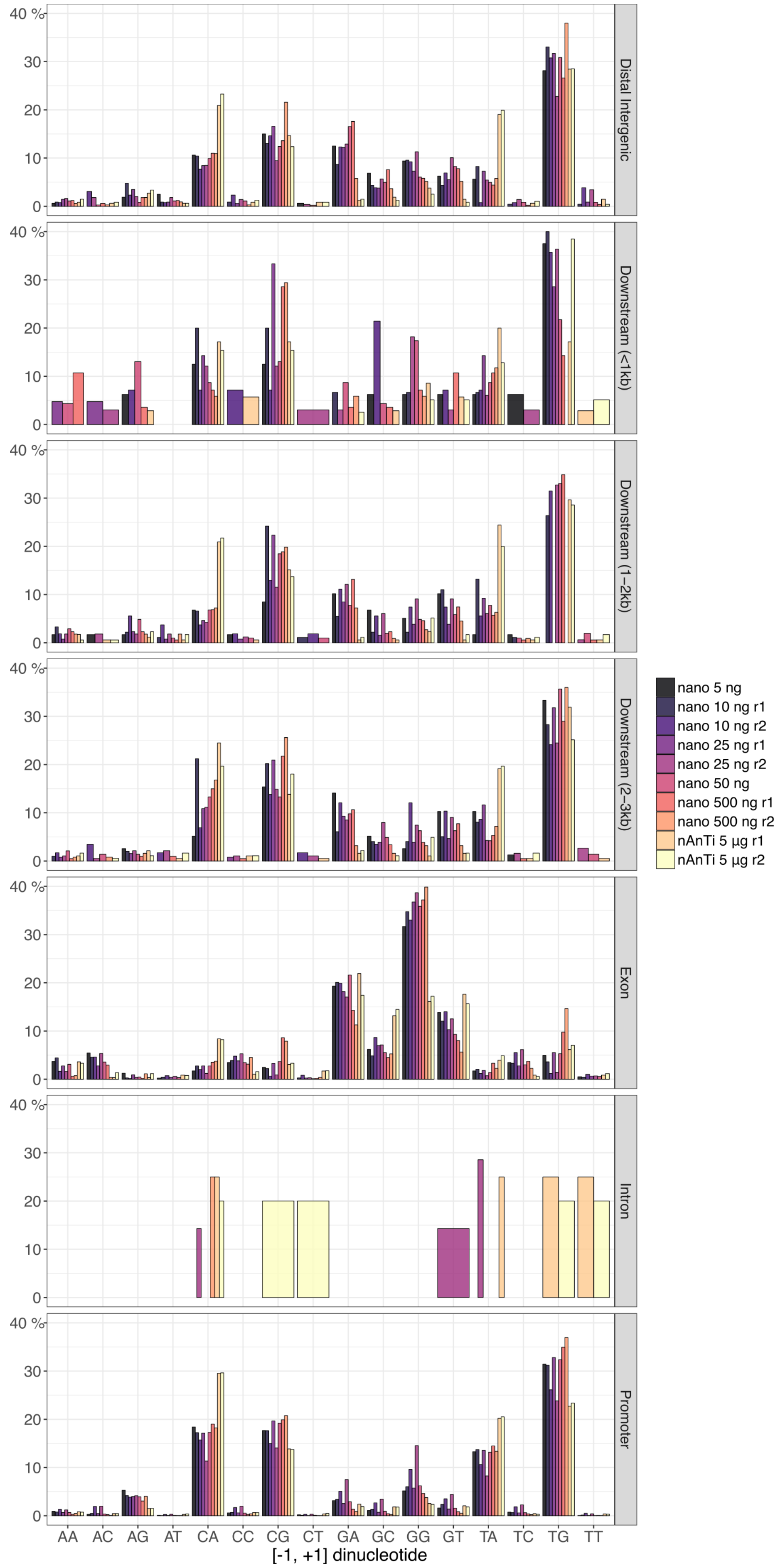

Supplemental Figure S11. Dinucleotide composition of dominant CTSSs. Dominant CTSSs were identified in nanoCAGE libraries derived from 5-500 ng of *S. cerevisiae* total RNA and compared with the nAnT-iCAGE library (derived from 5 μg of total RNA). Dominant CTSSs are split according to genomic locations.

Supplemental Figure S12.

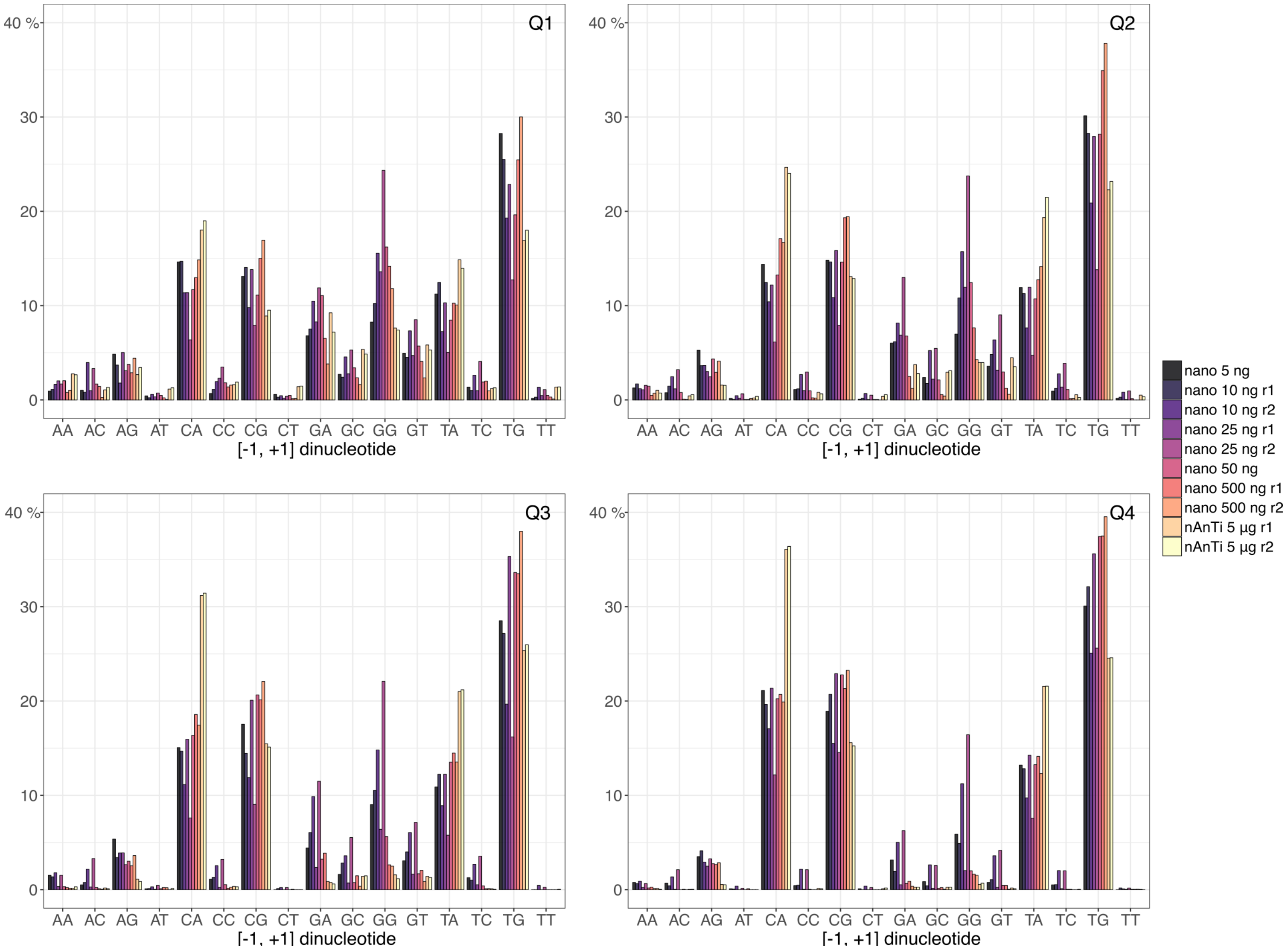

Supplemental Figure S12. Dinucleotide composition of dominant CTSSs. Dominant CTSSs were identified in nanoCAGE libraries derived from 5-500 ng of *S. cerevisiae* total RNA and compared with the nAnT-iCAGE library (derived from 5 µg of total RNA). Dominant CTSSs are split according their expression (TPM) values into quartiles (Q1 – the lowest 25%, Q4 – the highest 25%).

Supplemental Figure S13.

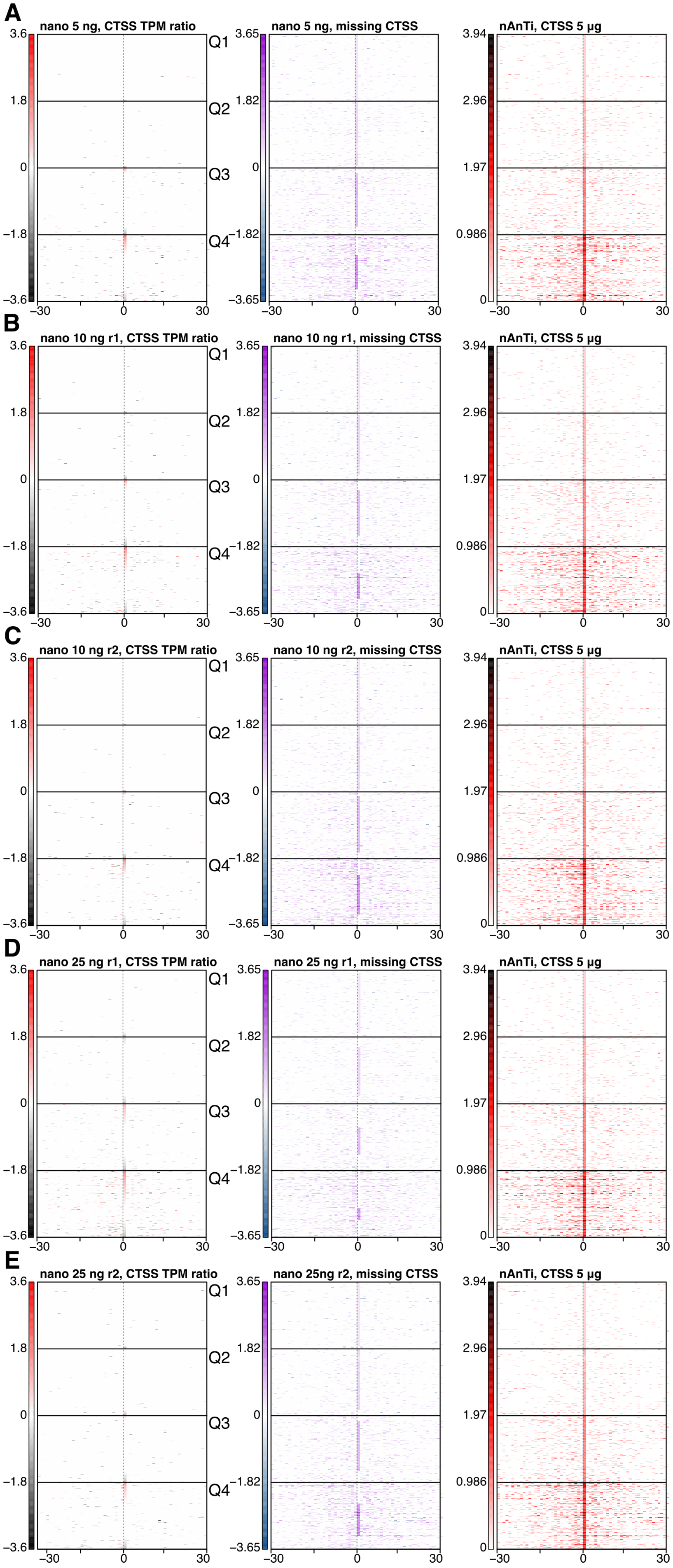

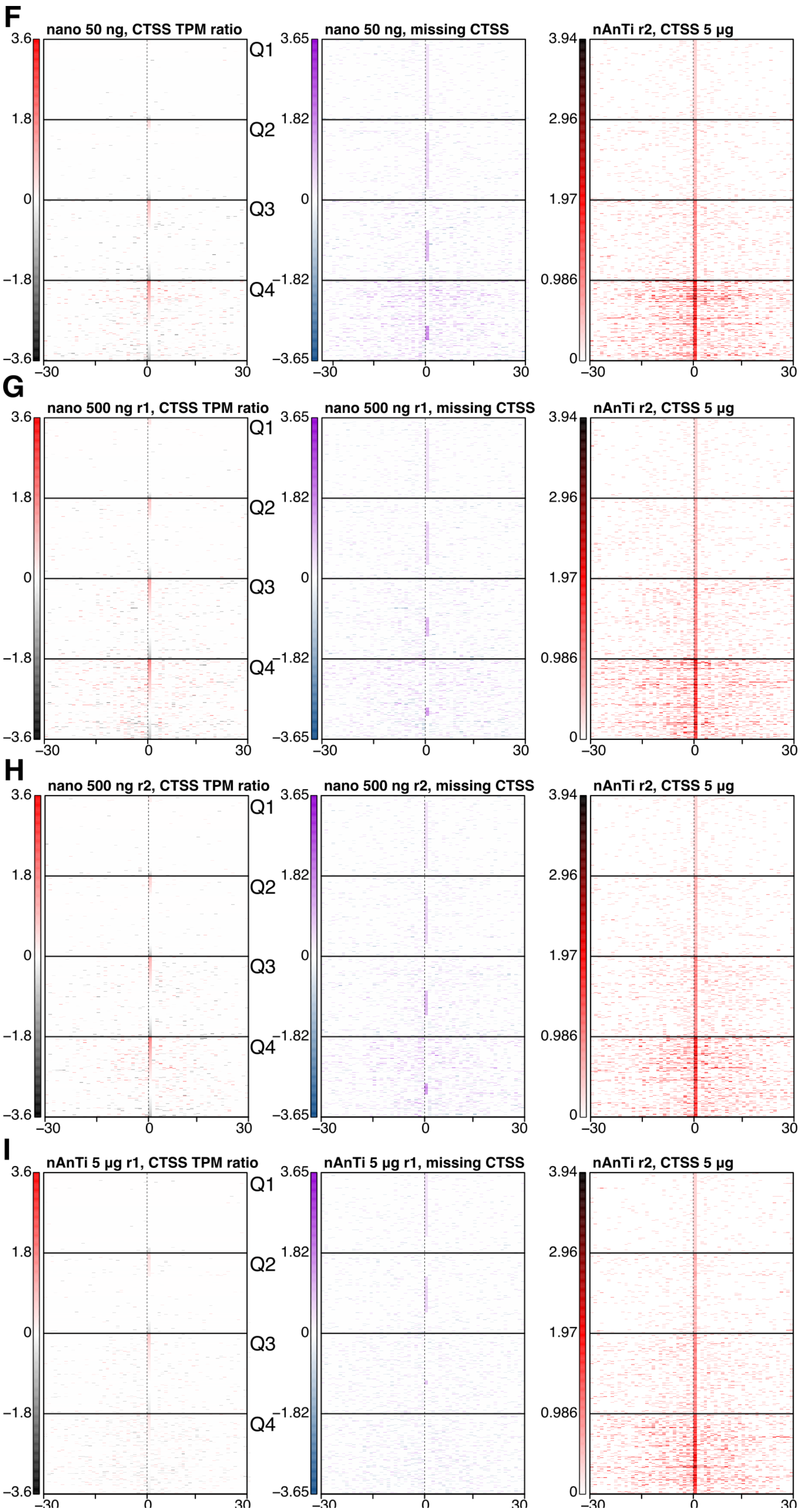

Supplemental Figure S13. Assessment of CTSS positional accuracy in nanoCAGE. Libraries are prepared from various amounts of *S. cerevisiae* total RNA (**A**) 5 ng, (**B**) 10 ng, replicate 1 (**C**) 10 ng, replicate 2 (**D**) 25 ng, replicate 1 (**E**) 25 ng, replicate 2 (**F**) 50 ng, (**G**) 500 ng, replicate 1 (**H**) 500 ng, replicate 2 or (**I**) nAnT-iCAGE library prepared from 5 µg of total RNA, replicate 1. Left panels: heatmaps represent log10(TPM ratio), where the ratio is defined as nAnT-iCAGE TPM value divided with the corresponding nanoCAGE TPM value for each CTSS identified in both

libraries. The horizontal lines separate four expression level (TPM) quantiles, with the lowest expression quantile on top, and the highest at the bottom of the heatmap. Within each quantile, the sequences are ordered from the highest to the lowest overall TPM ratio values per tag cluster in each nanoCAGE library. Middle panels: heatmaps represent the log10(TPM value) of the CTSS present in the nAnT-iCAGE and absent from the nanoCAGE library, or the –log10(TPM value) of the CTSS present in the nanoCAGE library and absent from the nAnT-iCAGE library. Ordering is the same as explained for left panels. Right panels: coverage of CTSSs present in the reference nAnT-iCAGE library, centred on dominant CTSS identified in the nanoCAGE library with ordering as in the left panels.

Supplemental Figure S14.

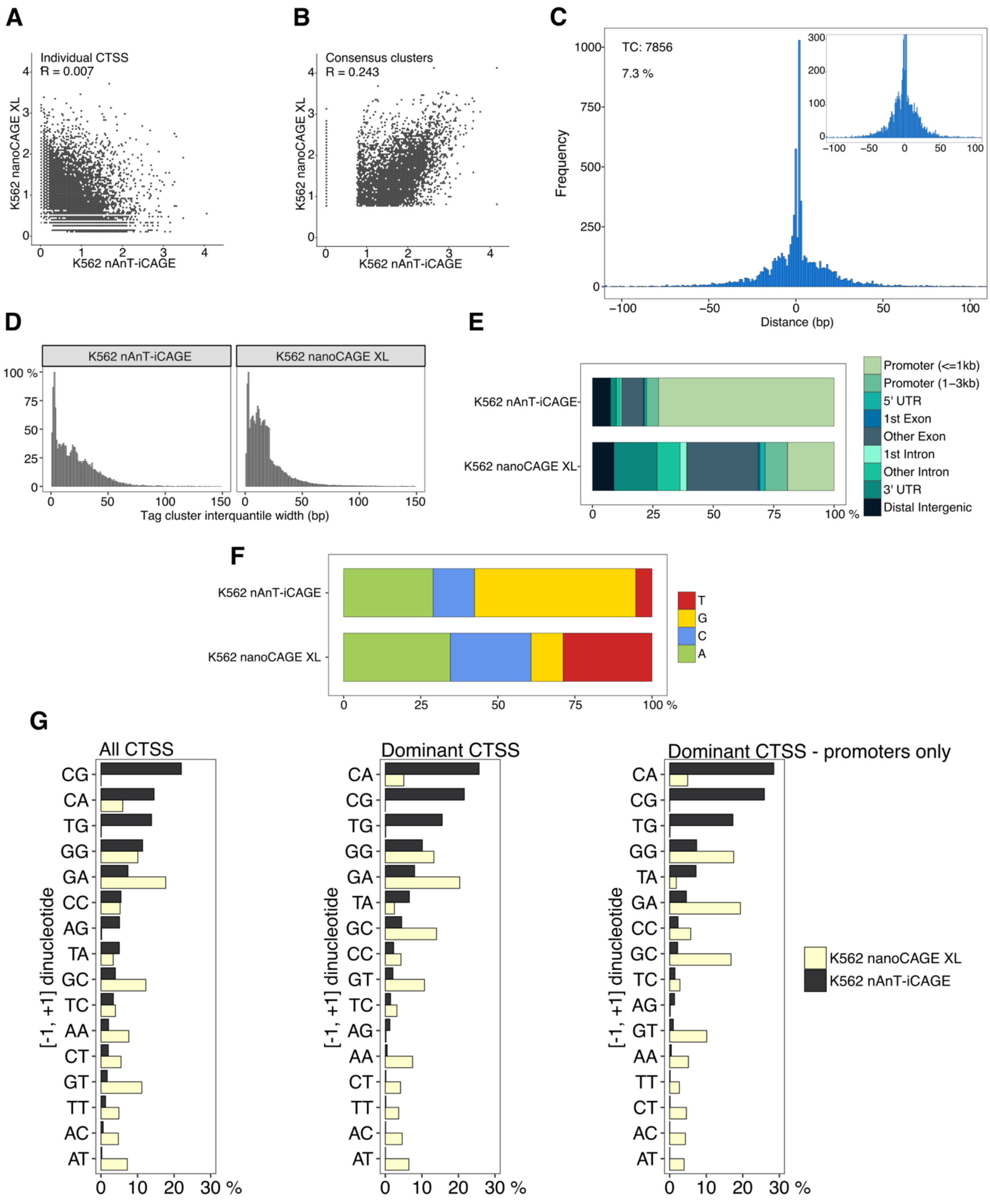

Supplemental Figure S14. Comparison of K562 cell line CAGE and nanoCAGE XL data. Data is from Adiconis et al (Adiconis et al. 2018). (**A**) Pearson correlation of individual CTSS expression levels in nAnT-iCAGE and nanoCAGE XL data. Axes show log10(TPM +1) values and the correlation is calculated on raw non-transformed data. (**B**) Pearson correlation of consensus clusters/promoter expression levels in nAnT-iCAGE and nanoCAGE XL data. (**C**) Distance distribution between the dominant TSSs identified in nanoCAGE XL libraries and dominant TSSs in

nAnT-iCAGE libraries in matched tag clusters. The insets show a magnification of the [-100, 100] region, where 0 is the position of the dominant TSS identified in the nAnT-iCAGE library. The label in the upper left corner denotes the number of tag clusters matching nAnT-iCAGE tag clusters and the percentage of its dominant TSSs within 0 bp distance of nAnT-iCAGE identified dominant TSSs. (**D**) Distribution of tag cluster interquantile widths in nAnT-iCAGE and the nanoCAGE XL library. (**E**) Genomic locations of tag clusters identified in nAnT-iCAGE or nanoCAGE XL K562 libraries. (**F**) Nucleotide composition of all CTSSs identified in nAnT-iCAGE or nanoCAGE XL K562 libraries. (**G**) Dinucleotide composition of all CTSSs (left panel) or dominant CTSSs (middle and right panel) identified in nAnT-iCAGE or nanoCAGE XL K562 libraries. Middle panel includes all identified tag clusters, while the right panel includes tag clusters in promoter regions only (0-3kb distance from UCSC annotated transcription start site). All panels are ordered from the most to least used dinucleotide in nAnT-iCAGE.

Supplemental Figure S15

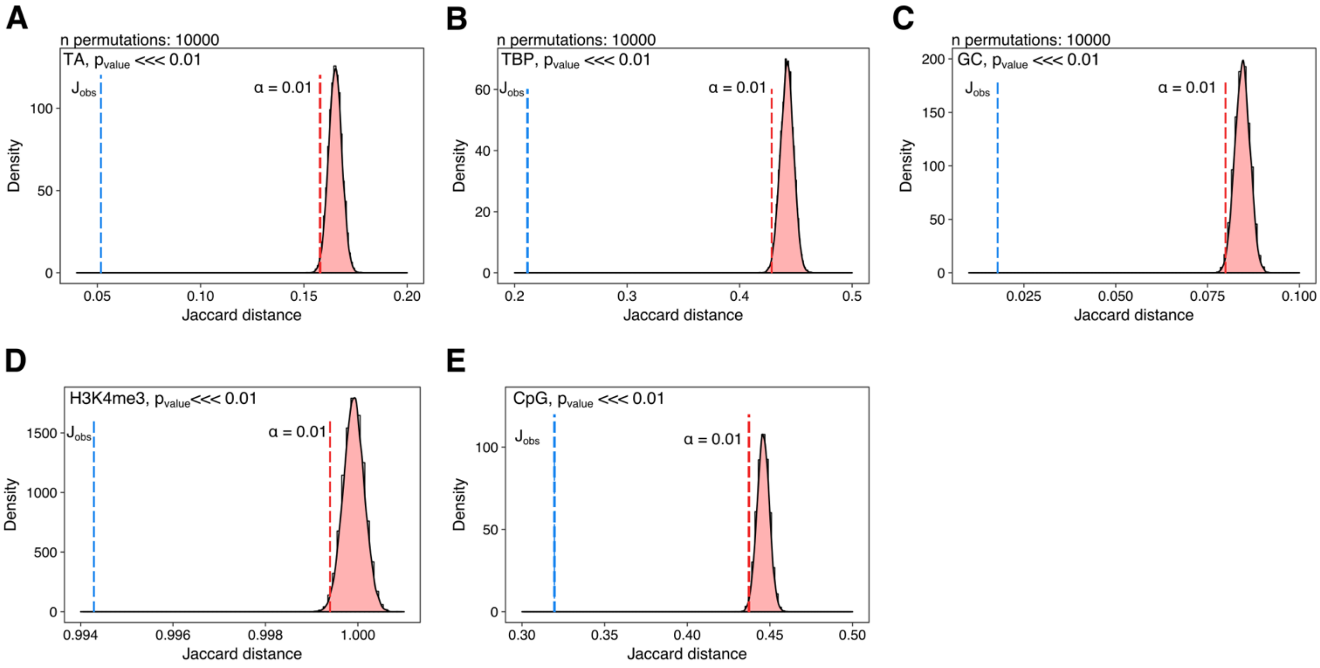

Supplemental Figure S15. Similarity of patterns discovered in mESC E14 SLIC-CAGE 10 ng sample and mESC E14 nAnT-iCAGE 5 µg sample. Jaccard distance (J_{obs}, vertical blue line) shows similarity of vectorized image matrices derived from pattern heatmaps. Background Jaccard distance distribution was calculated from 10000 column permutations of corresponding image matrices. Red vertical line marks the significance cut-off (1st percentile of the distribution). (**A**) TA pattern heatmap, (**B**) TATA-box 80 % PWM match heatmap, (**C)** GC pattern heatmap, (**D**) H3K4me3 coverage heatmap, (**E**) CpG island coverage heatmap.

Supplemental Figure S16.

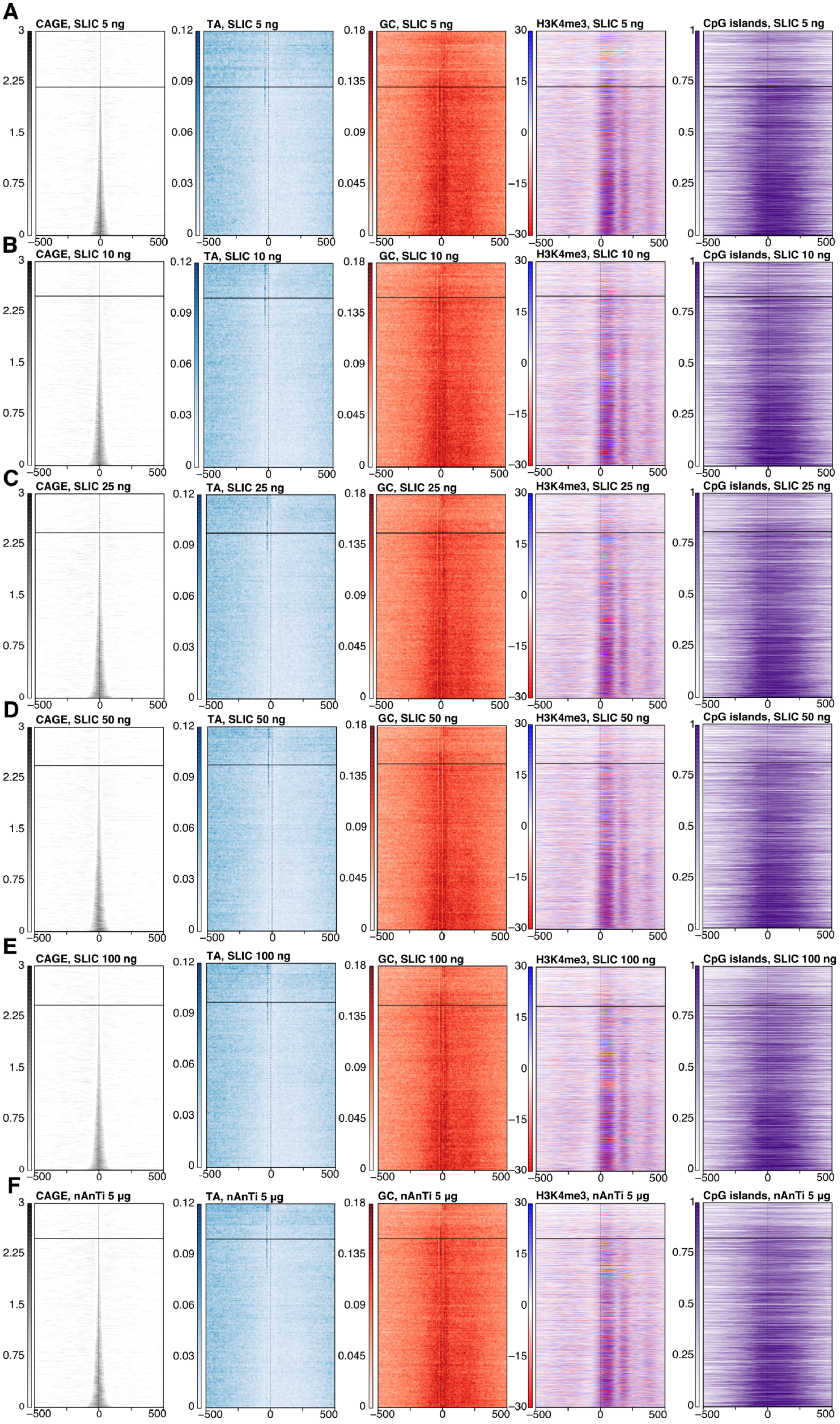

Supplemental Figure S16. Pattern discovery in *M. musculus* SLIC-CAGE libraries. Comparison of CTSS coverage, TA dinucleotide density, GC dinucleotide density, H3K4me3 coverage, CpG islands coverage in SLIC-CAGE libraries prepared from (**A**) 5 ng, (**B**) 10 ng, (**C**) 25 ng, (**D**) 50 ng or (**E**) 100 ng of total RNA and nAnTi-CAGE library prepared from (**F**) 5 µg of total RNA. Windows are centred on the dominant CTSSs identified in SLIC-CAGE or nAnT-iCAGE libraries. Promoter regions are all ordered from sharpest to broadest tag cluster interquantile width. The horizontal line separates sharp and broad promoters (defined by an empirical threshold where interquantile width <= 3 defines sharp, and interquantile width > 3 defines broad promoters). Percentage overlap of CpG islands with TCs in each sample: SLIC 5 ng – 68.1 %, SLIC 10 ng – 68.1%, SLIC 25 ng – 66.6 %, SLIC 50 ng – 65.9 %, SLIC 100 ng – 64.8 %, nAnTi 5 µg – 64.4 %.

Supplemental Figure S17.

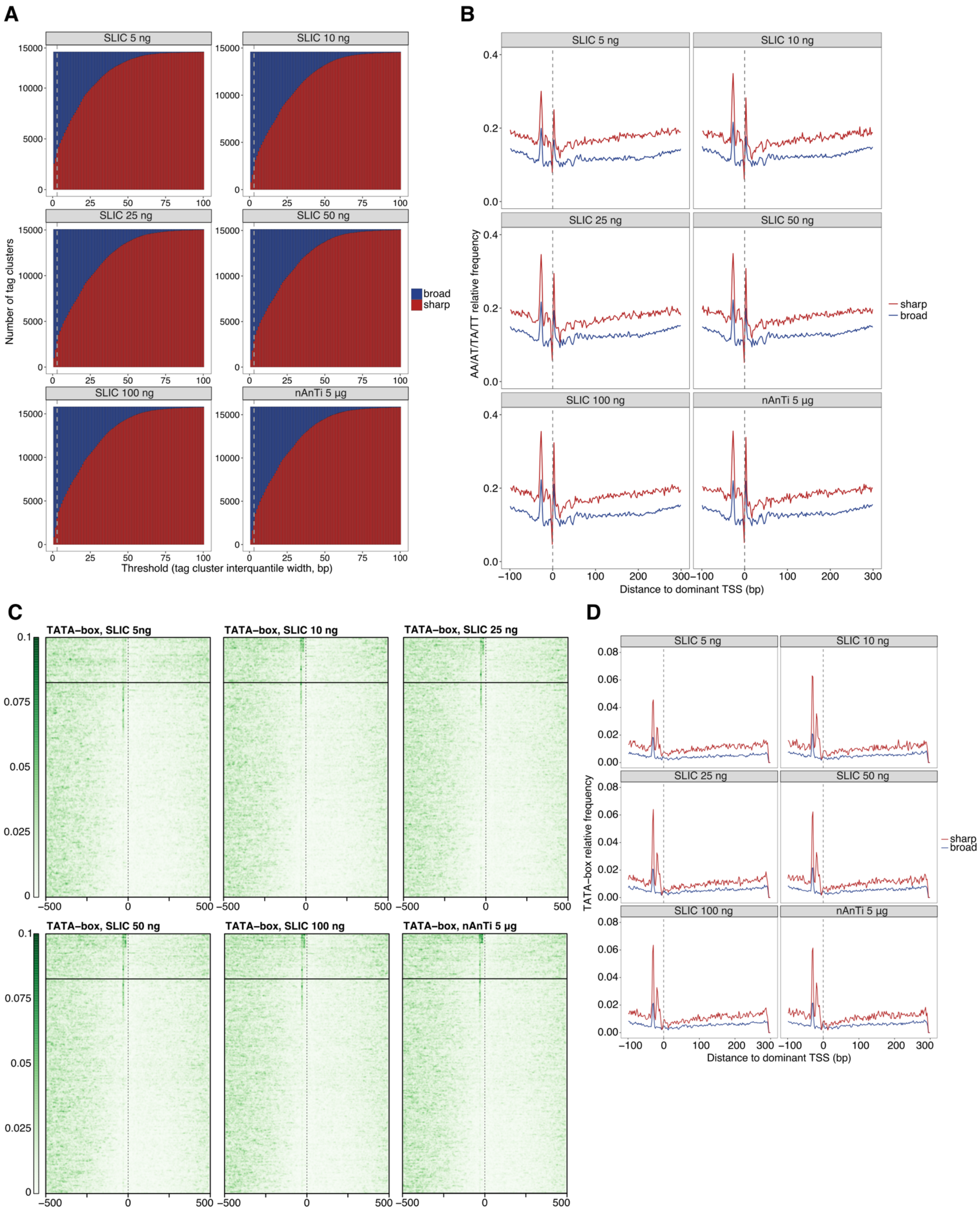

Supplemental Figure S17. Separation of sharp and broad promoters/tag clusters in *M. musculus* SLIC-CAGE libraries. (**A**) Number of sharp or broad tag clusters (y-axis) in dependence of the interquantile width threshold (x-axis). The white dashed vertical line marks the chosen empirical threshold for separating sharp and broad tag clusters/promoters (sharp have interquantile width <= 3 and broad > 3). (**B**) Average AA/AT/TA/TT dinucleotide relative frequency in sharp or broad promoters identified in SLIC-CAGE or nAnT-iCAGE libraries. (**C**) Comparison of TATA-box density in SLIC-CAGE and nAnT-iCAGE libraries. Promoter regions are scanned using a minimum of 80th percentile match to the TATA-box pwm, centred on the dominant TSS and ordered by interquantile width with the sharpest promoters on top of the heatmap, and broadest at the bottom. The horizontal black line separates sharp and broad promoters, defined in (A). Percentage of TCs that have a TATA-box around the -30 positions: SLIC 5 ng – 22.6 %, SLIC 10 ng – 23.6 %, SLIC 25 ng -23.1 %, SLIC 50 ng – 24.3 %, SLIC 100 ng – 23.8 %, nAnTi – 24.1 %. (**D**) TATA-box relative frequency in sharp or broad promoters.

Supplemental Figure S18.

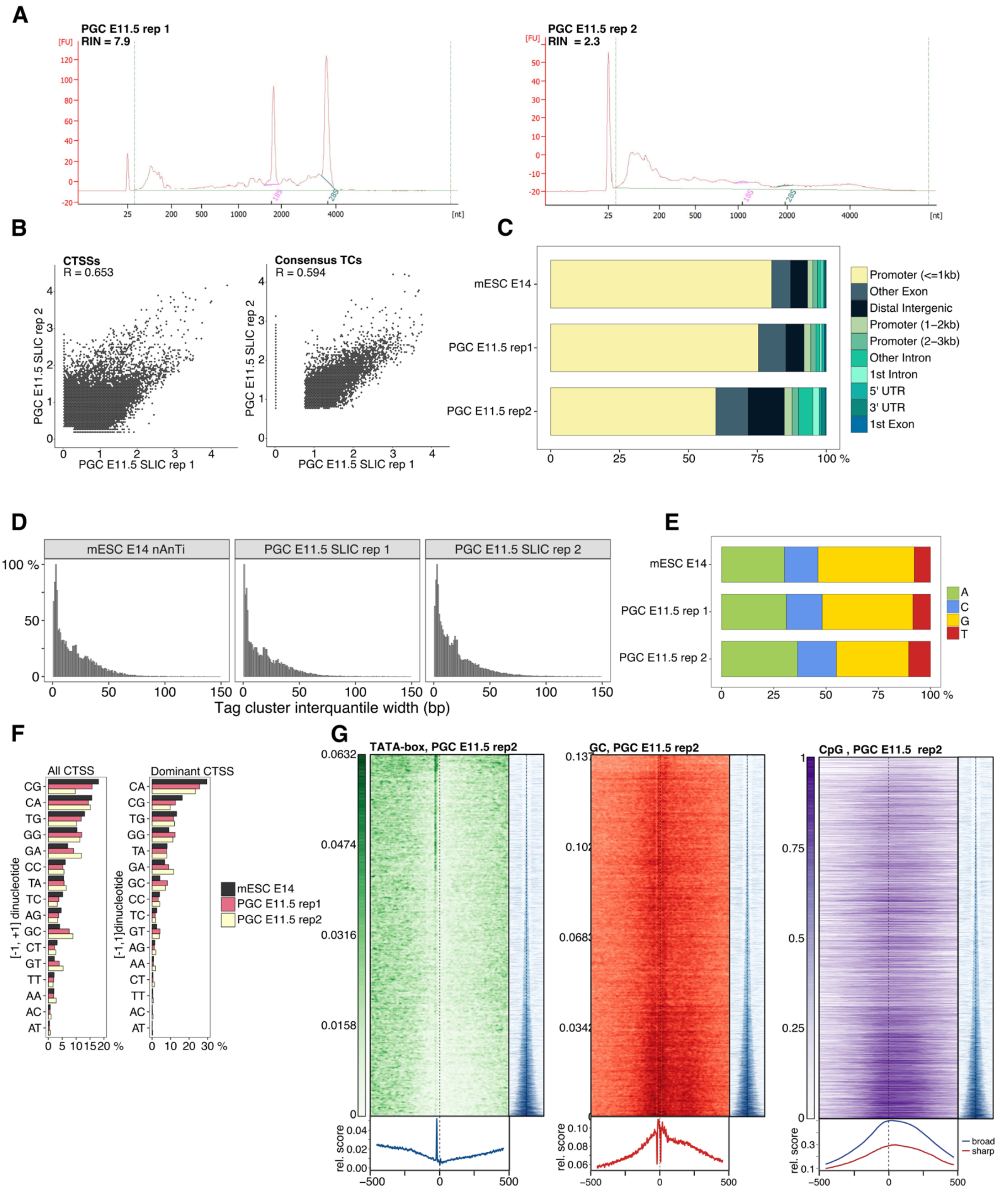

Supplemental Figure S18. Validation of PGC E11.5 SLIC-CAGE libraries. (**A**) Bioanalyzer trace of total RNA isolated from PGC E11.5 replicate 1 (left) or PGC E11.5 replicate 2 (right). (**B**) Pearson correlation of CTSS (left) or

tag cluster expression (right) in PGC E11.5 replicates. The axes are log10(TPM+1) values and the correlation was calculated on raw non-log transformed data. (C-F) Validation of mESC E14 nAnTi and PGC E11.5 SLI-CAGE replicates: (**C**) Genomic locations of identified tag clusters, (**D**) Distributions of tag cluster interquantile widths. (**E**) Nucleotide composition of all identified CTSSs. (**F**) Dinucleotide composition of all CTSSs (left panel) or dominant CTSSs (right panel). Both panels are ordered from the most to the least used dinucleotide in mESC E14 nAnT-iCAGE. (**G**) TATA-box, GC dinucleotide and CpG island density in PGC E11.5 biological replicate. In all heatmaps, promoters are centred at the dominant CTSS (dashed vertical line at 0). Promoter regions are scanned using a minimum of 80th percentile match to the TATA-box PWM. The signal metaplot is shown below each heatmap, and a tag cluster IQ-width coverage (in blue) shows ordering in the pattern heatmap from sharp to broad tag clusters/promoters (200 bp window centred on dominant TSS).

Supplemental Figure S19.

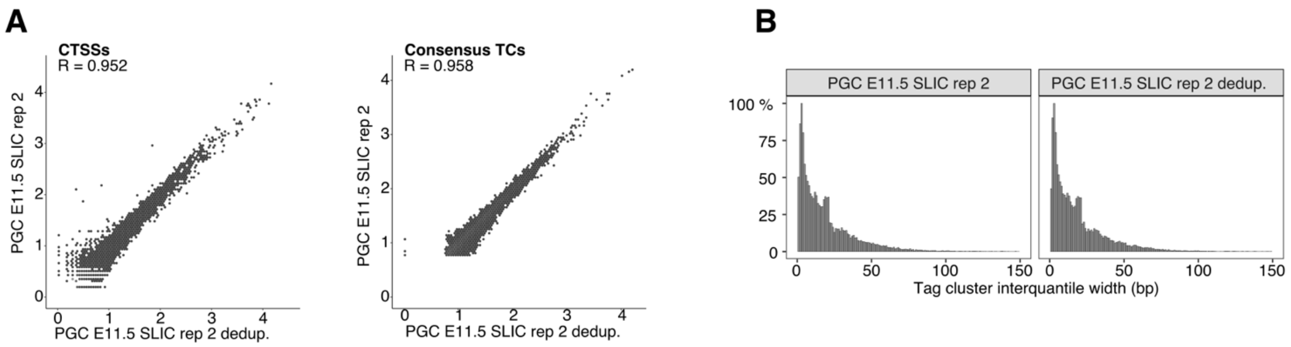

Supplemental Figure S19. Correlation of SLIC-CAGE libraries prior to and after deduplication.

(**A**) Pearson correlation of CTSS (left) or tag cluster expression (right) in PGC E11.5 replicate 2 before and after deduplication. The axes are log10(TPM+1) values and the correlation was calculated on raw non-log transformed data. (**B**) Distributions of tag cluster interquantile widths in PGC E11.5 replicate 2 before (left) and after deduplication (right).

Supplemental Figure S20.

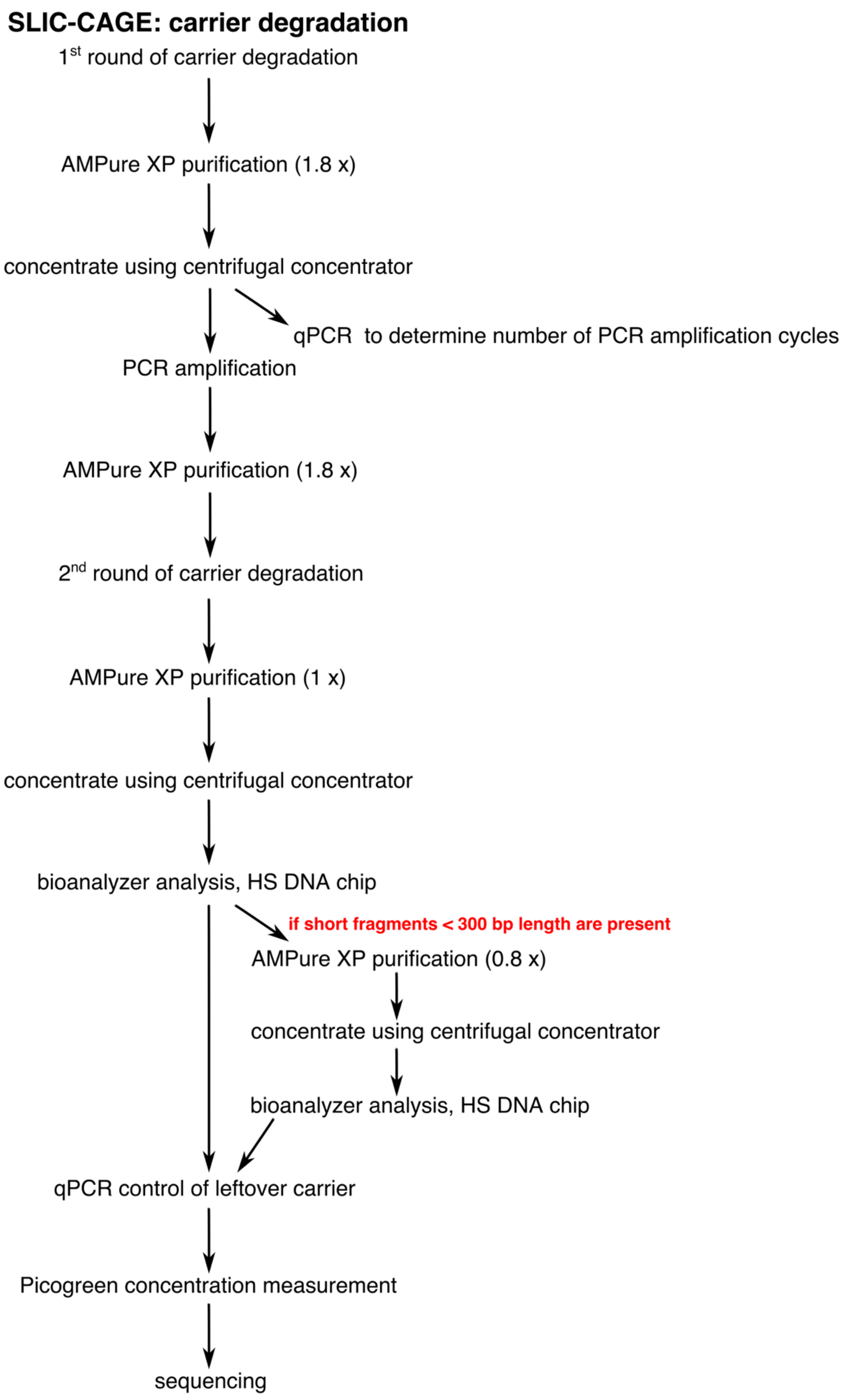

Supplemental Figure S20. Detailed workflow of SLIC-CAGE protocol steps following carrier degradation.

Supplemental Figure S21.

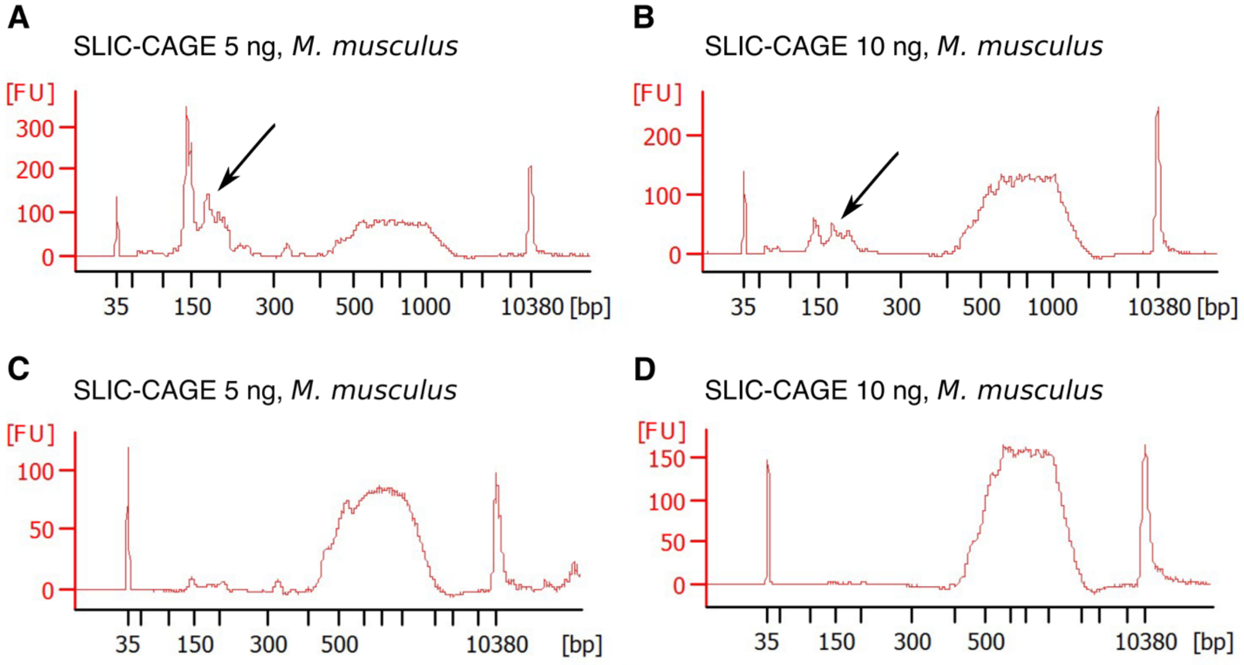

Supplemental Figure S21. Representative SLIC-CAGE HS DNA bioanalyzer traces. Libraries are prepared from 5 or 10 ng of *M. musculus* total RNA after carrier degradation and PCR amplification steps: (**A**, **B**) prior to 2nd round of AMPure XP size selection; (**C, D**) final SLIC-CAGE library after 2nd round of AMPure XP size selection.

Supplemental Figure S22.

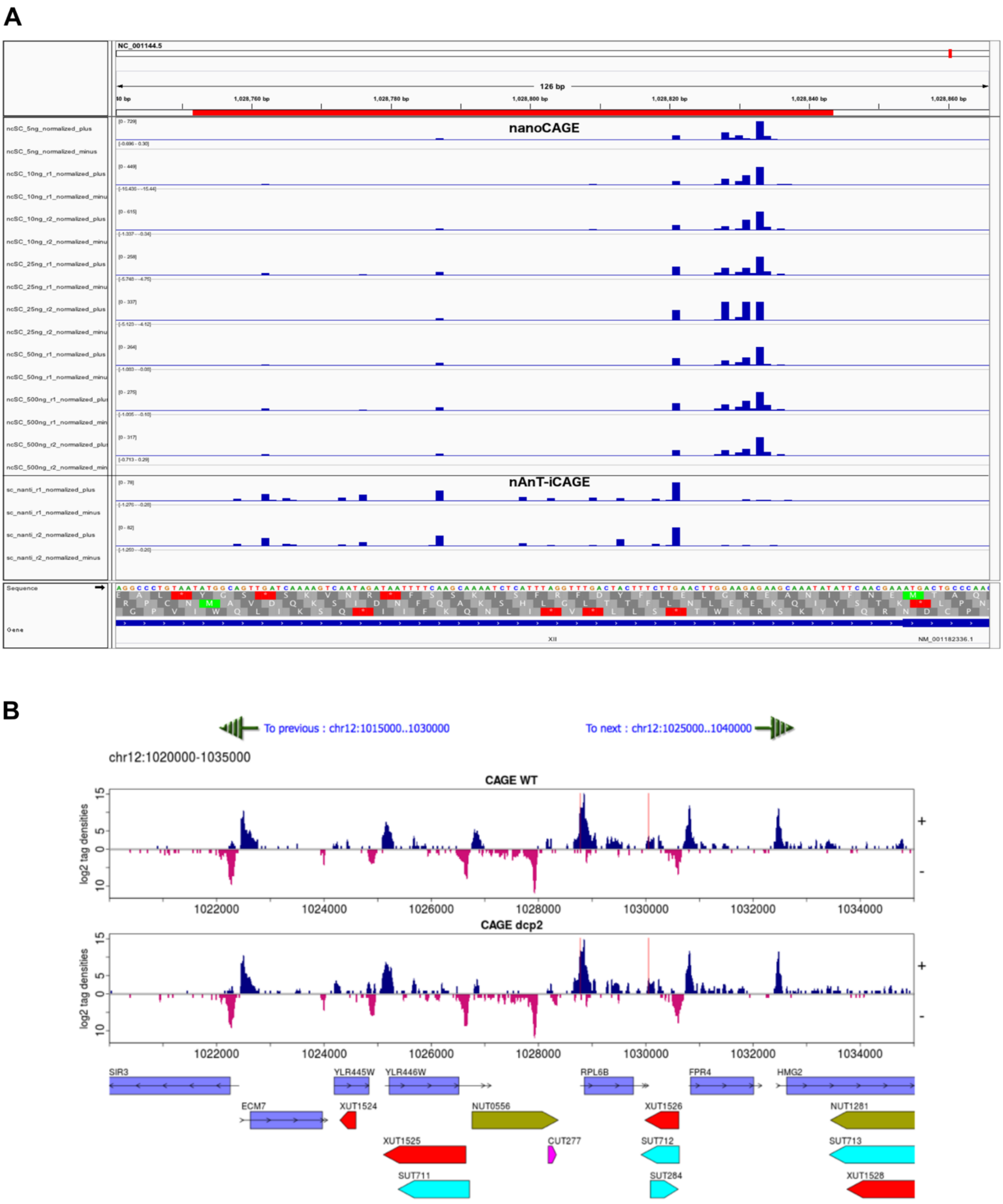

Supplemental Figure S22. Genome browser screenshots of RPL6B locus. (**A**) The screenshot shows promoter region around the *RPL6B* gene in nanoCAGE and nAnT-iCAGE data. The underlying sequence beneath the nanoCAGE tracks (top 8 tracks) shows that the nanoCAGE transcription starts sites are G-rich. Preferential capture of TSSs in G-rich regions explains why nanoCAGE-identified RPL6B tag cluster is more focused and downstream of the canocnial nAnT-iCAGE-identified tag cluster. (**B**) Genome browser

screenshot of RPL6B locus using data from Wery et al 2016 shows broad distribution, confirming our nAnT-iCAGE results in A) (note that these are called CAGE peaks/tag-clusters and not individual CTSSs represented in the screenshot). RPL6B is a component of translation machinery. Studies in Drosophila and mammals have shown that translation machinery components have distinct promoter architecture comprised of a pyrimidine-rich TCT initiator in place of the canonical CA. These promoters usually exhibit sharp transcription initiation and typically do not contain a TATA-box, although an occasional TATA-box may be present. The role of TATA-boxes within TCT promoters is still unclear (for detailed review see (Haberle and Lenhard 2016)). In contrast, *S. cerevisiae* promoter architecture is largely unexplored, most promoters are broad, and TATA-boxes do not have a canonical function as in Metazoan promoters (Cvetesic *et al*, manuscript in preparation).

Supplemental Figure S23.

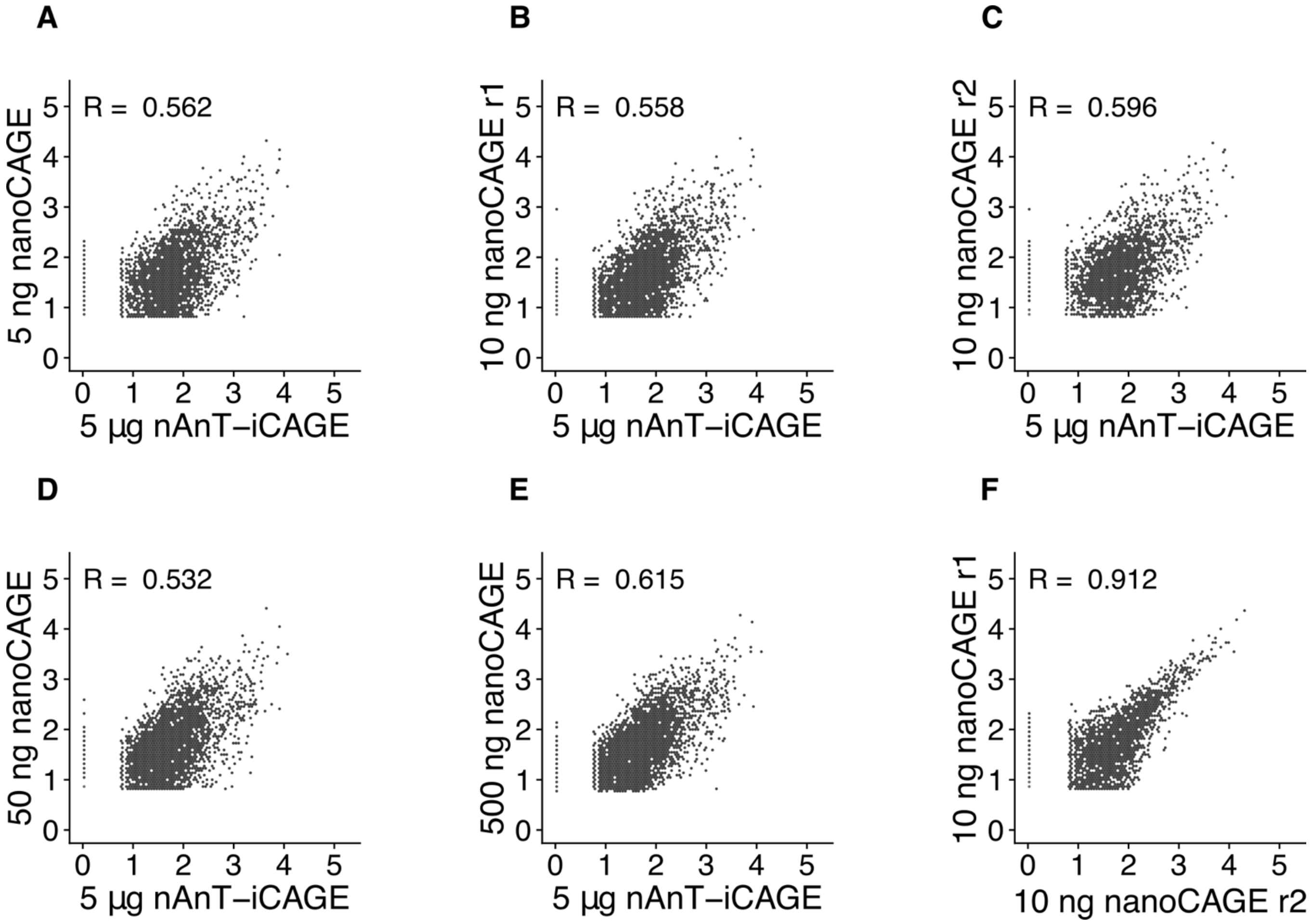

Supplemental Figure S23. Correlation of nanoCAGE and nAnT-iCAGE libraries on promoter/tag cluster level. Pearson correlation of tag cluster expression of nanoCAGE libraries derived from (**A**) 5 ng, (**B**) and (**C**) 10 ng, (**D**) 50 ng, (**E**) 500 ng of total RNA. (**F**) Correlation of nanoCAGE technical replicates derived from 10 ng of total RNA. The correlations are higher than when individual CTSS levels are compared (see Figure 3A-F), substantiating that the major nanoCAGE bias stems from template switching artefacts biasing

Supplemental Figure S24.

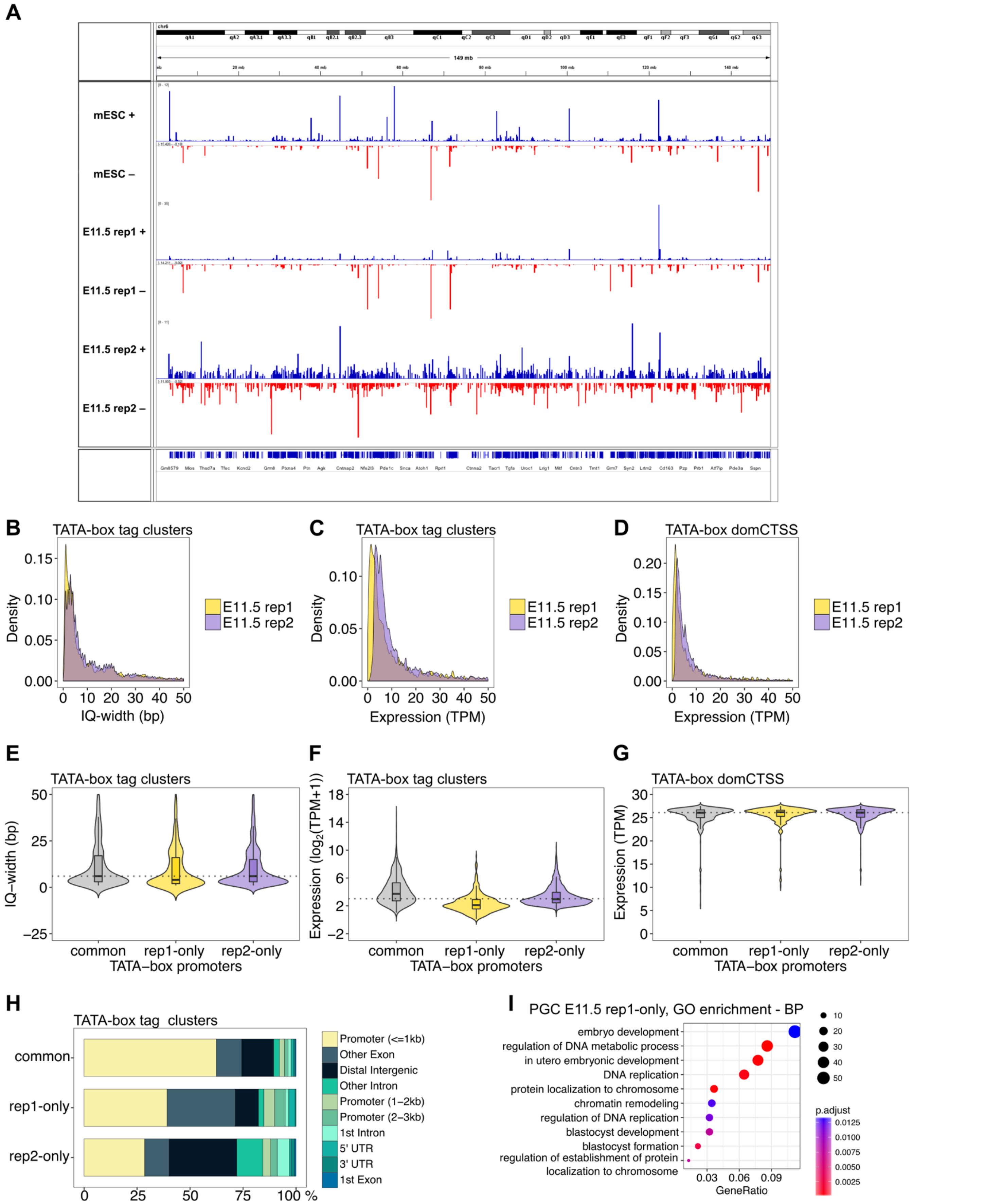

Supplemental Figure S24. Analyses of PGC E11.5 replicate 2. (**A**) Genome browser screenshot of chr6 reads from mESC E14 (nAnT-iCAGE) and PGC E11.5 replicate 1 or 2 (SLIC-CAGE). (**B**) IQ-width distribution of TATA-box promoters in E11.5 replicate 1 and 2. (**C**) TPM distribution of TATA-box promoters in E11.5 replicate 1 and 2. (**D**) TPM distribution of dominant CTSSs in TATA-box promoters. (**E**) IQ-width distribution of TATA-box promoters common to replicate 1 and 2 and specific for replicate 1 or 2. (**F**) TPM distribution of TATA-box promoters common to replicate 1 and 2 and specific for replicate 1 or 2. (**G**) TPM distribution of dominant CTSSs in TATA-box promoters common to replicate 1 and 2 and specific for replicate 1 or 2. (**H**) Genomic location of TATA-box tag clusters common to both replicate 1 and 2 or specific to replicate 1 or 2. (**I**) Biological process GO enrichment of TATA-box promoters specific to replicate 1.

Supplemental Figure S25.

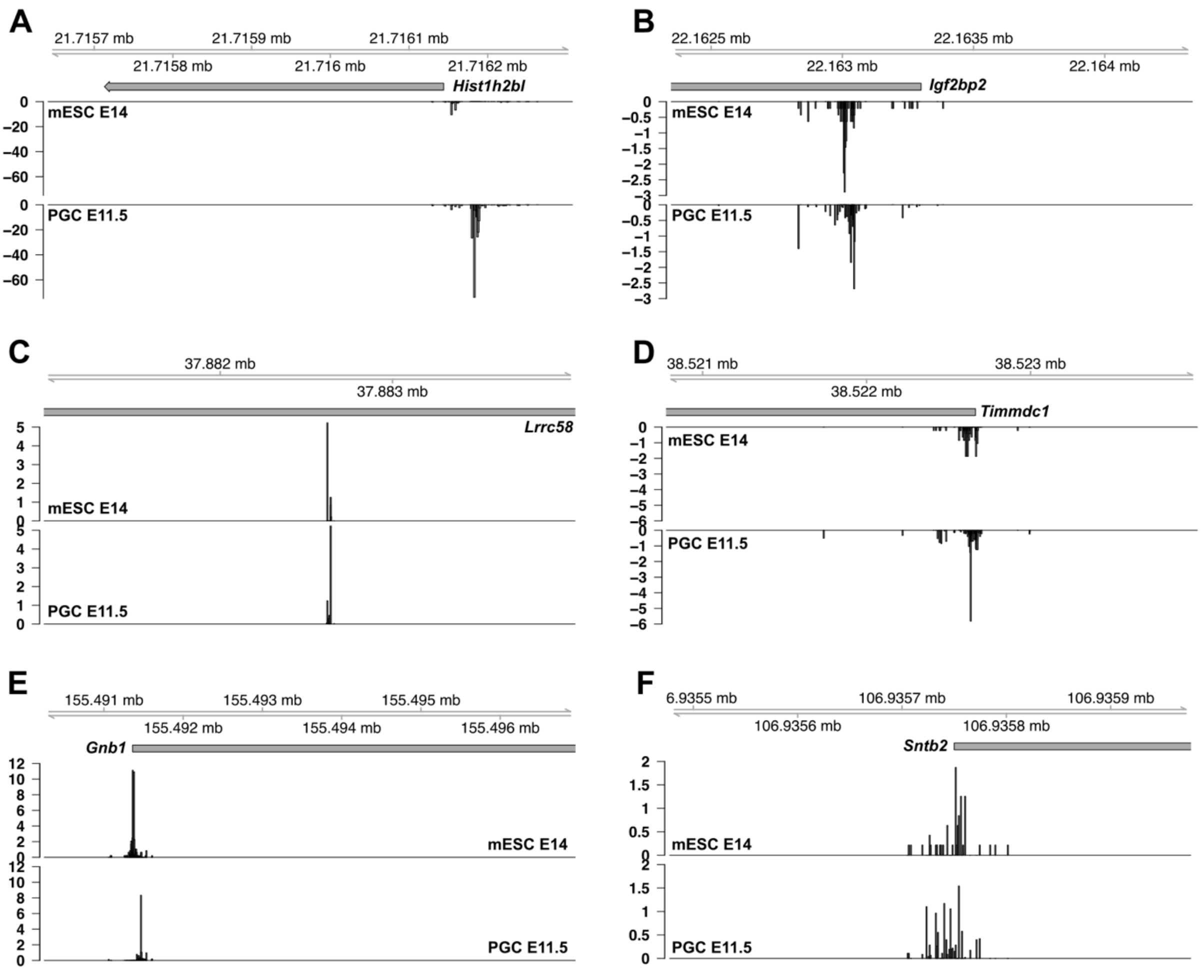

Supplemental Figure S25. CTSS signal in regions with TSS switching in PGC E11.5 compared to mESC E14. A) *Hist1h2bl* gene (29 bp shift in dominant CTSS position), **B)** *Igf2bp2* gene (36 bp shift in dominant CTSS position), **C)** *Lrrc58* gene (19 bp shift in dominant CTSS position), **D)** *Timmdc1* gene (21 bp shift in dominant CTSS position), **E)** *Gnb1* gene (104 bp shift in dominant CTSS position), **F)** *Sntb2* gene (3 bp shift in dominant CTSS position). These TSS switching events are identified using CAGEr Bioconductor package with stringent parameters for identification of shifting promoters (function *getShiftingPromoters*, parameters: tpmThreshold = 5, scoreThreshold = 0.6, fdrThreshold = 0.01).

SUPPLEMENTAL METHODS

Sample collection and nucleic acid extraction

S. cerevisiae BY4741 strain was grown in YPD media, and when the cells reached the exponential phase, collection was done by centrifugation. The cell material was stored at -80 degrees C prior to RNA isolation.

S. cerevisiae total RNA was extracted from using the standard hot-phenol procedure. The extracted RNA was additionally purified using the Qiagen RNeasy kit (clean-up protocol, according to manufacturer's instructions). Isolated RNA was quantified using NanoDrop1000 and the quality of RNA assessed on the bioanalyzer (Agilent). The RNA samples were of high quality (RIN > 9).

Mouse E14 cells were grown in in N2B27 (recipe below) supplemented with the inhibitors LIF (Millipore, ESG1107), CHIR99021 (Cambridge Bioscience, SM13-10) and PD-0325901 (Caltag-Medsystems Limited, SYN-1059). Cells were detached by trypsinization, collected by spinning down and frozen at -80 degrees C prior to RNA isolation.

Total RNA from mouse embryonic stem cells (E14 cell line) was extracted using Qiagen RNeasy kit (according to manufacturer's instructions). Isolated RNA was quantified using NanoDrop1000 and the quality of RNA assessed on the bioanalyzer (Agilent). The RNA samples were of high quality (RIN > 9).

Clustering of CTSSs into tag clusters and identification of dominant CTSS

CTSSs that pass the threshold of 1 TPM in at least one of the samples were clustered using a distance-based method implemented in the CAGEr package with a maximum allowed distance of 20 bp between the neighbouring CTSS.

For each tag cluster, a cumulative distribution of signal was calculated, and the boundaries of the tag cluster calculated using the 10th and 90th percentile of its signal. The distance between these boundaries represents the interquantile width of a tag cluster. The CTSS with the highest TPM value within a tag cluster is identified as the dominant CTSS (as implemented within CAGEr).

Genomic locations of tag clusters

Tag clusters were annotated with their corresponding genomic locations using the ChIPseeker package (Yu et al. 2015). In *S. cerevisiae* libraries, promoters were defined as 1 kb windows centred on Ensembl (Aken et al. 2016) annotated transcriptions start sites (annotations imported from SGD) and in M. musculus libraries, promoters were defined as <= 1 kb or 1-3 kb from the UCSC annotated transcription start site.

Nucleotide and dinucleotide composition of CTSSs

CTSSs from each library were filtered prior to analysis to include only CTSS with at least 1 TPM. In each library the number of A, C, G or T-containing CTSS was counted, divided by the total number of filtered CTSSs and converted to a percentage. The same analysis was performed using only dominant TSS (identified using the CAGEr package as a CTSS with highest expression within a tag cluster).

For dinucleotide analysis, identified filtered CTSSs were extended to include one upstream nucleotide ([-1, +1] dinucleotides where +1 represents the identified CTSS) and the same analysis as described above repeated for 16 possible dinucleotides.

ROC curves

To assess accuracy of TSS identification for SLIC-CAGE and nanoCAGE libraries, we used nAnT-iCAGE libraries to define the set of true CTSSs and tag clusters. A true positive CTSS or a tag cluster corresponds to the CTSS or tag cluster in the nAnT-iCAGE library, while a false positive CTSS or a tag cluster exists only in the nanoCAGE or SLIC-CAGE library. ROC curves were generated in dependence of the CTSS or tag cluster TPM threshold in nanoCAGE or SLIC-CAGE libraries.

Dinucleotide pattern analysis in *M. musculus* libraries

Heatmaps Bioconductor package (Perry M (18). heatmaps: Flexible Heatmaps for Functional Genomics and Sequence Features. R package version 1.2.0) was used to visualize dinucleotide patterns (TA and GC) across sequences centred on the dominant TSS. Sequences were ordered by interquantile width of the containing tag cluster, with the sharpest on top and broadest tag cluster on the bottom of the heatmap. Raw data with the exact matching for TA or GC was smoothed prior to plotting using kernel smoothing within the heatmaps package. Each heatmap was divided into two

sections based on tag cluster's IQ-widths. Empirical boundary (Supplemental Fig. S17A) was set to separate sharp (IQ-width <= 3 bp) and broad (IQ-width > 3) tag clusters identified in M. musculus libraries. The horizontal line/boundary was implemented using heatmaps options to partition heatmaps/rows of an image. Similarity of patterns between libraries was assessed by calculating the Jaccard distance between vectorized image matrices of smoothed heatmaps. Background similarity was assessed through calculation of Jaccard distance between vectorized image matrices of column-randomized, smoothed heatmaps. Column randomization was performed 10000 times, and the distribution of Jaccard distances calculated per each permutation was plotted and compared to the true Jaccard distance.

TATA-box motif analysis in *M. musculus* libraries

SeqPattern package was used to scan the sequences for the occurrence of the TATA-box motif using a threshold of 80th percentile match to the TATA-box PWM (imported from the seqPattern package). We further smoothed the obtained results using the kernel smoothing (heatmaps package) and plotted the results with sequences ordered by interquantile width of the containing tag cluster (sharpest on top and broadest on bottom of the tag cluster) and centred on the dominant TSS. The horizontal line in each heatmap represents the empirical boundary that separates sharp (IQ-width <= 3) and broad tag clusters (IQ-width > 3). Similarity between heatmaps was assessed as described above.

TATA-box metaplots (average signal/profile) were produced separately for sharp and broad tag clusters (see definition above). SeqPattern was used for scanning sequences using TATA-box PWM to identify 80% matches. The results were converted to the average signal using the heatmaps package with a 2 bp bin size. The final data was plotted using the ggplot2 package (Wickham 2009).

Nucleosome positioning signal in in *M. musculus* libraries – WW periodicity

WW dinucleotide (AA/AT/TA/TT) occurrence (average relative signal) was obtained using the heatmaps package separately for sharp and broad tag clusters (see definition above). A 2 bp bin size was used and the sequences were centred on the dominant TSS. As a control for the importance of centring the sequences on the dominant TSS, WW dinucleotide (AA/AT/TA/TT) occurrence was obtained as an average relative signal from sequences where each sequence is centred on a randomly

chosen CTSS within a tag cluster. The final data was plotted using the ggplot2 package (Wickham 2009).

H3K4me3 signal around *M. musculus* tag clusters

H3K4me3 data for E14 cell line, mapped to mm10 was downloaded from ENCODE experiment ENCSR000CGO. Bam files for two replicates (accession numbers ENCFF997CAQ and ENCFF425ZMWO) were merged using samtools (Li et al. 2009) and the merged bam file was imported to R environment using the rtracklayer package (Lawrence et al. 2009)

H3K4me3 coverage was calculated separately for reads mapping to minus or plus strand and minus strand reads subtracted from plus strand reads to get the subtracted H3K4me3 coverage.

Subtracted H3K4me3 coverage was visualized using heatmaps package centred on the dominant TSSs with sequences ordered by IQ-width of the containing tag clusters (sharpest on top, and broadest at the bottom of the heatmap). Each heatmap was divided into two sections based on tag cluster's IQ-widths as described above. Similarity between heatmaps was assessed as described above.

H3K4me3 coverage metaplots were produced separately for sharp and broad tag clusters (see definition above, only strongly supported dominant CTSSs with at least 5 TPM were used) using heatmaps package with a 3 bp bin size The final data was plotted using the ggplot2 package (Wickham 2009).

M. musculus tag cluster overlap with CpG islands

The CpG island track for mm10 was downloaded from the UCSC Genome Browser. Overlap with M. musculus tag clusters was visualized as a coverage heatmap using heatmaps package, centred on the dominant TSS with sequences ordered by IQ-width of the containing tag clusters (sharpest on top, and broadest at the bottom of the heatmap). Each heatmap was divided into two sections based on tag cluster's IQ-widths as described above.

CpG coverage metaplots were produced separately for sharp and broad tag clusters (see definition above) using heatmaps package with a 3 bp bin size. The final data was plotted using the ggplot2 package (Wickham 2009).

SUPPLEMENTAL RESULTS

SLIC-CAGE mapping efficiency

When only 1 ng of total RNA is used with a 5000-fold more carrier (5 µg), 25% of the sequenced reads are uniquely mapped to the target organism, while the rest corresponds to the leftover carrier (27%), short amplified linkers or multimappers, commonly discarded from TSS analyses (Supplemental Tables S10 and S11). This amount of leftover carrier is minor and does not significantly compromise sequencing depth (10% or less when 10 ng of total RNA are used). We expect that with additional rounds of degradation and purification, the leftover carrier could be further reduced, although with a risk of sample loss, and we found it unnecessary.

Analysis of nanoCAGE XL data

The nanoCAGE XL library exhibited low correlation with the nAnT-iCAGE library at individual CTSSs and tag clusters expression level (Supplemental Fig. S14A and B). Although distribution of interquantile widths suggests that libraries are not of low complexity (Supplemental Fig. S14D), this may be a consequence of contamination with non-capped captured RNA, as nanoCAGE XL poorly captures promoter regions (Supplementary Fig. S14E). In addition, only 7% of the dominant CTSSs identified in nanoCAGE XL libraries matched nAnT-iCAGE identified dominant CTSSs, while CTSS and initiator biases were even more prominent (Supplemental Fig. S14C and G) than in our dataset.

Additional comparison of PGC E11.5 replicate 1 and 2

Comparison of TATA-box and CpG heatmaps in Fig. 5 and Supplemental Fig. S18G shows that more TATA-box promoters and fewer CpG island-associated promoters are identified in PGC E11.5 replicate 2. Replicate 2 is derived from degraded RNA (see Supplemental Fig. S18A) and the higher background noise (Supplemental Fig. S24A) may increase the total signal level of the identified tag clusters. This is expected to have a greater influence on TATA-box promoters as they typically have precise transcription initiation resulting with sharp tag clusters (Haberle and Lenhard 2016). Lowly expressed CTSSs and tag clusters are typically excluded from analysis by applying a filtering threshold within the standard CAGEr pipeline (Haberle et al. 2015). Higher background noise in the data would increase the width and the total signal level of tag clusters, allowing a subset of lowly

expressed tag clusters to pass the threshold and stay included in the dataset, while without the noise, they would be excluded. To see if this is the case with TATA-box promoters detected only in replicate 2, we compared expression levels and IQ-widths of PGC E11.5 replicate 1 and 2 (Supplemental Fig. S24B,C, TATA-box promoters are identified as those that pass the threshold of minimum 80th percentile of maximum TATA pwm score at -40-20 bp positions). Indeed, TATA-box promoters are broader and of higher expression levels in replicate 2 than in replicate 1. When dominant TSSs are compared (Supplemental Fig. S24D), the difference in expression levels is much smaller, indicating that the background noise is raising expression levels and IQ-width of TATA-box promoters in replicate 2. Further, we classified TATA-box promoters into those that are common to both replicate 1 and 2 and found in only in replicate 1 or 2. We compared IQ-width, total expression levels and expression levels of dominant TSSs identified in those TATA-box classes (Supplemental Fig. S24E-G). Indeed, TATA-box promoters specific for replicate 2 are broader and more expressed than replicate 1-specific TATA-box promoters, while dominant TSSs are highly similar. Further, genomic locations of replicate 2 tag clusters with identified TATA-boxes are in high percentage in distal intergenic regions, demonstrating again higher noise and lower specificity. GO enrichment analysis showed significant terms only for TATA-box promoters specific for PGC E11.5 replicate 1, while there was no enrichment for TATA-box promoters specific for replicate 2, again showing biological relevance of replicate 1 and noise in replicate 2.

SUPPLEMENTAL REFERENCES

Adiconis X, Haber AL, Simmons SK, Levy Moonshine A, Ji Z, Busby MA, Shi X, Jacques J, Lancaster MA, Pan JQ et al. 2018. Comprehensive comparative analysis of 5'-end RNA-sequencing methods. *Nat Methods* doi:10.1038/s41592-018-0014-2.

Haberle V, Forrest AR, Hayashizaki Y, Carninci P, Lenhard B. 2015. CAGEr: precise TSS data retrieval and high-resolution promoterome mining for integrative analyses. *Nucleic acids research* **43**: e51.

Haberle V, Lenhard B. 2016. Promoter architectures and developmental gene regulation. *Semin Cell Dev Biol* **57**: 11-23.

Poulain S, Kato S, Arnaud O, Morlighem JE, Suzuki M, Plessy C, Harbers M. 2017. NanoCAGE: A Method for the Analysis of Coding and Noncoding 5'-Capped Transcriptomes. *Methods Mol Biol* **1543**: 57-109.
